# Synthesis and Biological Evaluation of New *cis*-Restricted Triazole Analogues of Combretastatin A-4

**DOI:** 10.3390/molecules30020317

**Published:** 2025-01-15

**Authors:** Lidia Prieto, Daniel Gaviña, Marcos Escolano, María Cánovas-Belchí, María Sánchez-Roselló, Carlos del Pozo, Eva Falomir, Santiago Díaz-Oltra

**Affiliations:** 1 Department of Organic Chemistry, University of Valencia, E-46100 Burjassot, Spain; lidia.prieto@uv.es (L.P.); daniel.gavina@uv.es (D.G.); marcos.escolano@uv.es (M.E.); macabel6@alumni.uv.es (M.C.-B.); maria.sanchez-rosello@uv.es (M.S.-R.); carlos.pozo@uv.es (C.d.P.); 2 Department of Organic and Inorganic Chemistry, University Jaume I, E-12071 Castellón, Spain; efalomir@uji.es

**Keywords:** cancer, tubulin, antimitotics, combretastatin A-4, triazoles, cytotoxicity

## Abstract

The natural products combretastatins A-1 and A-4 are potent antimitotic and vascular-disrupting agents through their binding at the colchicine site in tubulin. However, these compounds suffer from a low water solubility and a tendency to isomerize to the inactive *trans* stilbenes. In this study, we have prepared a series of 18 *cis*-restricted triazole analogues of combretastatin A-4 (CA-4), maintaining, in all cases, the 3,4,5-trimethoxy phenyl ring A, with the aim of investigating the substitution pattern on the B-ring in a systematic way. To this end, cytotoxic activities of the *cis*-restricted analogues of CA-4 prepared were determined in two tumor cell lines, namely, HT-29 and A-549, as well as in the non-tumor cell line HEK-293, to pre-evaluate the selectivity profile of the compounds for the tumor cell lines. The main conclusion was the essential presence of methoxyl or ethoxyl groups at the *para* position of the B-ring in order to obtain good antitumor activities. Thus, the more active compounds in our study displayed IC_50_ values in the nanomolar range for the tumor cell lines but not for the normal cells. Consequently, these triazole analogues of CA-4 could serve as promising alternatives to the natural product, although further studies about their biological activity are essential in order to fully determine their viability as therapeutic agents in the treatment of cancer.

## 1. Introduction

Despite considerable progress in its early diagnosis and treatment, cancer continues to be a major health and socio-economic problem, accounting for millions of deaths annually [1]. The search for new anti-cancer drugs and the development of more effective treatment strategies is a field of utmost importance in the current drug discovery and clinical research. While there has been significant progress in our understanding of the molecular mechanisms underlying this diverse group of diseases collectively referred to as ‘cancer’ (there are in fact more than 150 different types of cancer), the development of improved anti-cancer drugs is of pressing urgency.

Cytotoxic drugs work by interfering with the cell’s ability to divide and proliferate, ultimately leading to cell death [2,3,4,5,6]. Many of these drugs interfere with microtubule dynamics by its interaction with tubulin, a protein that serves as the building block for microtubules, like the well-known antimitotic drugs taxanes or vinca alkaloids [7].

**Figure 1:**
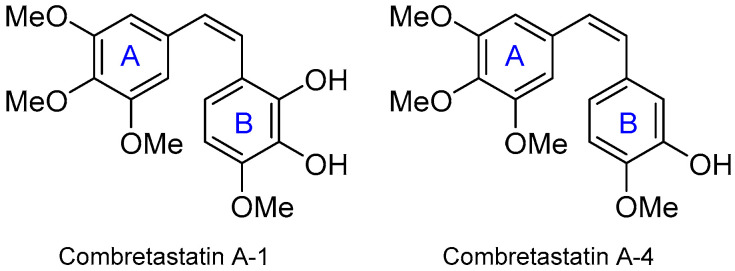
Structures of vascular-disrupting agents combretastatins A-1 and A-4.

Another important family of compounds that interact with tubulin are combretastatins (Figure 1) [8], which bind to the colchicine domain of tubulin. Particularly, combretastatin A-1, A-4, and their derivatives, have garnered significant interest in cancer research due to their potent ability to disrupt tumor vasculature leading to tumor regression and necrosis [7,9]. Extensive structure–activity relationships (SAR) studies on combretastatin A-4 (CA-4) have shown that the 3,4,5-trimethoxy ring A is essential in order to anchor the molecule to the target, as well as the *cis* configuration of the double bond and the 4′-methoxy group at ring B (Figure 1) [9,10,11,12].

Some common drawbacks of the combretastatin family of compounds are their poor water solubility and their tendency to isomerize to the inactive *trans* stilbenes. To improve water solubility, they have been used as phosphate ester prodrugs in clinical trials; however, in order to avoid the *cis*–*trans* isomerization, some perpetual structural modifications have to be carried out. One approach to obtaining *cis*-restricted analogues of CA-4 involves replacing the carbon–carbon double bond with four- or five-membered heterocyclic rings, which not only restrict the geometry but also could enhance water solubility through the incorporation of more heteroatoms [9,11].

**Figure 2:**
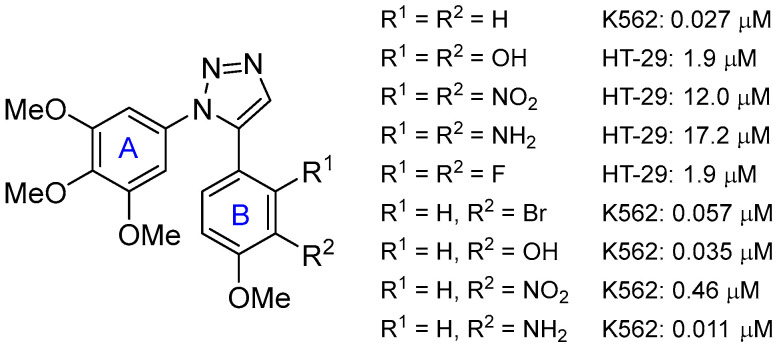
Known 1,5-disubsituted 1,2,3-triazole analogues of CA-4, with IC_50_ values available on the cell line indicated.

Between the different heterocyclic rings that can be used to this end, 1,2,3-triazoles are a class of heterocyclic compounds widely recognized for their versatility in medicinal chemistry. These structures are characterized by their aromaticity, metabolic stability, and resistance to enzymatic degradation, making them valuable pharmacophores in drug design [13,14]. Among these, some 1,5-disubstituted 1,2,3-triazole analogues of CA-4 have shown promising biological activities (Figure 2) [15,16,17,18,19,20], and it has been proposed by molecular modeling that their binding site coincides with that of the parent molecule [15,16,17,18].

The more active compounds of this family are analogues with R^1^ = H and R^2^ = H, Br, OH, or NH_2_ (Figure 2), displaying cytotoxic activities in the nanomolar range against the K562 leukemia cancer cell line [15]. However, SAR studies for this family of compounds are incomplete since the substitution pattern of B-ring has not been extensively studied as has been done with CA-4.

In the present work, we proposed to broaden the chemical space previously covered for the B-ring substitution pattern of triazole CA-4 analogues in a systematic way. Therefore, we have synthesized a new series of 1,5-disubstituted 1,2,3-triazole derivatives and we have determined their cytotoxicity in two tumor cell lines, namely, the human colon cancer cell line HT-29 and human lung cancer cell line A-549, as well as non-tumor human embryonic kidney 293 cells (HEK-293), as a means of comparison to pre-evaluate the selectivity profile of the newly obtained compounds for the tumor cell lines [21,22,23].

## 2. Results

### 2.1. Chemical Synthesis

**Scheme 1:**
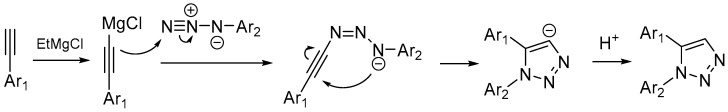
Proposed mechanism for the synthesis of 1,5-disubstituted 1,2,3-triazoles.

The thermally induced Huisgen 1,3-dipolar cycloaddition of aromatic alkynes and aryl azides usually affords mixtures of regioisomeric 1,4- and 1,5-disubstituted 1,2,3-triazoles. The regioselective formation of the 1,5-disubstituted triazoles was originally described by Sharpless [24] and is explained by the mechanism illustrated in Scheme 1. In this process, terminal alkynes react with EtMgCl to generate magnesium acetylides. The nucleophilic addition of these intermediates exclusively to the terminal nitrogen atom of the azide accounts for the observed regioselectivity. This step is followed by ring closure and protonation, resulting in the formation of 1,5-disubstituted triazoles (Scheme 1).

**Scheme 2:**
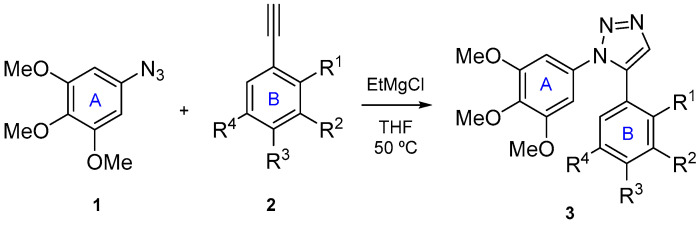
General strategy for the synthesis of 1,5-disubstituted 1,2,3-triazole analogues of CA-4.

Accordingly, our general strategy to selectively obtain the 1,5-geometry of the two aryl groups in triazoles **3** was to react A-ring azide **1** with B-ring magnesium acetylides of terminal alkynes **2**, as previously reported (Scheme 2) [15,17,18].

**Scheme 3:**
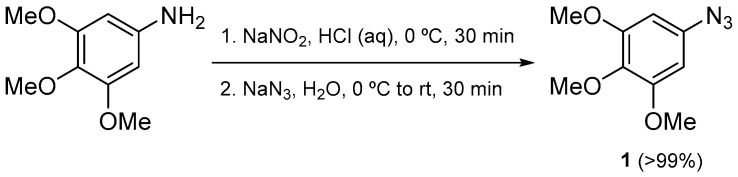
Synthesis of 3,4,5-trimethoxyaryl azide **1**.

On the one hand, 3,4,5-trimethoxyaryl azide **1** was prepared, in a quantitative yield, from the corresponding aniline by a two-step one-pot procedure, consisting of a standard diazotization followed by the displacement of the resulting diazo group with sodium azide (Scheme 3) [25].

On the other hand, B-ring terminal alkynes **2** that were not commercially available were obtained by the homologation of the corresponding aromatic aldehydes **4** using the Colvin rearrangement, with the lithium salt of trimethylsilyldiazomethane as a homologation reagent. This reaction took place in moderate to good yields (Table 1) [26,27,28]. When appropriate, phenolic hydroxyls of commercial aldehydes were conveniently alkylated with methyl or ethyl iodide to obtain aldehydes **4a** and **4d**, respectively, or protected as *tert*-butyldimethylsilyl ether (OTBS) to obtain aldehydes **4b**, **4c**, and **4e** (see SI for details).

The last step of our synthesis was the coupling of azide **1** with aromatic alkynes **2**, as well as other commercially available terminal alkynes. To this end, Huisgen cycloaddition took place through the metalation of the terminal alkynes **2** with ethyl magnesium chloride, followed by the addition of azide **1**. This reaction gave rise to the desired 1,5-disubstituted triazoles with complete regioselectivity in moderate to good yields (Table 2).

### 2.2. Biological Evaluation

Cytotoxicity of the series of *cis*-restricted triazole analogues of CA-4 was investigated as described in the Materials and Methods section by growth inhibition assays, using two types of tumor cells, namely, the human colon cancer cell line HT-29 and human lung cancer cell line A-549, as well as one normal cell line, the human embryonic kidney cell line HEK-293 [29], as a way to estimate the selectivity of the compounds for tumor cells. Cytotoxicity values, expressed as the compound concentration (μM) that causes a 50% inhibition of cell growth (IC_50_) after 48 h of incubation, are shown in Table 3.

Only two of the analogues synthesized were previously studied, **3i** and **3r** [15,16,18,19,20]; however, they have not been investigated with the cell lines employed in this work, so they were prepared again to compare their activity with the previous one.

Triazole derivative **3a**, with no substitution on the B-ring, was found to be non-cytotoxic in the concentration range tested for any cell line, as well as compounds mono-substituted at the *ortho* position with bromine (**3b**) or nitro groups (**3c**) (Table 3). Likewise, triazoles monosubsituted at the *para* position of the phenyl B-ring with methyl (**3d**), triflouromethyl (**3e**), fluorine (**3f**), chlorine (**3g**), and bromine (**3h**) groups were either non-cytotoxic or weakly cytotoxic. Conversely, analogues monosubstituted at the *para* position with methoxyl (**3i**) or ethoxyl (**3j**) groups turned out to be highly cytotoxic and very selective for the tumor cell lines. Thus, compound **3i**, that was already described to have an IC_50_ value of 27 nM for the K562 leukemia cancer cell line (Figure 2, R^1^ = R^2^ = H) [15], showed similar values for HT-29 colon cancer and A-549 lung cancer cell lines (20 and 22 nM, respectively) and, gratifyingly, it was three orders of magnitude less cytotoxic for the non-tumor HEK-293 cell line (35 μM). Curiously, triazole **3j** showed high selectivity for the colon cancer HT-29 cells (being the most active one for this cell line, with an IC_50_ value of 2 nM); however, it was much less cytotoxic for the lung cancer A-549 cell line (5.8 μM).

Regarding *cis*-restricted analogues of CA-4 disubstituted on the phenyl B-ring, the presence of bromine at the *ortho* position (compounds **3k** and **3l**), drastically reduced the cytotoxicity. More surprisingly, triazole **3m**, bearing a bromine atom at the *meta* position and an ethoxyl group in *para*, was weakly cytotoxic (Table 3). It is worth mentioning that its analogue with bromine in *meta* and methoxyl in *para* (not prepared) was described to have nanomolar activity for the K562 leukemia cancer cell line (Figure 2, R^1^ = H, R^2^ = Br) [15].

The best cytotoxic activities were shown by triazole analogues combining a methoxyl group at the *para* position of the phenyl B-ring with a chlorine atom (**3n**) or a hydroxyl group (**3r**) at the *meta* position. Thus, compound **3n** was the most active one against the A-549 lung cancer cell line, with an IC_50_ of 3 nM; however, it is also the most cytotoxic compound for the non-tumor cell line HEK-293 (25 nM), so it is less selective than **3r**, for instance.

The incorporation of two methoxyl groups, at the *meta* and *para* positions, completely eliminated the cytotoxic activity of the corresponding CA-4 analogue **3o**. The same result was observed when the methoxyl group in *para* was substituted for a hydroxyl group, like in compounds **3p** and **3q**.

Finally, analogue **3r**, that shares substitution pattern on the B-ring with the reference natural product CA-4, was previously described to have an IC_50_ of 35 nM for the leukemia cancer cell line K562 (Figure 2, R^1^ = H, R^2^ = OH) [15]. In our study, this compound was the second most active for the A-549 cell line (IC_50_ = 9 nM) and was very selective for tumor cells.

## 3. Discussion

In summary, we have efficiently synthesized a series of 1,5-disubstituted triazoles (**3**) by means of a regioselective 1,3-dipolar Huisgen cycloaddition between aromatic alkynes (**2**), in turn prepared through the Colvin rearrangement reaction from aldehydes **4**, and 3,4,5-trimethoxyaryl azide (**1**).

These new *cis*-restricted triazole analogues of CA-4 (**3**) allowed us to evaluate how the structural modifications on the B-ring moiety affect the biological activity of those derivatives, providing valuable information on the structure–activity relationship.

In this context, the main conclusion was the essential presence of methoxyl or ethoxyl groups at the *para* position of the B-ring in order to improve the antitumor activities of the parent molecule CA-4, like in compounds **3i** and **3j**. The later, bearing an ethoxyl group, was the most active one for the human colon cancer HT-29 cell line (IC_50_ = 2 nM), and very selective when compared with the cytotoxicity on the non-tumor cell line.

Moreover, when these methoxyl or ethoxyl groups at the *para* position were combined with another substituent on the B-ring, the best results were obtained with triazoles containing chlorine (**3n**) or hydroxyl (**3r**) groups at the *meta* position. Thus, compound **3n** turned out to be the most active one against the lung cancer A-549 cell line (IC_50_ = 3 nM), albeit less selective for tumor cells than **3r**, which was the second most active for the lung cancer cell line (IC_50_ = 9 nM) and very selective for tumor cells.

Computational studies of compound **3r** [16,18], along with other triazole CA-4 analogues depicted in Figure 2 [15,17], have consistently predicted that their binding mode is analogous to that of CA-4 at the colchicine site of tubulin. Consequently, the potent cytotoxic activity observed for compounds **3i**, **3j**, **3n**, and **3r** is presumed to arise from the same mechanism of action.

These preliminary investigations suggest that *cis*-restricted triazole analogues **3i**, **3j**, **3n,** and **3r**, in particular, could serve as promising alternatives to the original CA-4, although further studies about their biological activity are essential in order to fully determine their viability as therapeutic agents in the treatment of cancer.

## 4. Materials and Methods

### 4.1. Chemistry

**General procedure of the 1,3-dipolar Huisgen cycloaddition for the synthesis of triazoles.** To the corresponding terminal alkyne **2** (0.5 mmol, 1 equiv.) in dry THF (1.10 M), EtMgCl 2 M in THF (1.25 equiv.) was added dropwise at room temperature under N_2_ atmosphere. Upon completion of the addition, the solution was heated to 50 °C for 15 min and cooled to room temperature. Then, a solution of the azide **1** in THF (0.45 M, 1 equiv.) was loaded dropwise. The reaction mixture was heated to 50 °C for 1.5 h. After quenching with saturated aqueous NH_4_Cl, the product was extracted 3 times with CH_2_Cl_2_. The organic phase was dried over anhydrous Na_2_SO_4_ and the mixture was concentrated to dryness and purified by flash column chromatography on silica gel using mixtures of *n*-hexane and ethyl acetate as eluents.
**5-phenyl-1-(3,4,5-trimethoxyphenyl)-1H-1,2,3-triazole (3a):** Starting from ethynylbenzene (51 mg, 0.5 mmol) and following the general procedure described before, compound **3a** was purified by chromatography, eluting with *n*-hexane-EtOAc (2:1). White solid (0.11 g, 0.36 mmol), yield 72%. NMR spectra match with previously published [31].
**5-(2-bromophenyl)-1-(3,4,5-trimethoxyphenyl)-1H-1,2,3-triazole (3b)**. Starting from 1-bromo-2-ethynylbenzene (90 mg, 0.5 mmol) and following the general procedure described before, compound **3b** was purified by chromatography, eluting *n*-hexane-EtOAc (2:1). Brown solid (0.17 g, 0.43 mmol), yield 85%, mp 120–122 °C. ^1^H NMR (300 MHz, CDCl_3_) δ 7.85 (s, 1H), 7.67 (dd, *J* = 7.8, 1.5 Hz, 1H), 7.39–7.28 (m, 2H), 7.25–7.22 (m, 1H), 6.56 (s, 2H), 3.83 (s, 3H), 3.67 (s, 6H). ^13^C NMR (75 MHz, CDCl_3_) δ 153.4, 138.3, 136.3, 134.5, 133.4, 132.2, 132.1, 131.4, 129.3, 127.8, 124.6, 101.7, 61.1, 56.2. HRMS (ESI/Q-TOF): *m/z* [M+H]^+^ calcd. for C_17_H_17_BrN_3_O_3_^+^ [M+H]^+^: 390.0448, found: 390.0438.
**5-(2-nitrophenyl)-1-(3,4,5-trimethoxyphenyl)-1H-1,2,3-triazole (3c):** Starting from 1-ethynyl-2-nitrobenzene (74 mg, 0.5 mmol) and following the general procedure described before, compound **3c** was purified by chromatography, eluting with *n*-hexane-EtOAc (1:1). Brown solid (0.11 g, 0.30 mmol), yield 59%, mp 122–124 °C. ^1^H NMR (300 MHz, CDCl_3_) δ 8.06 (dd, *J* = 8.1, 1.2 Hz, 1H), 7.82 (s, 1H), 7.74–7.60 (m, 2H), 7.44 (dd, *J* = 7.5, 1.6 Hz, 1H), 6.49 (s, 2H), 3.83 (s, 3H), 3.67 (s, 6H).^13^C NMR (75 MHz, CDCl_3_) δ 153.6, 148.6, 138.8, 133.8, 133.6, 132.9, 131.5, 131.1, 125.2, 122.9, 102.3, 61.1, 56.3. HRMS (ESI/Q-TOF): *m/z* [M+H]^+^ calcd. for C_17_H_17_N_4_O_5_^+^ [M+H]^+^: 357.1193, found: 357.1183.
**5-(p-tolyl)-1-(3,4,5-trimethoxyphenyl)-1H-1,2,3-triazole (3d):** Starting from 1-ethynyl-4-methylbenzene (58 mg, 0.5 mmol) and following general procedure D, compound **3d** was purified by chromatography, eluting with *n*-hexane-EtOAc (1:1). Green solid (98 mg, 0.30 mmol), yield 60%, mp 127–129 °C. ^1^H NMR (300 MHz, CDCl_3_) δ 7.82 (s, 1H), 7.19–7.13 (m, 4H), 6.57 (s, 2H), 3.87 (s, 3H), 3.71 (s, 6H), 2.36 (s, 3H). ^13^C NMR (75 MHz, CDCl_3_) δ 153.6, 139.6, 138.6, 137.9, 133.2, 132.3, 129.7, 128.6, 124.0, 102.9, 61.2, 56.3, 21.4. HRMS (ESI/Q-TOF): *m/z* [M+H]^+^ calcd. for C_18_H_20_N_3_O_3_^+^ [M+H]^+^: 326.1499, found: 326.1502.
**5-(4-(trifluoromethyl)phenyl)-1-(3,4,5-trimethoxyphenyl)-1H-1,2,3-triazole (3e):** Starting from 1-ethynyl-4-(trifluoromethyl)benzene (85 mg, 0.5 mmol) and following general procedure D, compound **3e** was purified by chromatography, eluting with *n*-hexane-EtOAc (2:1). Orange solid (0.12 g, 0.31 mmol), yield 61%, mp 101–103 °C. ^1^H NMR (300 MHz, CDCl_3_) δ 7.84 (s, 1H), 7.58 (d, *J* = 8.1 Hz, 2H), 7.37 (d, *J* = 8.1 Hz, 2H), 6.47 (s, 2H), 3.79 (s, 3H), 3.63 (s, 6H). ^13^C NMR (75 MHz, CDCl_3_) δ 153.6, 138.8, 136.3, 133.6, 131.75, 131.6, 131.3, 130.9, 130.4 (q, *J* = 1.2 Hz), 128.9, 125.8 (q, *J* = 12.3 Hz), 123.6 (q, *J* = 272.2 Hz), 102.8, 60.9, 56.2. ^19^F NMR (282 MHz, CDCl_3_) δ -63.4. HRMS (ESI/Q-TOF): *m/z* [M+H]^+^ calcd. for C_18_H_17_F_3_N_3_O_3_^+^ [M+H]^+^: 380.1217, found 380.1224.
**5-(4-fluorophenyl)-1-(3,4,5-trimethoxyphenyl)-1H-1,2,3-triazole (3f):** Starting from 1-ethynyl-4-fluorobenzene (60 mg, 0.5 mmol) and following general procedure D, compound **3f** was purified by chromatography, eluting with *n*-hexane-EtOAc (2:1). Yellow solid (84 mg, 0.26 mmol), yield 51%, mp 129–131 °C. ^1^H NMR (300 MHz, CDCl_3_) δ 7.82 (s, 1H), 7.47–7.43 (m, 2H), 7.29–7.23 (m, 2H), 6.74 (s, 2H), 4.05 (s, 3H), 3.90 (s, 6H). ^13^C NMR (75 MHz, CDCl_3_) δ 163.0 (d, *J* = 250.7 Hz), 153.6, 138.7, 136.8, 133.3, 131.9, 130.6 (d, *J* = 8.5 Hz), 123.0 (d, *J* = 3.5 Hz), 116.2 (d, *J* = 22.0 Hz), 102.8, 61.1, 56.3. ^19^F NMR (282 MHz, CDCl_3_) δ -111.2. HRMS (ESI/Q-TOF): *m/z* [M+H]^+^ calcd. for C_17_H_17_FN_3_O_3_^+^ [M+H]^+^: 330.1248, found: 330.1256.
**5-(4-chlorophenyl)-1-(3,4,5-trimethoxyphenyl)-1H-1,2,3-triazole (3g):** Starting from 1-chloro-4-ethynylbenzene (68 mg, 0.5 mmol) and following general procedure D, compound **3g** was purified by chromatography, eluting with *n*-hexane-EtOAc (2:1). White solid (89 mg, 0.28 mmol), yield 51%, mp 146–148 °C. ^1^H NMR (300 MHz, CDCl_3_) δ 7.86 (s, 1H), 7.36 (d, *J* = 8.6 Hz, 2H), 7.21 (d, *J* = 8.6 Hz, 2H), 6.55 (s, 2H), 3.89 (s, 3H), 3.74 (s, 6H). ^13^C NMR (75 MHz, CDCl_3_) δ 153.7, 138.9, 136.7, 135.7, 133.5, 132.0, 129.9, 129.4, 125.4, 103.0, 61.2, 56.4. HRMS (ESI/Q-TOF): *m/z* [M+H]^+^ calcd. for C_17_H_17_ClN_3_O_3_^+^ [M+H]^+^: 346.0953, found: 346.0956.
**5-(4-bromophenyl)-1-(3,4,5-trimethoxyphenyl)-1H-1,2,3-triazole (3h):** Starting from 1-bromo-4-ethynylbenzene (91 mg, 0.5 mmol) and following general procedure D, compound **3h** was purified by chromatography, eluting *n*-hexane-EtOAc (2:1). Green solid (0.13 mg, 0.33 mmol), yield 66%, mp 163–165 °C. ^1^H NMR (300 MHz, CDCl_3_) δ 7.86 (s, 1H), 7.52 (d, *J* = 8.6 Hz, 1H), 7.15 (d, *J* = 8.6 Hz, 2H), 6.55 (s, 2H), 3.89 (s, 3H), 3.74 (s, 6H). ^13^C NMR (75 MHz, CDCl_3_) δ 153.8, 138.9, 136.8, 133.5, 132.3, 131.9, 130.1, 125.9, 123.9, 103.0, 61.2, 56.4. HRMS (ESI/Q-TOF): *m/z* [M+H]^+^ calcd. for C_17_H_17_BrN_3_O_3_^+^ [MH]^+^: 390.0448, found: 390.0432.
**5-(4-methoxyphenyl)-1-(3,4,5-trimethoxyphenyl)-1H-1,2,3-triazole (3i):** Starting from 1-ethynyl-4-methoxybenzene (66 mg, 0.5 mmol) and following general procedure D, compound **3i** was purified by chromatography, eluting with *n*-hexane-EtOAc (2:1). White solid (0.10 mg, 0.30 mmol), yield 60%. NMR spectra match with previously published [15].
**5-(4-ethoxyphenyl)-1-(3,4,5-trimethoxyphenyl)-1H-1,2,3-triazole (3j):** Starting from **2g** (73 mg, 0.5 mmol) and following general procedure D, compound **3j** was purified by chromatography, eluting with *n*-hexane-EtOAc (2:1). Orange solid (91 mg, 0.26 mmol), yield 51%, mp 102–104 °C ^1^H NMR (300 MHz, CDCl_3_) δ 7.78 (s, 1H), 7.17 (d, *J* = 9.0 Hz, 2H), 6.86 (d, *J* = 9.0 Hz, 2H), 6.57 (s, 2H), 4.02 (q, *J* = 7.0 Hz, 2H), 3.86 (s, 3H), 3.71 (s, 6H), 1.40 (t, *J* = 7.0 Hz, 3H). ^13^C NMR (75 MHz, CDCl_3_) δ 159.8, 153.6, 138.5, 137.7, 133.0, 132.4, 130.0, 118.8, 114.9, 102.9, 63.7, 61.1, 56.3, 14.8. HRMS (ESI/Q-TOF): *m/z* [M+H]^+^ calcd. for C_19_H_21_N_3_O_4_^+^ [M+H]^+^: 356.1605, found: 356.1597.
**5-(2-bromo-4-methoxyphenyl)-1-(3,4,5-trimethoxyphenyl)-1H-1,2,3-triazole (3k):** Starting from **2h** (0.11 g, 0.5 mmol) and following general procedure D, compound **3k** was purified by chromatography, eluting with *n*-hexane-EtOAc (1:1). Yellow solid (0.12 g, 0.28 mmol), yield 56%, mp 128–130 °C. ^1^H NMR (300 MHz, CDCl_3_) δ 7.80 (s, 1H), 7.19 (d, *J* = 2.6 Hz, 1H), 7.11 (d, *J* = 8.6 Hz, 1H), 6.87 (dd, *J* = 8.6, 2.6 Hz, 1H), 6.57 (s, 2H), 3.83 (s, 3H), 3.82 (s, 3H), 3.69 (s, 6H). ^13^C NMR (75 MHz, CDCl_3_) δ 161.2, 153.4, 138.3, 136.3, 135.0, 132.8, 132.2, 125.1, 120.9, 118.6, 113.9, 101.8, 61.3, 56.3, 55.9. HRMS (ESI/Q-TOF): *m/z* [M+H]^+^ calcd. for C_187_H_19_BrN_3_O4^+^ [M+H]^+^: 420.0553, found: 420.0542.
**5-(2-bromo-5-methoxyphenyl)-1-(3,4,5-trimethoxyphenyl)-1H-1,2,3-triazole (3l):** Starting from **2i** (0.11 g, 0.5 mmol) and following general procedure D, compound **3l** was purified by chromatography, eluting with *n*-hexane-EtOAc (2:1). White solid (76 mg, 0.19 mmol), yield 38%, mp 158–160 °C. ^1^H NMR (300 MHz, CDCl_3_) δ 7.83 (s, 1H), 7.52 (d, *J* = 8.9 Hz, 1H), 6.85 (dd, *J* = 8.9, 3.0 Hz, 1H), 6.75 (d, *J* = 3.0 Hz, 1H), 6.60 (s, 2H), 3.83 (s, 3H), 3.73 (s, 3H), 3.69 (s, 6H). ^13^C NMR (75 MHz, CDCl_3_): δ = 159.0, 153.5, 138.4, 134.0, 130.2, 117.9, 117.3, 115.0, 101.8, 61.1, 56.3, 55.8. HRMS (ESI/Q-TOF): *m/z* [M+H]^+^ calcd. for C_18_H_19_BrN_3_O_4_^+^ [M+H]^+^: 420.0553, found: 420.0534.
**5-(3-bromo-4-ethoxyphenyl)-1-(3,4,5-trimethoxyphenyl)-1H-1,2,3-triazole (3m):** Starting from **2d** (0.11 g, 0.5 mmol) and following general procedure D, compound **3m** was purified by chromatography, eluting with *n*-hexane-EtOAc (1:1). Green solid (0.11 g, 0.25 mmol), yield 51%, mp 130–132 °C. ^1^H NMR (400 MHz, CDCl_3_) δ 7.74 (s, 1H), 7.47 (d, *J* = 2.2 Hz, 1H), 7.04 (dd, *J* = 8.6, 2.2 Hz, 1H), 6.79 (d, *J* = 8.6 Hz, 1H), 6.53 (s, 2H), 4.04 (q, *J* = 7.0 Hz, 2H), 3.82 (s, 3H), 3.69 (s, 6H), 1.41 (t, *J* = 7.0 Hz, 3H). ^13^C NMR (101 MHz, CDCl_3_) δ 156.0, 153.5, 138.7, 136.2, 133.2, 132.9, 131.9, 128.8, 119.9, 112.8, 112.2, 102.9, 64.9, 61.0, 56.3, 14.5. HRMS (ESI/Q-TOF): *m/z* [M+H]^+^ calcd. for C_19_H_21_BrN_3_O_4_^+^ [M+H]^+^: 434.0710, found: 434.0692.
**5-(3-chloro-4-methoxyphenyl)-1-(3,4,5-trimethoxyphenyl)-1H-1,2,3-triazole (3n):** Starting from **2f** (83 mg, 0.5 mmol) and following general procedure D, compound **3n** was purified by chromatography, eluting with *n*-hexane-EtOAc (1:1). White solid (0.10 g, 0.27 mmol), yield 54%, mp 107–109 °C. ^1^1H NMR (300 MHz, CDCl_3_) δ 7.83 (s, 1H), 7.36 (d, *J* = 2.2 Hz, 1H), 7.07 (dd, *J* = 8.6, 2.2 Hz, 1H), 6.89 (d, *J* = 8.6 Hz, 1H), 6.58 (s, 2H), 3.92 (s, 3H), 3.89 (s, 3H), 3.75 (s, 6H). ^13^C NMR (126 MHz, CDCl_3_) δ 155.9, 153.7, 138.9, 136.5, 133.0, 132.0, 130.4, 128.3, 123.2, 119.8, 112.2, 103.1, 61.2, 56.5, 56.4. HRMS (ESI/Q-TOF): *m/z* [M+H]^+^ calcd. for C_18_H_18_ClN_3_O_4_ [M+H]^+^: 376.1059, found: 376.1047.
**5-(3,4-dimethoxyphenyl)-1-(3,4,5-trimethoxyphenyl)-1H-1,2,3-triazoletriazole (3o):** Starting from **2a** (81 mg, 0.5 mmol) and following general procedure D, compound **3o** was purified by chromatography, eluting with *n*-hexane-EtOAc (2:1). White solid (0.11 g, 0.30 mmol), yield 59%, mp 119–121 °C. ^1^H NMR (400 MHz, CDCl_3_) δ 7.66 (s, 1H), 6.74 (s, 2H), 6.61 (s, 1H), 6.48 (s, 2H), 3.75 (s, 3H), 3.72 (s, 3H), 3.60 (s, 6H), 3.59 (s, 3H). ^13^C NMR (101 MHz, CDCl_3_) δ 153.2, 149.6, 148.7, 138.2, 137.4, 132.4, 132.0, 121.3, 118.8, 111.3, 111.2, 111.1, 102.8, 60.7, 56.0, 55.7, 55.6, 55.6. HRMS (ESI/Q-TOF): *m/z* [M+H]^+^ calcd. for C_19_H_22_N_3_O_5_^+^ [M+H]^+^: 372.1554, found: 372.1539.
**General procedure for the deprotection of TBDMS ethers for the synthesis of triazoles 3p-r.** Following the general procedure of the 1,3-dipolar Huisgen cycloaddition described before, under N_2_ atmosphere, tetra-*n*-butyl ammonium fluoride (TBAF, 1 M in THF, 1.5 equiv.) was added dropwise to a cooled (0 °C) solution of the corresponding TBDMS-ether in THF (0.05 M). The reaction mixture was stirred at the same temperature for 2 h and then treated with water. The mixture was extracted 3 times with ethyl acetate. The organic phase was dried over anhydrous Na_2_SO_4_, and the mixture was concentrated to dryness and purified by flash column chromatography on silica gel using mixtures of *n*-hexane and ethyl acetate as eluents.
**2-bromo-4-(1-(3,4,5-trimethoxyphenyl)-1H-1,2,3-triazol-5-yl)phenol (3p):** Starting from **2e** (99 mg, 0.5 mmol) and following the general procedure described before, compound **3p** was purified by chromatography, eluting with *n*-hexane-EtOAc (1:2). Orange solid (0.12 g, 0.29 mmol), yield 58%, mp >200 °C. ^1^H NMR (300 MHz, Acetone-d_6_) δ 8.01 (s, 1H), 7.92 (s, 1H), 7.55 (d, *J* = 2.1 Hz, 01H), 7.15 (dd, *J* = 8.4, 2.1 Hz, 1H), 7.02 (d, *J* = 8.4 Hz, 1H), 6.74 (s, 1H), 3.79 (s, 3H), 3.77 (s, 6H). ^13^C NMR (75 MHz, Acetone-d_6_) δ 156.3, 154.8, 137.7, 134.2, 133.4, 133.3, 130.1, 120.4, 117.5, 110.7, 104.7, 60.9, 56.8. HRMS (ESI/Q-TOF): *m/z* [M+H]^+^ calcd. for C_17_H_17_BrN_3_O_4_^+^ [M+H]^+^: 406.0397, found: 406.0379.
**4-(1-(3,4,5-trimethoxyphenyl)-1H-1,2,3-triazol-5-yl)benzene-1,2-diol (3q):** Starting from **2b** (74 mg, 0.5 mmol) and following the general procedure described before, compound **3q** was purified by chromatography, eluting with *n*-hexane-EtOAc (1:3). Yellow solid (0.13 g, 0.35 mmol), yield 71%, mp > 200 °C. ^1^H NMR (300 MHz, CD_3_OD) δ 7.85 (s, 1H), 6.80 (d, *J* = 7.8 Hz, 1H), 6.72–6.68 (m, 4 H), 3.82 (s, 3H), 3.75 (s, 6H). ^13^C NMR (75 MHz, CD_3_OD) δ 155.0, 148.1, 146.8, 140.1, 139.9, 133.6, 133.2, 121.9, 118.9, 116.7, 116.6, 104.4, 61.2, 56.8. HRMS (ESI/Q-TOF): *m/z* [M+K]^+^ calcd. for C_17_H_18_KN_3_O_5_^+^ [M+K]^+^: 382.0800, found: 382.0763.
**2-methoxy-5-(1-(3,4,5-trimethoxyphenyl)-1H-1,2,3-triazol-5-yl)phenol (3r):** Starting from **2c** (0.18 g, 0.5 mmol) and following the general procedure described before, compound **3r** was purified by chromatography, eluting with *n*-hexane-EtOAc (1:1). White solid (0.2 g, 0.35 mmol), yield 69%. NMR spectra match with previously published [15].

### 4.2. Biological Assays

**Cell culture.** Cell culture media were purchased from Gibco (Grand Island, NY, USA). Fetal bovine serum (FBS) was a product of Harlan-Seralab (Belton, UK). Supplements and other chemicals not listed in this section were obtained from Sigma Chemical Co. (St. Louis, MO, USA). Plastics for cell culture were supplied by Thermo Scientific BioLite. All tested compounds were dissolved in DMSO at a concentration of 20 mM and stored at –20 °C until use. HT-29, A-549, and HEK-293 cell lines were maintained in a Dulbecco’s modified Eagle’s medium (DMEM) containing glucose (1 g/L), glutamine (2 mM), penicillin (50 μg/mL), streptomycin (50 μg/mL), and amphotericin B (1.25 μg/mL), supplemented with 10% FBS.
**Cytotoxicity assays.** 5 × 10^3^ cells per well were incubated in 96-well plates with serial dilutions of the tested compounds in a total volume of 100 μL of their growth media. 3-(4,5-dimethylthiazol-2-yl)-2,5-diphenyltetrazolium bromide (MTT; Sigma Chemical Co.) dye reduction assay in 96-well microplates was used. Then, 10 μL of MTT (5 mg/mL in phosphate-buffered saline, PBS) was added to each well after 2 days of incubation (37 °C, 5% CO_2_ in a humid atmosphere). The plate was incubated for a further 3 h (37 °C). After that, the supernatant was discarded and 100 μL of DMSO were added in order to dissolve formazan crystals. The absorbance was read at 550 nm by spectro-photometry. For all concentrations of compound, cell viability was expressed as the percentage of the ratio between the mean absorbance of treated cells and the mean absorbance of untreated cells. Three independent experiments were performed, and the IC_50_ values (i.e., concentration half inhibiting cell proliferation) were graphically determined using GraphPad Prism 9 software.

## Figures and Tables

**Table 1:** Synthesis of aromatic alkynes **2** *^a^*.

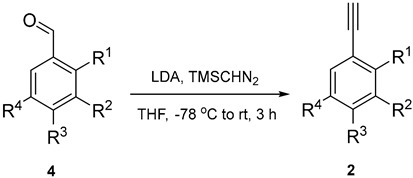						
Entry	4	R^1^	R^2^	R^3^	R^4^	2 (Yield %) *^b^*
1	**4a**	H	OMe	OMe	H	**2a** (50)
2	**4b**	H	OTBS	OTBS	H	**2b** (44)
3	**4c**	H	OTBS	OMe	H	**2c** (60)
4	**4d**	H	Br	OEt	H	**2d** (58)
5	**4e**	H	Br	OTBS	H	**2e** (36)
6	**4f**	H	Cl	OMe	H	**2f** (68)
7	**4g**	H	H	OEt	H	**2g** (52)
8	**4h**	Br	H	OMe	H	**2h** (60)
9	**4i**	Br	H	H	OMe	**2i** (42)

*^a^* General conditions: reactions were performed with **4** (1 mmol), LDA (1.9 equiv.), and trimethylsilyldiazomethane (1.25 equiv.) in THF (0.45 M) at −78 °C for 1 h and 2 h more at room temperature. *^b^* Isolated yields of terminal alkynes **2** after flash column chromatography.

**Table 2:** Synthesis of 1,5-disubstituted triazoles **3** *^a^*.

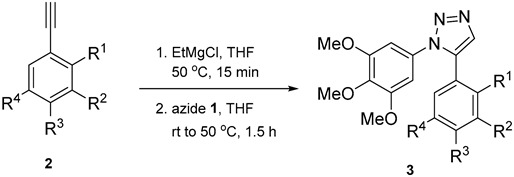						
Entry	3	R^1^	R^2^	R^3^	R^4^	Yield (%) *^b^*
1	**3a**	H	H	H	H	72
2	**3b**	Br	H	H	H	85
3	**3c**	NO_2_	H	H	H	59
4	**3d**	H	H	Me	H	60
5	**3e**	H	H	CF_3_	H	61
6	**3f**	H	H	F	H	51
7	**3g**	H	H	Cl	H	51
8	**3h**	H	H	Br	H	66
9	**3i**	H	H	OMe	H	60
10	**3j**	H	H	OEt	H	51
11	**3k**	Br	H	OMe	H	56
12	**3l**	Br	H	H	OMe	38
13	**3m**	H	Br	OEt	H	51
14	**3n**	H	Cl	OMe	H	54
15	**3o**	H	OMe	OMe	H	59
16	**3p**	H	Br	OH	H	58 *^c^*
17	**3q**	H	OH	OH	H	71 *^c^*
18	**3r**	H	OH	OMe	H	69 *^c^*

*^a^* General conditions: reactions were performed with **2** (0.5 mmol), EtMgCl (1.25 equiv.), and azide **1** (1 equiv.) in THF (0.45 M). *^b^* Isolated yields of triazoles **3** after flash column chromatography. *^c^* Global yield of two steps: formation of the triazole, followed by deprotection of OTBS groups with TBAF.

**Table 3:** Cytotoxicity (IC_50_ values in μM units) of *cis*-restricted triazol analogues of CA-4 (**3**) *^a^*.

Comp.	Structure	HT-29	A-549	HEK-293
**CA-4** *^b^*	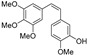	4.2 ± 0.5	0.428 ± 0.004	25 ± 3
**3a**	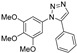	>100	>200	>200
**3b**	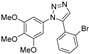	>100	>100	>200
**3c**	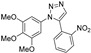	>100	>200	>200
**3d**	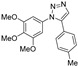	>100	>200	>200
**3e**	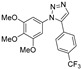	54 ± 3	>100	>200
**3f**	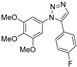	>200	>200	>200
**3g**	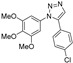	>100	>100	>200
**3h**	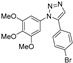	>100	>100	>200
**3i**	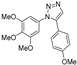	0.020 ± 0.009	0.022 ± 0.007	35 ± 9
**3j**	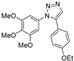	0.0021 ± 0.0009	5.8 ± 0.8	19 ± 4
**3k**	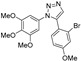	56 ± 4	33 ± 2	>100
**3l**	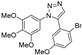	>100	>200	>200
**3m**	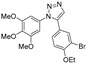	20 ± 5	59 ± 1	>200
**3n**	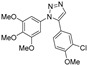	0.021 ± 0.006	0.0032 ± 0.0009	0.025 ± 0.008
**3o**	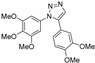	>100	>200	>200
**3p**	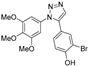	>200	>200	>200
**3q**	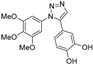	>200	>200	>200
**3r**	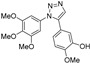	0.055 ± 0.007	0.0094 ± 0.0010	42 ± 5

*^a^* IC_50_ values are expressed as the compound concentration (μM) that causes 50% inhibition of cell growth, and are the average (±SD) of three different measurements after 48 h of incubation. *^b^* Data taken from ref. [30].

## Data Availability

The data are contained within the article or Supplementary Material.

## Supplementary Materials

The following supporting information can be downloaded at: https://www.mdpi.com/article/10.3390/molecules30020317/s1, with experimental data, spectroscopic characterizations, and graphical NMR spectra of new compounds. References [15,25,32,33,34,35,36,37,38,39,40,41,42] are cited in the Supplementary Materials.
