# Supplementary material for: Synthesis and Biological Evaluation of New cis-Restricted Triazole Analogues of Combretastatin A-4
Source: Molecules. 2025 Jan 15;30(2):317. doi: 10.3390/molecules30020317 (PMC11767582; doi:10.3390/molecules30020317)
Supplement: Supplementary file 1 [file molecules-30-00317-s001.zip › molecules-3414121-supplementary.pdf]

SUPPORTING INFORMATION

Synthesis and Biological Evaluation of New *cis*-Restricted Triazole Analogues of Combretastatin A-4

Lidia Prieto, Daniel Gaviña, Marcos Escolano, María Cánovas-Belchí, María Sánchez-Roselló, Carlos del Pozo, Eva Falomir, and Santiago Díaz-Oltra

Contents

General remarks S2

Synthesis of 3,4,5-trimethoxyaryl azide **1** S2

General procedure A. Synthesis of aldehydes **4a** and **4d** S3

General procedure B. Synthesis of aldehydes **4b**, **4c** and **4e** S3

General procedure C. Synthesis of alkynes **2** S4

References S5

NMR spectra of new compounds S7

HRMS spectra of new compounds S28

General remarks

NMR spectra were recorded on a Bruker 300 MHz, 400 MHz or 500 MHz spectrometer using deuterated chloroform ($CDCl_3$); deuterated methanol (CD_3OD) and deuterated acetone (acetone-d_6) as solvent. Chemical shifts (δ) are given in ppm relative to the residual solvent signals of non-deuterated chloroform 7.26 ppm for ^{1}H NMR and 77.16 ppm for ^{13}C NMR. Coupling constants (*J*) are given in Hertz (Hz). The letters m, s, d, t, q and bs stand for multiplet, singlet, doublet, triplet, quartet and broad singlet respectively.

Mass spectra were recorded on a VG AUTOESPEC (micromass) spectrometer, employing the electronic impact (EI) technic performed at 70 eV, whereas the acceleration speed of the ions beam Cs+ at the fast atom bombardment (FAB) spectra was 30,000 V. The listed values for each compound of the memory are expressed in units of m/z.

Melting points have been determined either with a Cambridge Instrusments or with a Büchi melting point P-450 apparatus.

Reactions and purifications were monitored with the aid of thin-layer chromatography (TLC) on 0.25 mm precoated Merck silica gel plates actives in UV light (Kiesegel 60 F254 on aluminium). Visualization was carried out with 254 nm UV light and employing potassium permanganate stain.

Flash column chromatography purifications was performed with the indicated solvents on silica gel 60 (particle size 0.040-0.063 mm).

Solvents employed were either distilled and dried under nitrogen atmosphere prior to use: THF and toluene were distilled from sodium, CH_2Cl_2 from calcium hydride; or anhydrous category solvents proceeding from commercial sources, being used without any previous purification.

Reagents employed were obtained of the best possible grade from commercial sources and were directly used. Air-sensitive reagents were employed under nitrogen atmosphere. Reactions were carried out under nitrogen atmosphere unless otherwise indicated.

Synthesis of 3,4,5-trimethoxyaryl azide 1

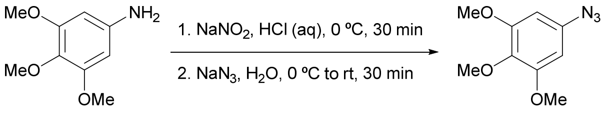

1 (>99%)

Under N_2 atmosphere, a solution of 3,4,5-trimetoxianiline (1 equiv., 13.53 mmol) in H_2O (33 mL) and $HCl_{conc.}$ (2 equiv., 2.36 mL) was stirred for 15 min at 0 °C. A solution of $NaNO_2$ (1.2 equiv., 16.24 mmol) in H_2O (16 mL) was added dropwise and stirred for 30 min at the same temperature. Then, a solution of sodium azide (1.2 equiv., 16.24 mmol) in H_2O (16 mL) was loaded dropwise. Upon completion of the addition, the mixture was allowed to come to room temperature and was kept stirring for 30 min. Ethyl acetate was added and the two phases were separated in a separatory funnel. The aqueous phase was extracted 2 more times with ethyl acetate. The organic phase was dried over anhydrous Na_2SO_4 and the mixture was concentrated to dryness to afford 3,4,5-trimethoxyaryl azide **2** as a brown solid in quantitative yield, and sufficiently pure to be used in the next reaction. NMR spectra match with previously published [1].

General procedure A. Synthesis of aldehydes 4a and 4d

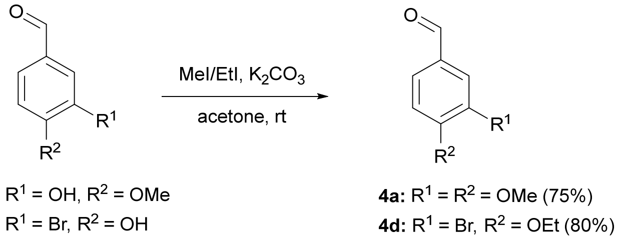

Under N_2 atmosphere, the corresponding aldehyde (1 equiv.) and potassium carbonate (2 equiv.) were dissolved in dimethylformamide (0.5 M). Then, 2 equiv. of MeI or EtI were added to form the corresponding alkyl aldehyde. The mixture was kept stirring for 3 hours at room temperature. Upon consumption of the substrate, CH_2Cl_2 and saturated aqueous NaCl were added and the two phases were separated in a separatory funnel. The aqueous phase was extracted 2 more times with CH_2Cl_2. The organic phase was dried over anhydrous Na_2SO_4 and the mixture was concentrated to dryness and purified by flash column chromatography on silica gel using mixtures of *n*-hexane and ethyl acetate as eluents.

3,4-dimethoxybenzaldehyde (4a): Starting from 3-hydroxy-4-methoxybenzaldehyde (1 g, 6.57 mmol) and following general procedure A, compound **4a** was obtained as a white solid (0.82 g, 4.95 mmol), yield 75%. NMR spectra match with previously published [2].

3-bromo-4-ethoxybenzaldehyde (4d): Starting from 3-bromo-4-hydroxybenzaldehyde (1 g, 4.97 mmol) and following general procedure A, compound **4d** was obtained as a white solid (0.91 g, 3.97 mmol), yield 80%. NMR spectra match with previously published [3].

General procedure B. Synthesis of aldehydes 4b, 4c and 4e

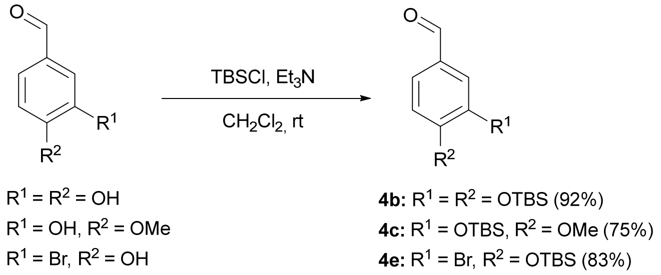

To a cooled (0 ºC) solution of the corresponding aldehyde (1 equiv.) in CH_2Cl_2 (0.33 M) triethylamine (1.5 equiv.) was added and stirred for 5 min at the same temperature. Then, a *tert*-butyldimethylsilyl chloride (TBDMSCl, 1.2 equiv.) was loaded. The mixture was allowed to come to room temperature and was kept stirring for 2 hours, upon consumption of the aldehyde. The reaction was worked up by addition of H_2O and the two phases were separated in a separatory funnel. The aqueous phase was extracted 2 more times with CH_2Cl_2. The organic phases were then combined and washed twice with a saturated aqueous NaCl solution and was dried over anhydrous Na_2SO_4. The mixture was concentrated to dryness and purified by flash column chromatography on silica gel using mixtures of *n*-hexane and ethyl acetate as eluents.

3,4-bis((*tert*-butyldimethylsilyl)oxy)benzaldehyde (4b): Starting from 3,4-dihydroxybenzaldehyde (1 g, 7.24 mmol) and following general procedure B, compound **4b** was obtained as a white solid (2.43 g, 6.63 mmol), yield 92%. NMR spectra match with previously published [4].

3-((*tert*-butyldimethylsilyl)oxy)-4-methoxybenzaldehyde (4c): Starting from 3-hydroxy-4-methoxybenzaldehyde (1 g, 6.57 mmol) and following general procedure B, compound **4c** was

obtained as a yellow oil (1.32 g, 4.95 mmol), yield 75%. NMR spectra match with previously published [5].

3-bromo-4-((*tert*-butyldimethylsilyl)oxy)benzaldehyde (4e): Starting from 3-bromo-4-hydroxybenzaldehyde (1 g, 4.97 mmol) and following general procedure B, compound **4e** was obtained as a brown solid (1.30 g, 4.12 mmol), yield 83%. NMR spectra match with previously published [6].

General procedure C. Synthesis of alkynes 2

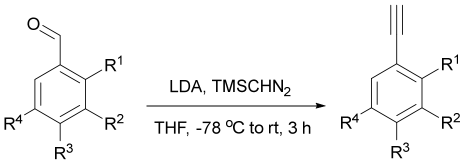

4 **2**

To a -78 °C cooled solution of LDA in THF (1.8 M, 1.9 equiv.), $TMSCHN_2$ 2.5 M in hexane (1.25 equiv.) was added under N_2 atmosphere and stirred for 1 hour at the same temperature. Then, a solution of the corresponding aldehyde **4** in THF (0.45 M, 1 equiv.) was loaded dropwise. Upon completion of the addition, the mixture was stirred for 1 hour at -78 °C and then, allowed to come to room temperature while stirring for 2 hours. Saturated aqueous NaCl was added, and the mixture was extracted 3 times with AcOEt. The organic phase was dried over anhydrous Na_2SO_4 and the mixture was concentrated to dryness and purified by flash column chromatography on silica gel using mixtures of *n*-hexane and ethyl acetate as eluents.

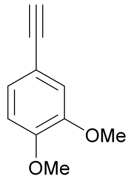

2a

4-ethynyl-1,2-dimethoxybenzene (2a): Starting from **4a** (0.17 g, 1 mmol) and following general procedure C, compound **2a** was obtained as colorless crystals (81 mg, 0.5 mmol), yield 50%. NMR spectra match with previously published [7].

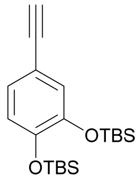

2b

((4-ethynyl-1,2-phenylene)bis(oxy))bis(*tert*-butyldimethylsilane) (2b): Starting from **4b** (0.36 g, 1 mmol) and following general procedure C, compound **2b** was obtained as a yellow oil (0.16 g, 0.44 mmol), yield 44%. NMR spectra match with previously published [8].

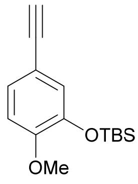

2c

***tert*-butyl(5-ethynyl-2-methoxyphenoxy)dimethylsilane (2c):** Starting from **4c** (0.26 g, 1 mmol) and following general procedure C, compound **2c** was obtained as a yellow oil (0.16 g, 0.6 mmol), yield 60%. NMR spectra match with previously published [9].

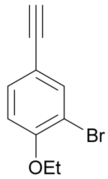

2d

2-bromo-1-ethoxy-4-ethynylbenzene (2d): Starting from **4d** (0.23 g, 1 mmol) and following general procedure C, compound **2d** was purified by chromatography, eluting with *n*-hexane-EtOAc (20:1). Orange solid (0.13 g, 0.58 mmol), yield 58%, mp 96-98 °C. ^{1}H NMR (300 MHz, $CDCl_3$) δ 7.68 (d, *J* = 2.0 Hz, 1H), 7.38 (dd, *J* = 8.5, 2.0 Hz, 1H), 6.80 (d, *J* = 8.5 Hz, 1H), 4.11 (q, *J* = 7.0 Hz, 2H), 3.02 (s, 1H), 1.47 (t, *J* = 7.0 Hz, 3H).^{13}C NMR (75 MHz, $CDCl_3$) δ 156.0, 137.0, 132.4, 115.5, 112.6, 111.8, 82.3, 77.1, 65.0, 14.7. HRMS (ESI/Q-TOF): *m/z* $[M+H]^+$ calcd. for $C_{10}H_{10}BrO^+$ $[M+H]^+$: 224.9910, not found.

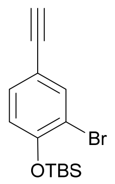

2e

(2-bromo-4-ethynylphenoxy)(*tert*-butyl)dimethylsilane (2e): Starting from **4e** (0.32 g, 1 mmol) and following general procedure C, compound **2e** was purified by chromatography, eluting with *n*-hexane. Brown liquid (0.11 g, 0.36 mmol), yield 36%. ^{1}H NMR (300 MHz, $CDCl_3$) δ 7.68 (d, *J* = 2.1 Hz, 1H), 7.30 (dd, *J* = 8.4, 2.1 Hz, 0H), 6.80 (d, *J* = 8.4 Hz, 1H), 3.02 (s, 1H), 1.04 (s, 9H), 0.26 (s, 6H). ^{13}C NMR (75 MHz, $CDCl_3$) δ 153.6, 137.2, 132.4, 116.3, 115.2, 82.3, 77.1, 25.8, 18.5, -4.09. HRMS (ESI/Q-TOF): *m/z* $[M+H]^+$ calcd. for $C_{14}H_{20}BrOSi^+$ $[M+H]^+$: 311.0461, not found.

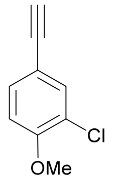

2f

2-chloro-4-ethynyl-1-methoxybenzene (2f): Starting from (0.17 g, 1 mmol) and following general procedure C, compound **2f** was obtained as a white solid (0.11 g, 0.68 mmol), yield 68%. NMR spectra match with previously published [10].

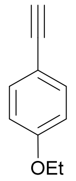

2g

1-ethoxy-4-ethynylbenzene (2g): Starting from (0.15 g, 1 mmol) and following general procedure C, compound **2g** was obtained as an orange solid (76 mg, 0.52 mmol), yield 52%. NMR spectra match with previously published [11].

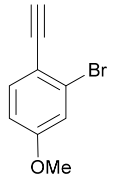

2h

2-bromo-1-ethynyl-4-methoxybenzene (2h): Starting from (0.22 g, 1 mmol) and following general procedure C, compound **2h** was obtained as a brown solid (0.13 g, 0.60 mmol), yield 60%. NMR spectra match with previously published [12].

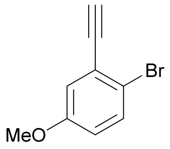

2i

1-bromo-2-ethynyl-4-methoxybenzene (2i): Starting from (0.22 g, 1 mmol) and following general procedure C, compound **2i** was obtained as a brown solid (89 mg, 0.42 mmol), yield 42%. NMR spectra match with previously published [13].

References

1. Hu, M.; Li, J.; Yao, S.Q. In situ "click" Assembly of Small Molecule Matrix Metalloprotease Inhibitors Containing Zinc-Chelating Groups. *Org. Lett.* **2008**, *10*, 5529–5531. https://doi.org/10.1021/ol802286g
2. Sun, N.; Zhang, X.; Jin, L.; Hu, B.; Shen, Z.; Hu, X. Recyclable copper-catalyzed ambient aerobic oxidation of primary alcohols to aldehydes in water using water-soluble PEG-functionalized pyridine triazole as ligand. *Cat. Commun.* **2017**, *101*, 5–9. https://doi.org/10.1016/j.catcom.2017.07.010
3. Kim, G.-R.; Kim, S.; Kim, Y.-O.; Han, X.; Nagel, J.; Kim, J.; Song, D. I.; Müller, C. E.; Jin, M. S.; Kim, Y.-C. Discovery of Triazolopyrimidine Derivatives as Selective P2X3 Receptor Antagonists Binding to an Unprecedented Allosteric Site as Evidenced by Cryo-Electron Microscopy. *J. Med. Chem.*, **2024**, *67*, 14443–14465. https://doi.org/10.1021/acs.jmedchem.4c01214
4. Yang, R.; Tavares, M. T.; Teixeira, S. F.; Azevedo, R. A.; Pietro, D. C.; Fernandes, T. B.; Ferreira, A. K.; Trossini, G. H. G.; Barbuto, J. A. M.; Parise-Filho, R. Toward chelerythrine optimization: Analogues designed by molecular simplification exhibit selective growth inhibition in non-small-cell lung cancer cells. *Biorg. Med. Chem.*, **2016**, *24*, 4600–4610. https://doi.org/10.1016/j.bmc.2016.07.065
5. Li, L.; Huang, X.; Huang, R.; Gou, S.; Wang, Z.; Wang, H. Pt(IV) prodrugs containing microtubule inhibitors displayed potent antitumor activity and ability to overcome cisplatin resistance. *Eur. J. Med. Chem.* **2018**, *156*, 666–679. https://doi.org/10.1016/j.ejmech.2018.07.016

6. Tello-Aburto, R.; Harned, A. M. Palladium-Catalyzed Reactions of Cyclohexadienones: Regioselective Cyclizations Triggered by Alkyne Acetoxylation. *Org. Lett.*, **2009**, *11*, 3998–4000. https://doi.org/10.1021/ol901642w
7. Rosiak, A.; Frey, W.; Christoffers, J. Synthesis of Tetrahydropyran-4-ones and Thiopyran-4-ones from Donor-Substituted α-Bromostyrene Derivatives. *Eur. J. Org. Chem.*, **2006**, 4044–4054. https://doi.org/10.1002/ejoc.200600372
8. Uchiyama, M.; Ozawa, H.; Takuma, K.; Matsumoto, Y.; Yonehara, M.; Hiroya, K.; Sakamoto, T. Regiocontrolled Intramolecular Cyclizations of Carboxylic Acids to Carbon−Carbon Triple Bonds Promoted by Acid or Base Catalyst. *Org. Lett.* **2006**, *8*, 5517–5520. https://doi.org/10.1021/ol062190+
9. Odlo, K.; Hentzen, J.; Dit Chabert, J.F.; Ducki, S.; Gani, O.A.; Sylte, I.; Skrede, M.; Flørenes, V.A.; Hansen, T.V. 1,5-Disubstituted 1,2,3-triazoles as *cis*-restricted analogues of combretastatin A-4: Synthesis, molecular modeling and evaluation as cytotoxic agents and inhibitors of tubulin. *Bioorg. Med. Chem.* **2008**, *16*, 4829–4838. https://doi.org/10.1016/j.bmc.2008.03.049
10. Odlo, K.; Fournier-Dit-Chabert, J.; Ducki, S.; Gani, O.A.; Sylte, I.; Hansen, T.V. 1,2,3-triazole analogs of combretastatin A-4 as potential microtubule-binding agents. *Bioorg. Med. Chem.* **2010**, *18*, 6874–6885. https://doi.org/10.1016/j.bmc.2010.07.032
11. Yao, W.; Li, R.; Jiang, H.; Han, D. An Additive-Free, Base-Catalyzed Protodesilylation of Organosilanes. *J. Org. Chem.* **2018**, *83*, 2250–2255. https://doi.org/10.1021/acs.joc.7b03139
12. Jin, H.; Liu, D.; Zhou, B.; Liu, Y. One-Pot Copper-Catalyzed Three-Component Reaction of Sulfonyl Azides, Alkynes, and Allylamines To Access 2,3-Dihydro-1H-imidazo [1,2-a]indoles. *Synthesis* **2020**, *52*, 1417–1424. https://doi.org/10.1055/s-0037-1610739
13. Madden, K. S.; Laroche, B.; David, S.; Batsanov, A. S.; Thompson, D.; Knowles, J. P.; Whiting, A. Approaches to Styrenyl Building Blocks for the Synthesis of Polyene Xanthomonadin and its Analogues. *Eur. J. Org. Chem.* **2018**, 5312–5322. https://doi.org/10.1002/ejoc.201800540

NMR spectra of new compounds

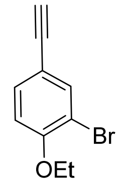

2d

^{1}H-NMR (300 MHz, $CDCl_3$)

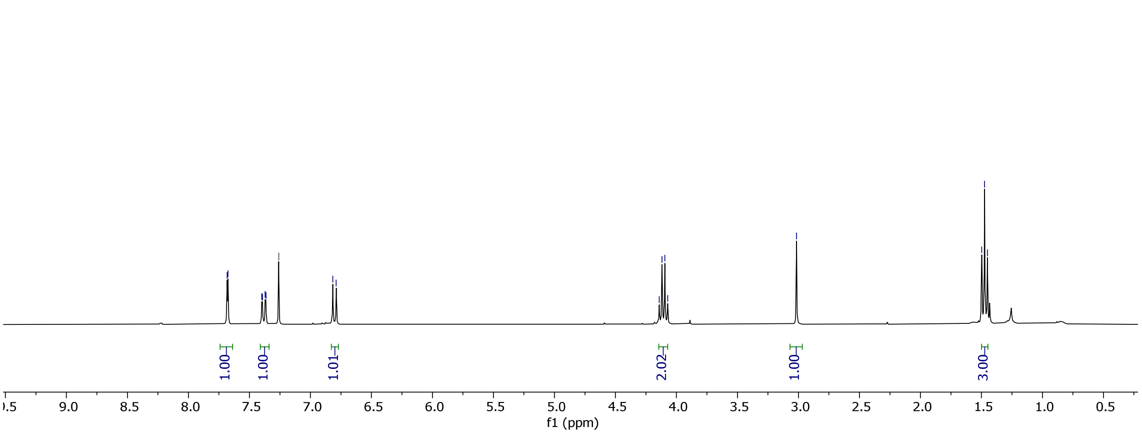

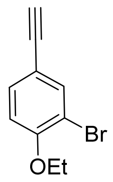

2d

^{13}C-NMR (75 MHz, $CDCl_3$)

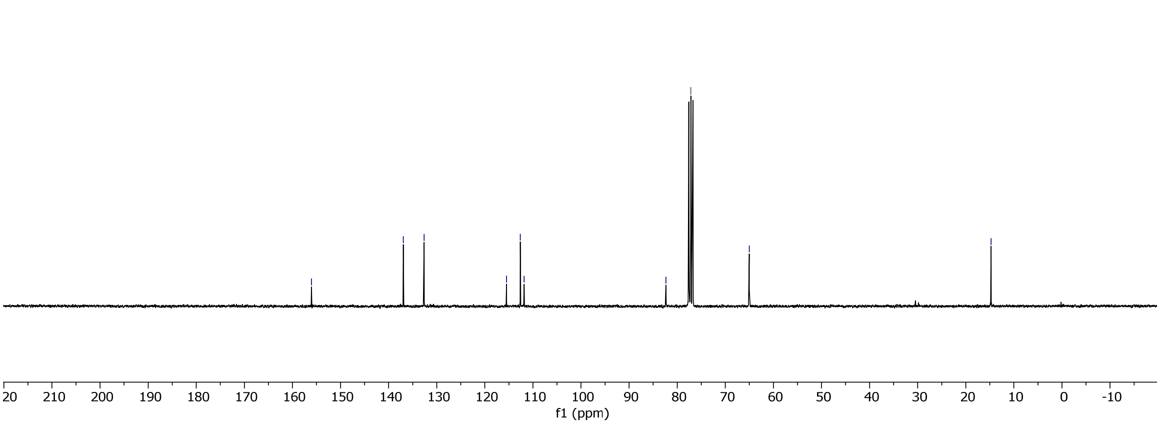

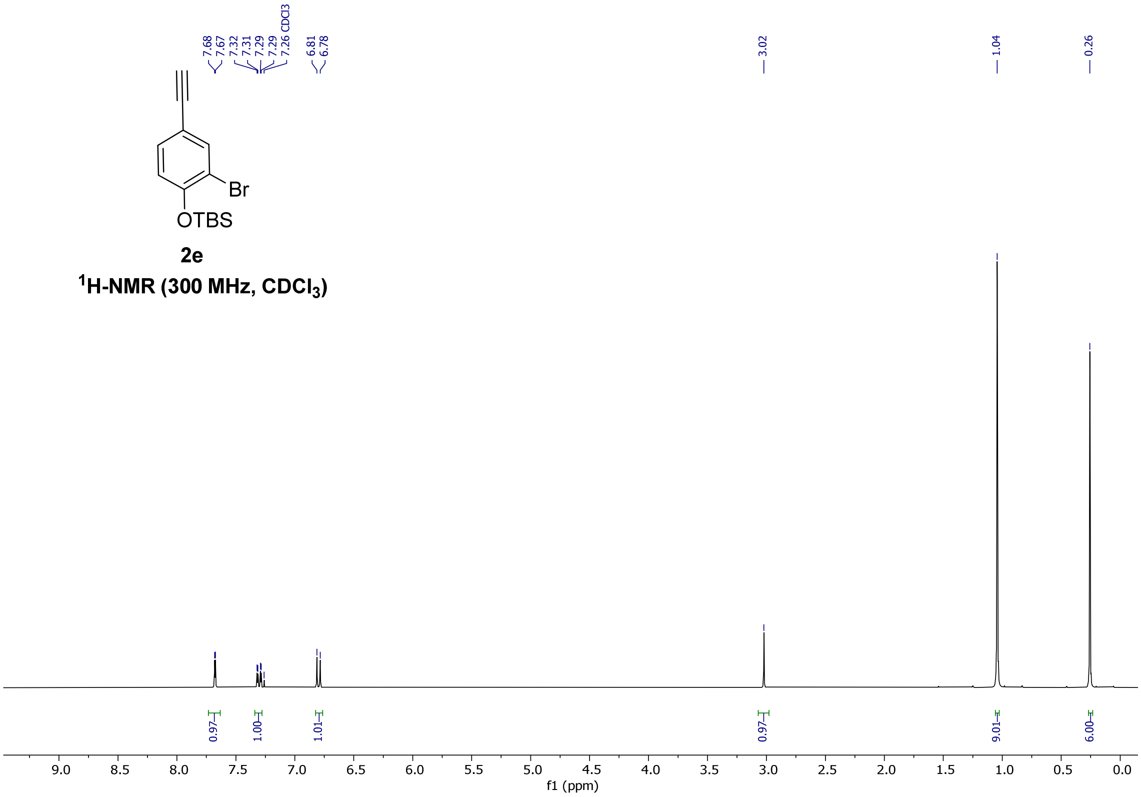

2e

^{1}H-NMR (300 MHz, $CDCl_3$)

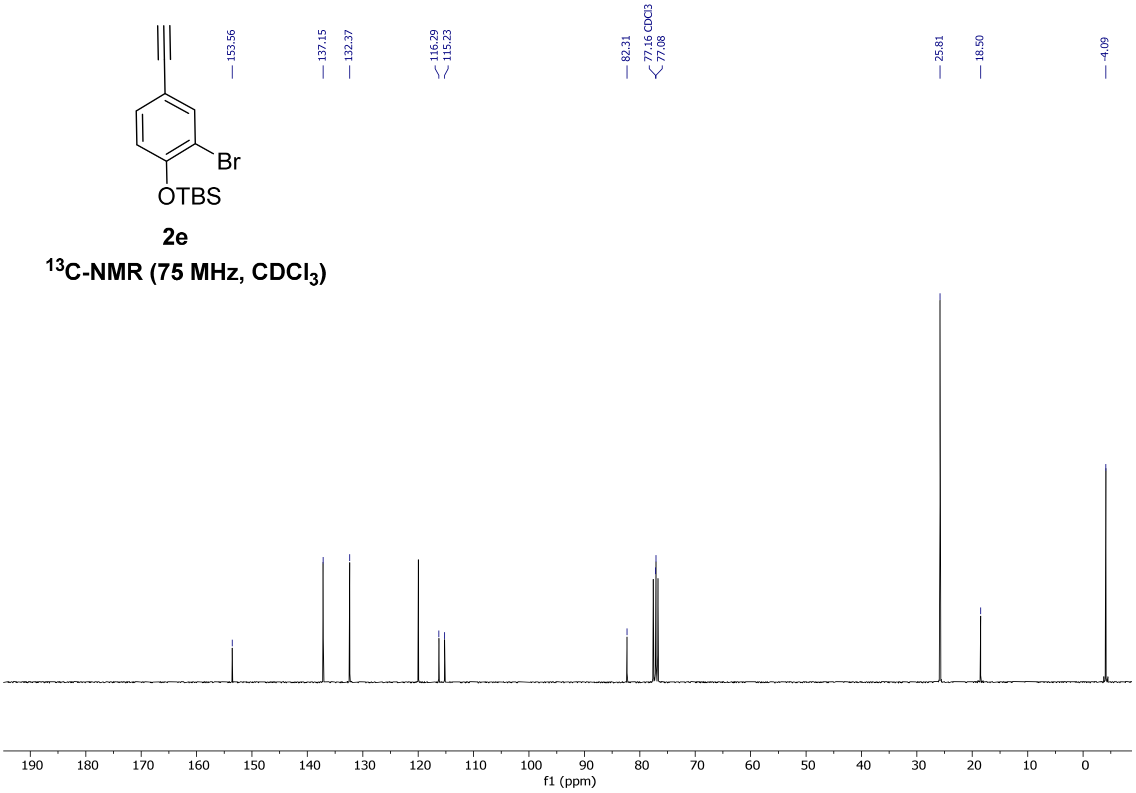

2e

^{13}C-NMR (75 MHz, $CDCl_3$)

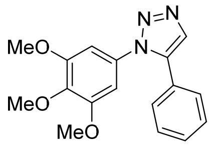

3a

^{1}H-NMR (300 MHz, $CDCl_3$)

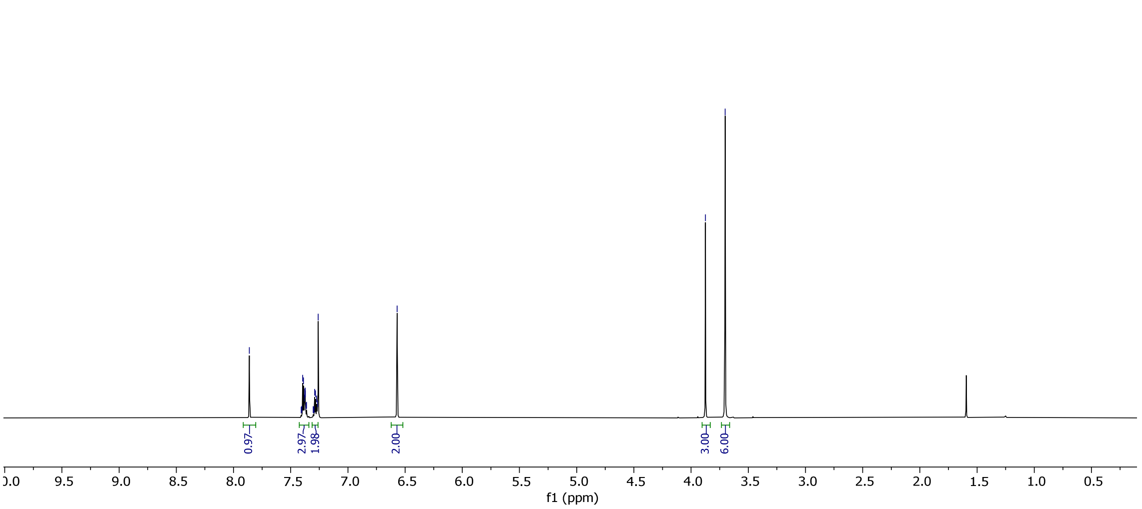

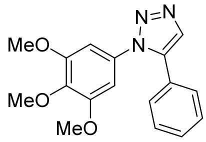

3a

^{13}C-NMR (75 MHz, $CDCl_3$)

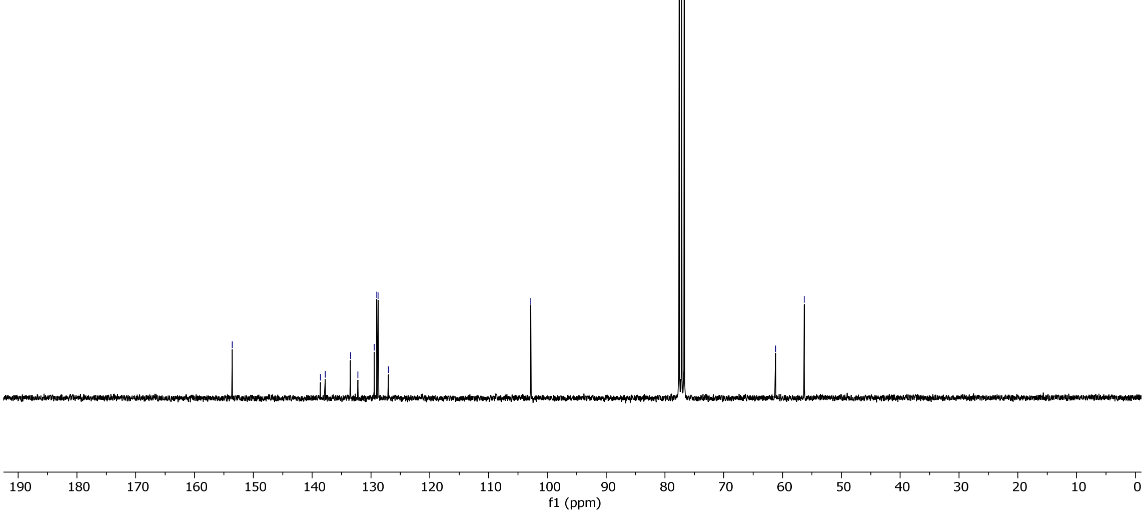

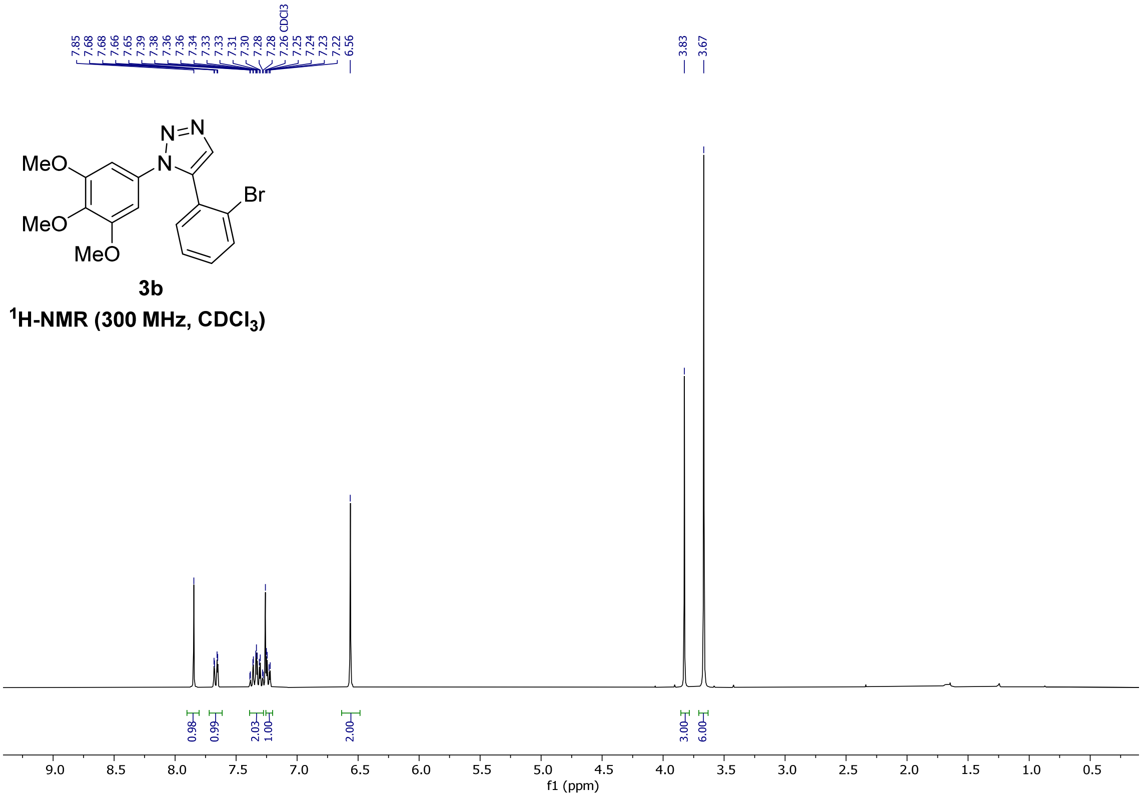

3b
^{1}H-NMR (300 MHz, $CDCl_3$)

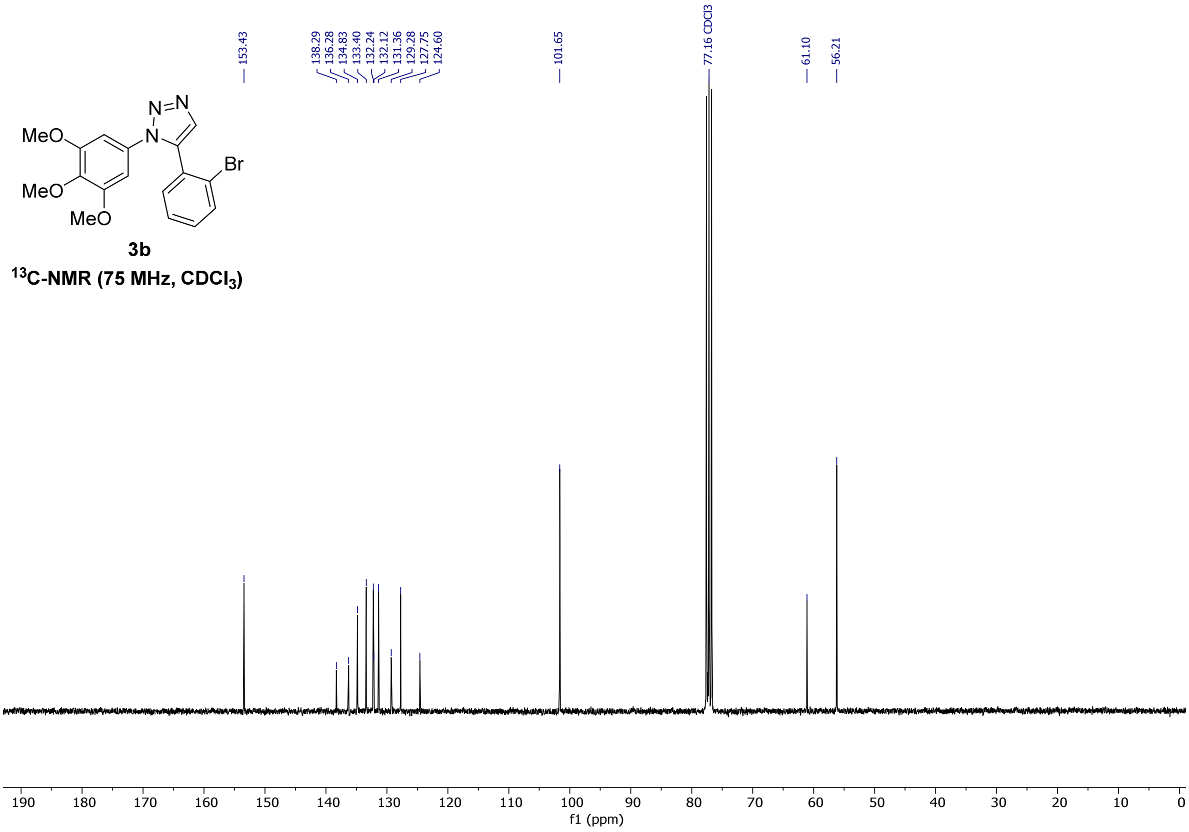

3b
^{13}C-NMR (75 MHz, $CDCl_3$)

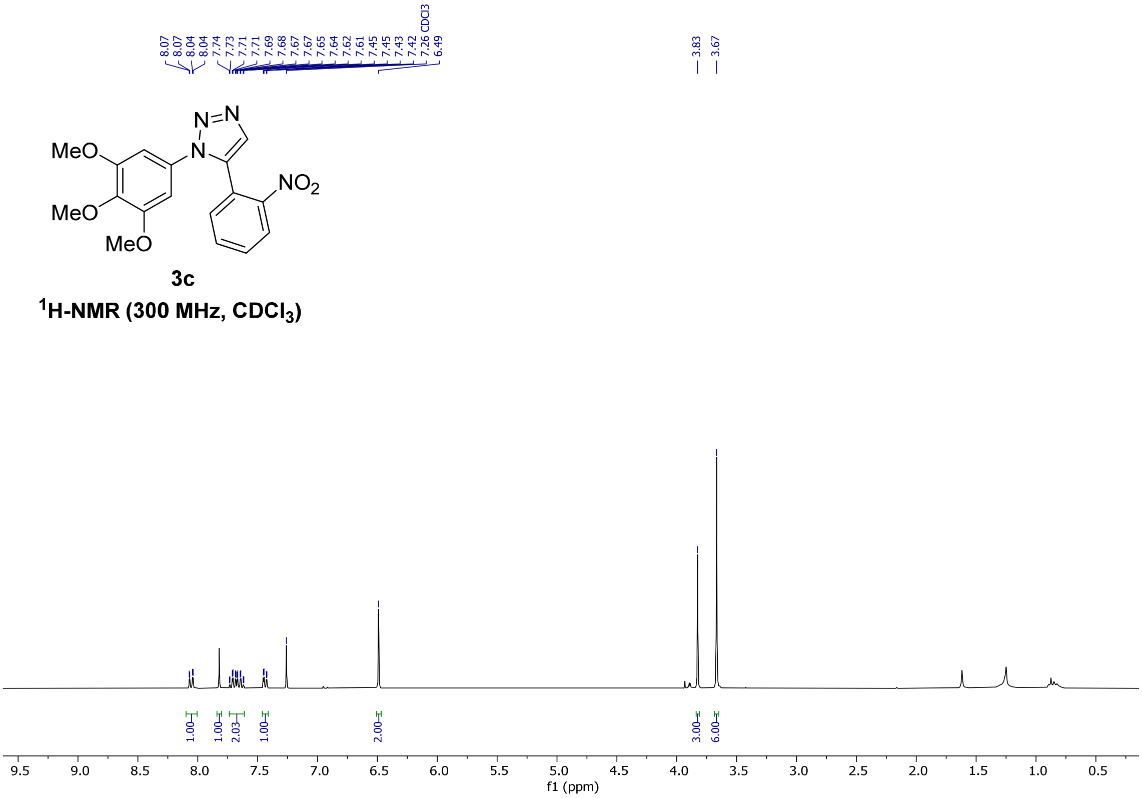

8.07
8.07
8.04
8.04
7.74
7.73
7.71
7.71
7.69
7.68
7.67
7.67
7.65
7.64
7.62
7.61
7.45
7.45
7.43
7.42
7.26 CDCl3
6.49
3.83
3.67
MeO
MeO
MeO
N
N
N
NO2
3c
1H-NMR (300 MHz, CDCl3)
1.00
1.00
2.03
1.00
2.00
3.00
6.00
9.5
9.0
8.5
8.0
7.5
7.0
6.5
6.0
5.5
5.0
4.5
4.0
3.5
3.0
2.5
2.0
1.5
1.0
0.5
f1 (ppm)

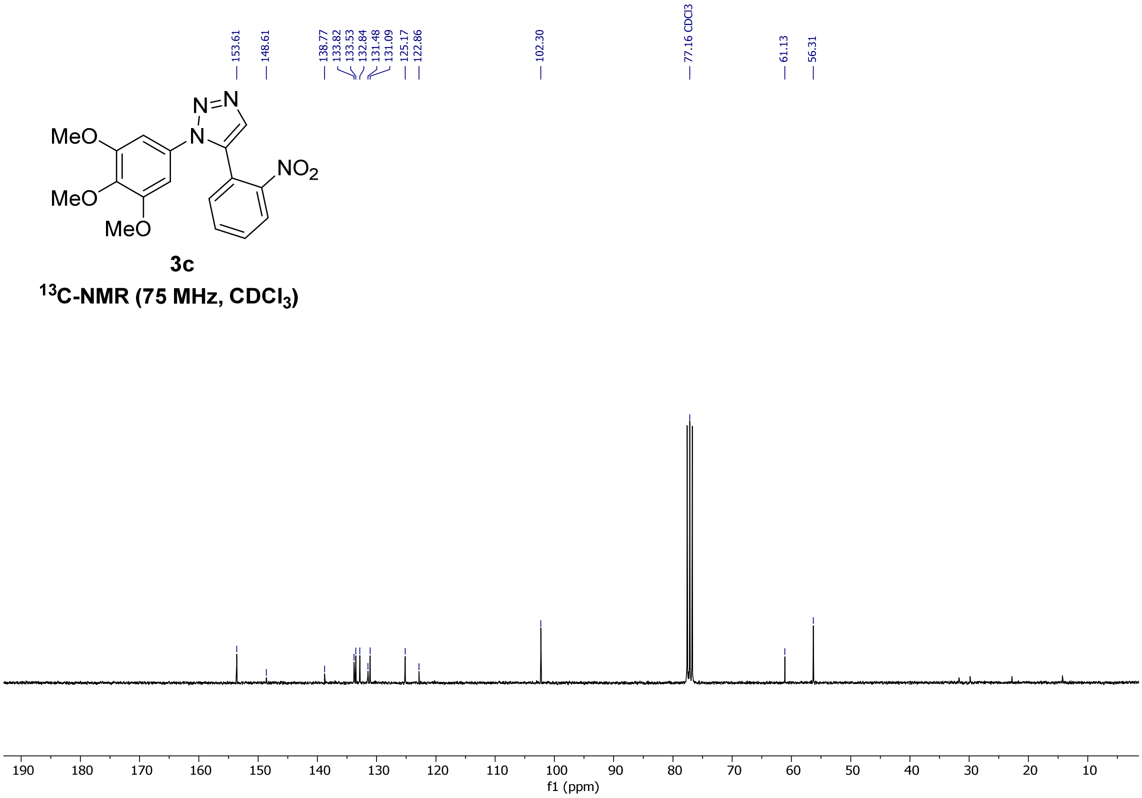

153.61
148.61
138.77
133.82
133.53
132.84
131.48
131.09
125.17
122.86
102.30
77.16 CDCl3
61.13
56.31
MeO
MeO
MeO
N
N
N
NO2
3c
13C-NMR (75 MHz, CDCl3)
190
180
170
160
150
140
130
120
110
100
90
80
70
60
50
40
30
20
10
f1 (ppm)

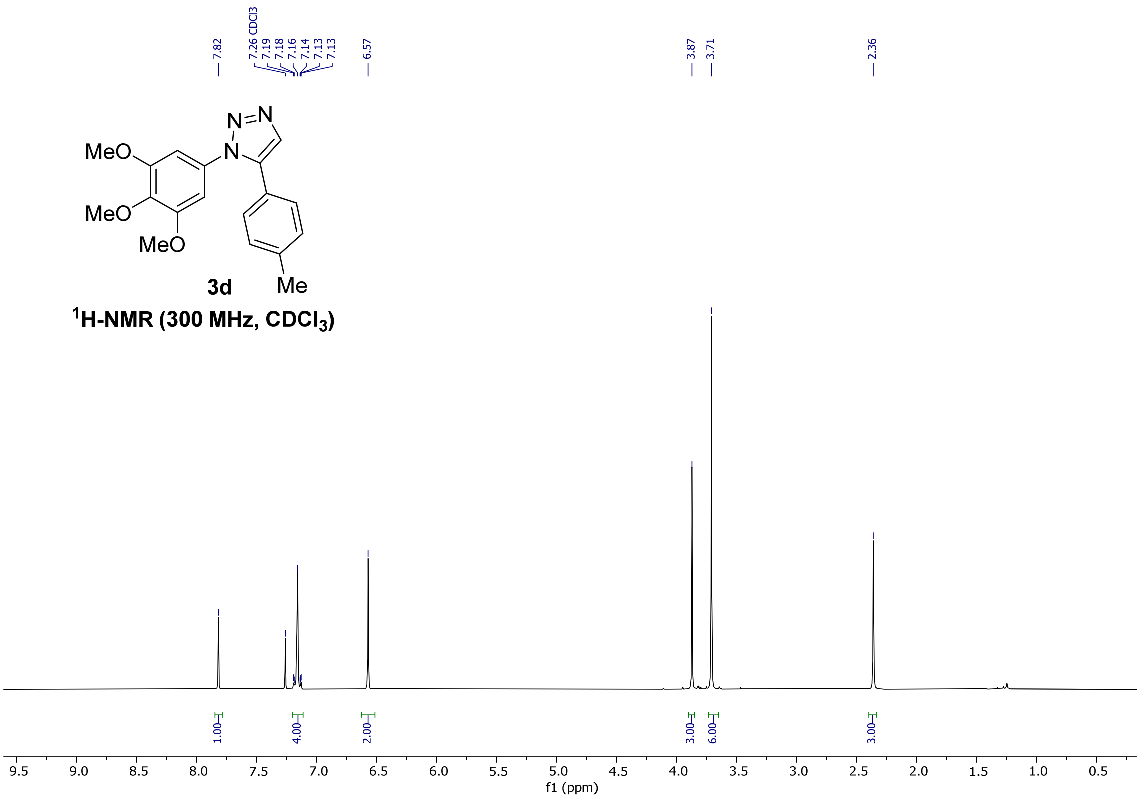

3d

^{1}H-NMR (300 MHz, $CDCl_3$)

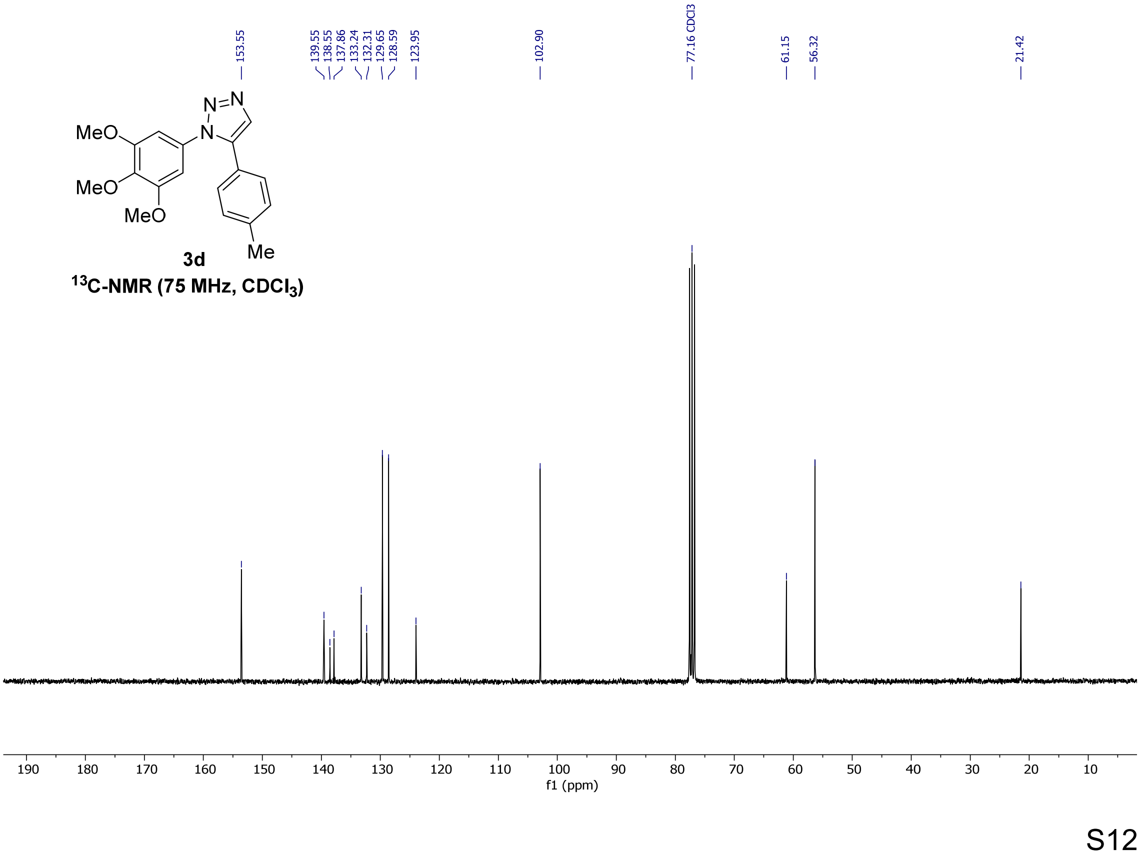

3d

^{13}C-NMR (75 MHz, $CDCl_3$)

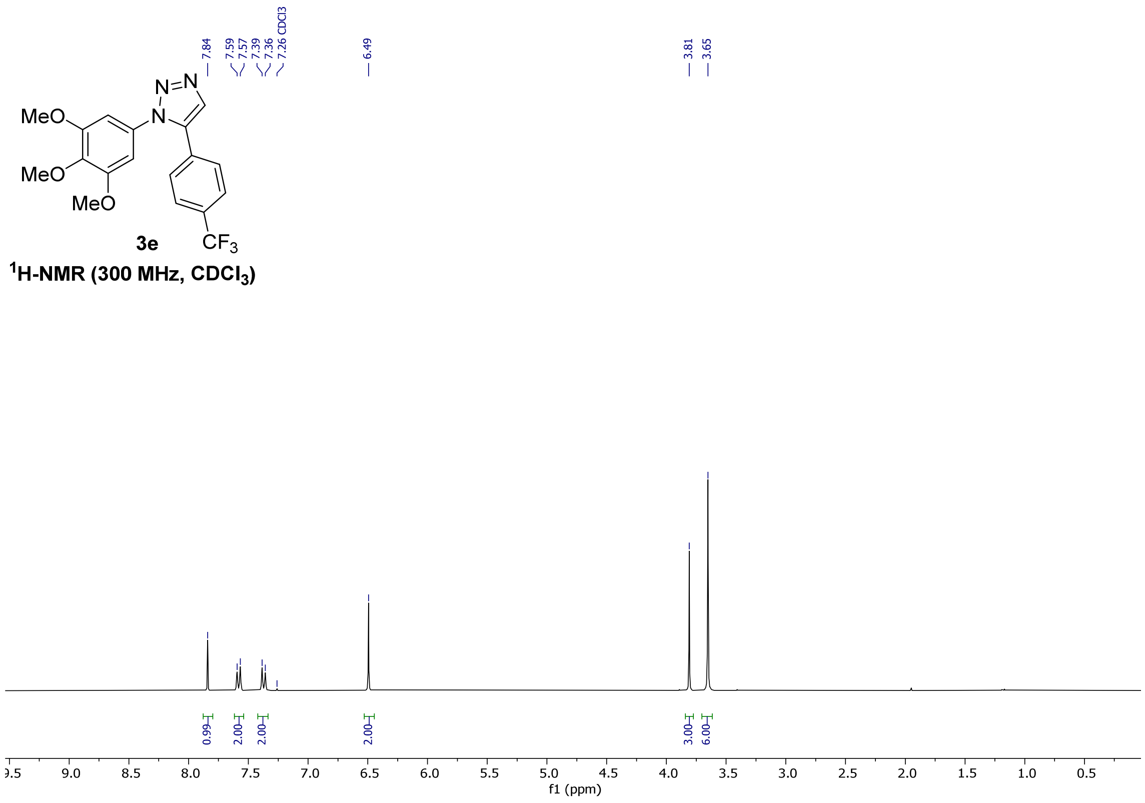
3e
^{1}H-NMR (300 MHz, $CDCl_3$)
f1 (ppm)

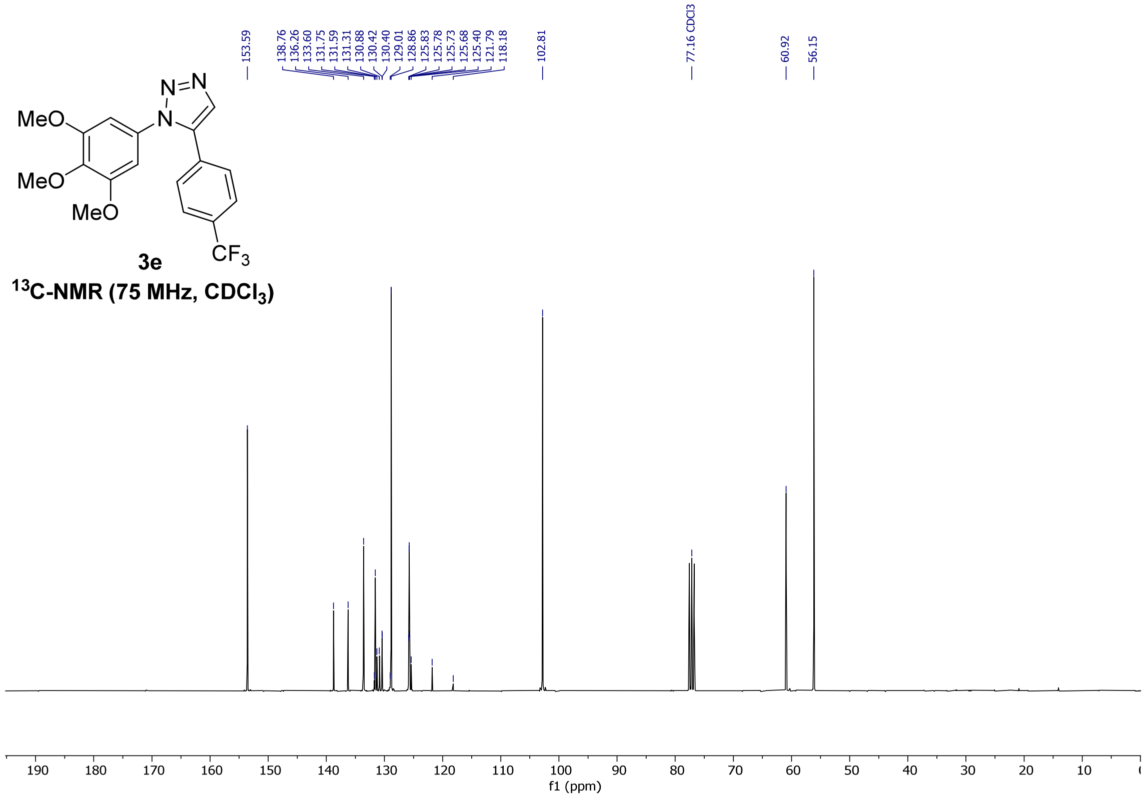
3e
^{13}C-NMR (75 MHz, $CDCl_3$)
f1 (ppm)

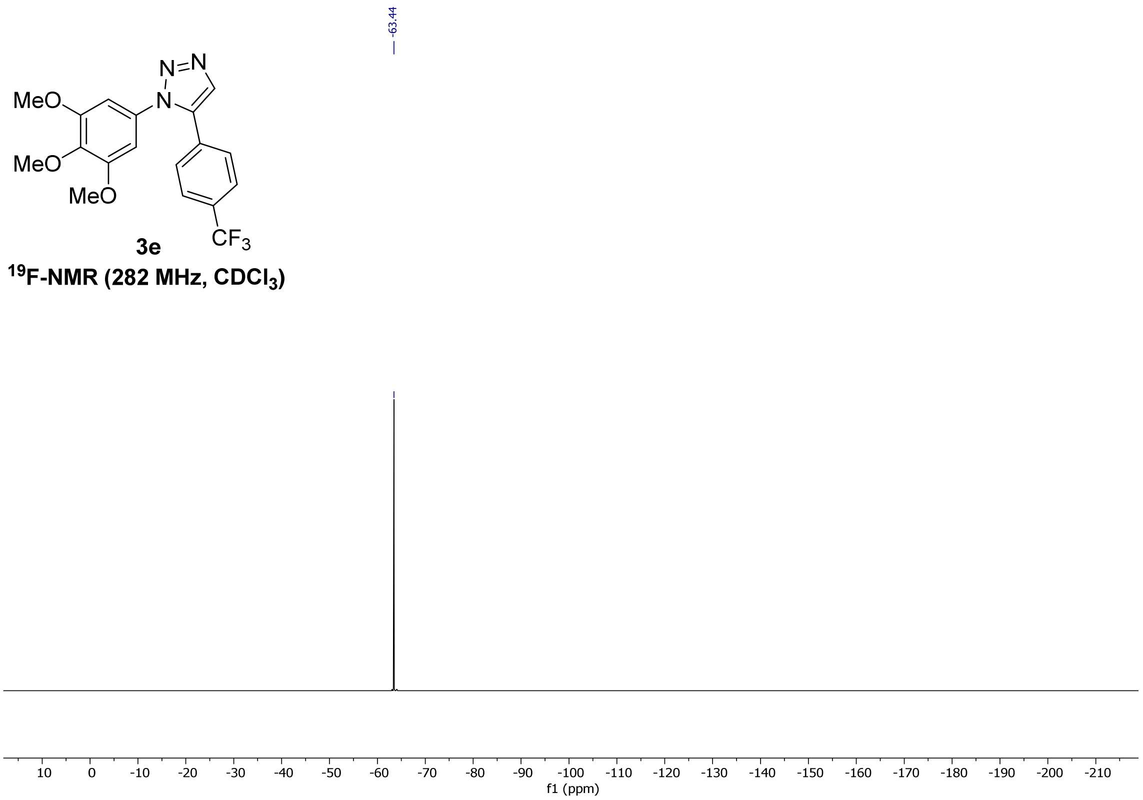

3e

^{19}F-NMR (282 MHz, $CDCl_3$)

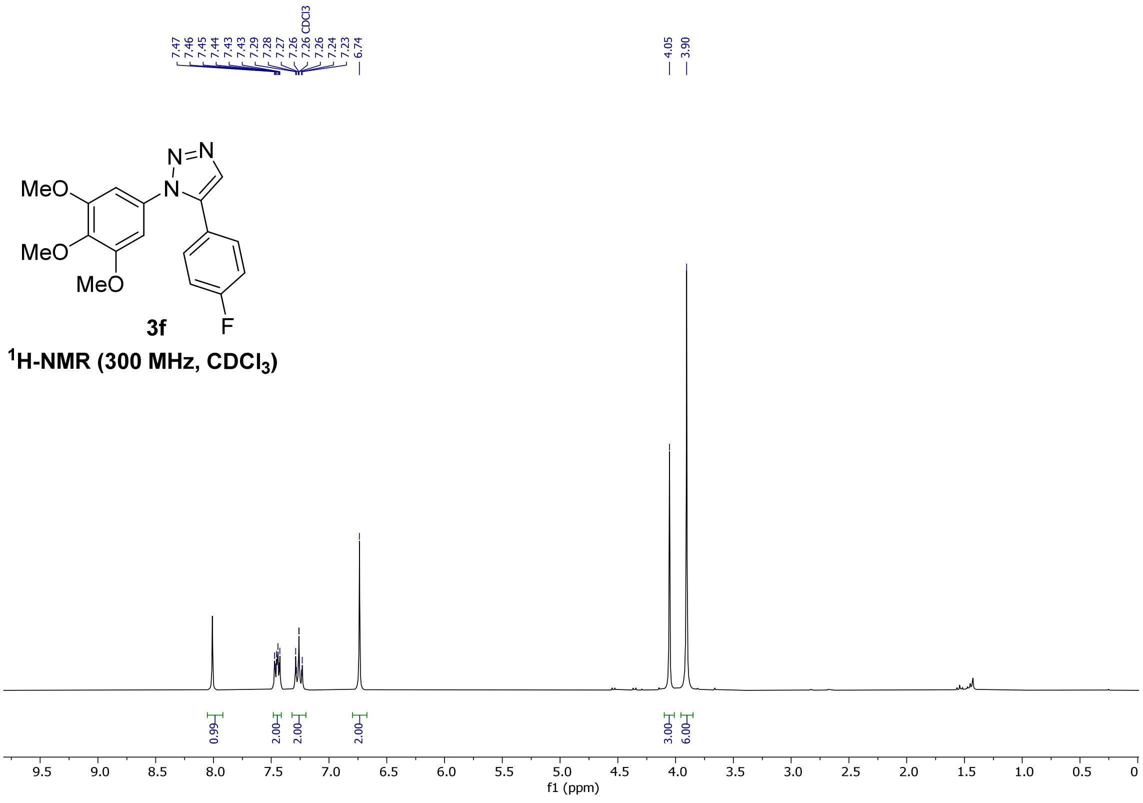

3f

^{1}H-NMR (300 MHz, $CDCl_3$)

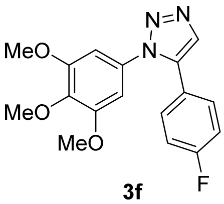

3f

^{13}C-NMR (75 MHz, $CDCl_3$)

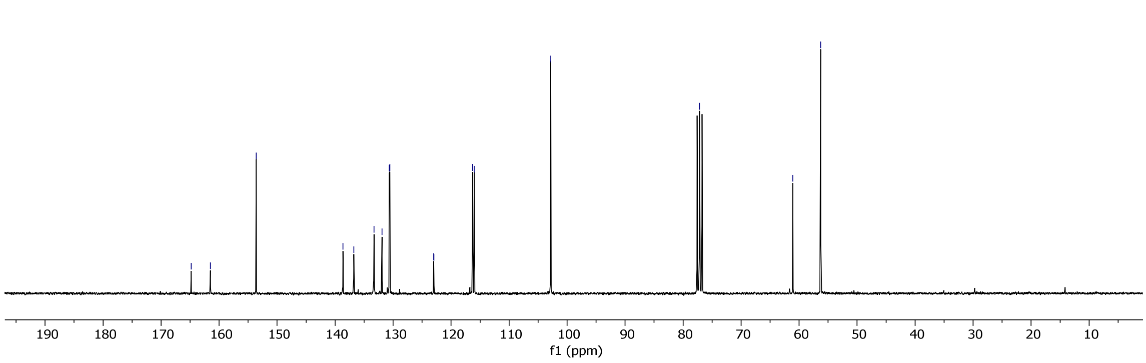

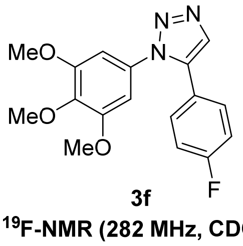

3f

^{19}F-NMR (282 MHz, $CDCl_3$)

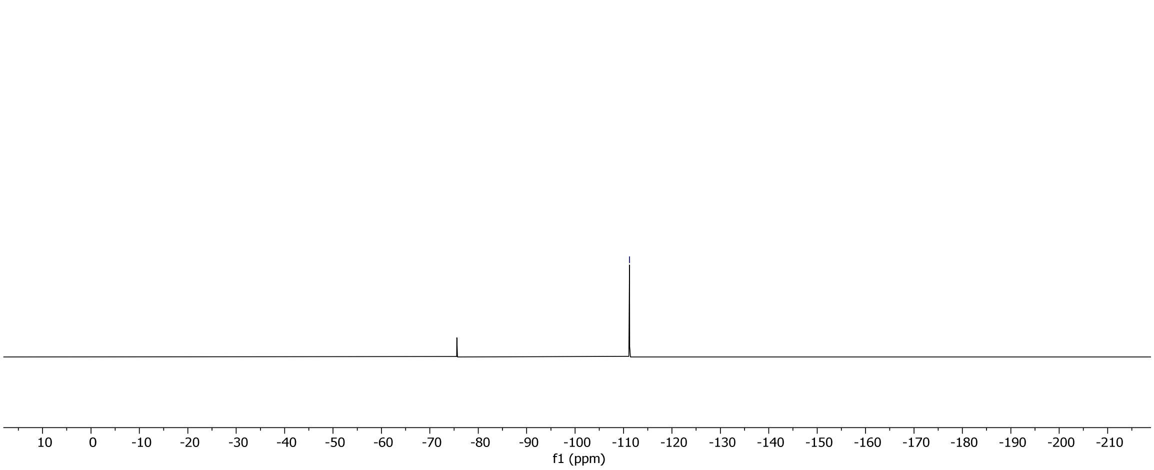

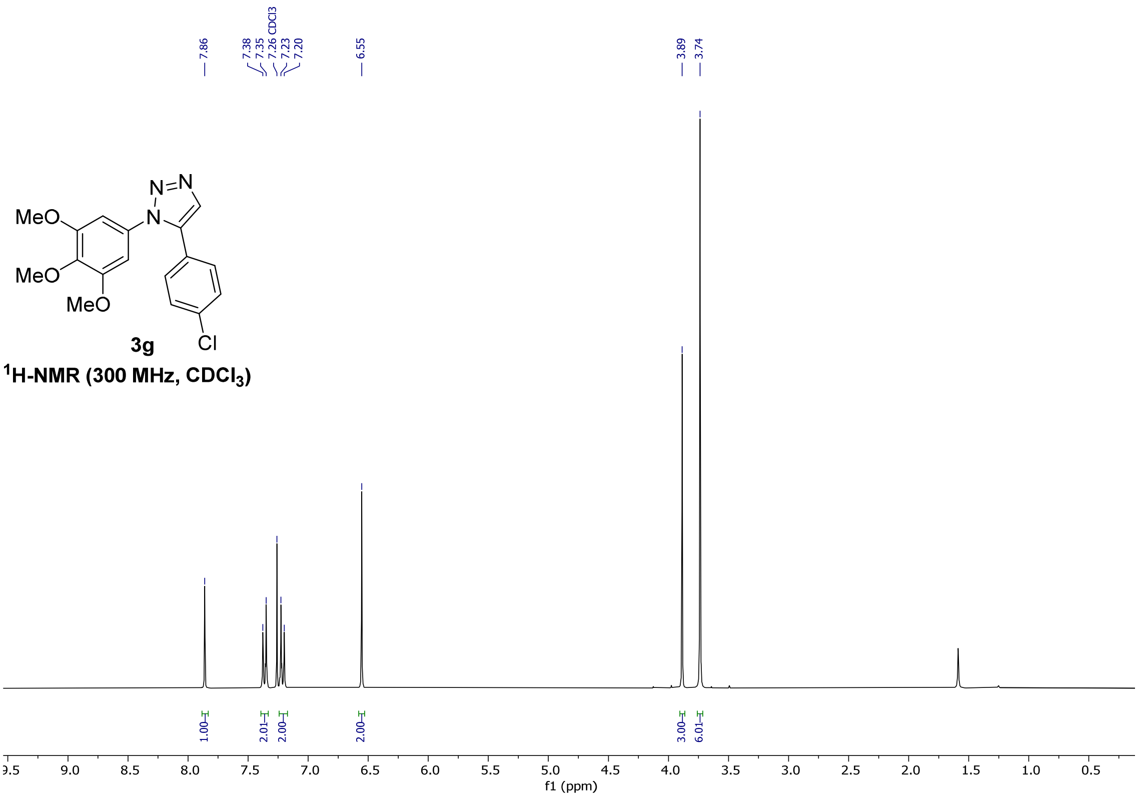

3g

^{1}H-NMR (300 MHz, $CDCl_3$)

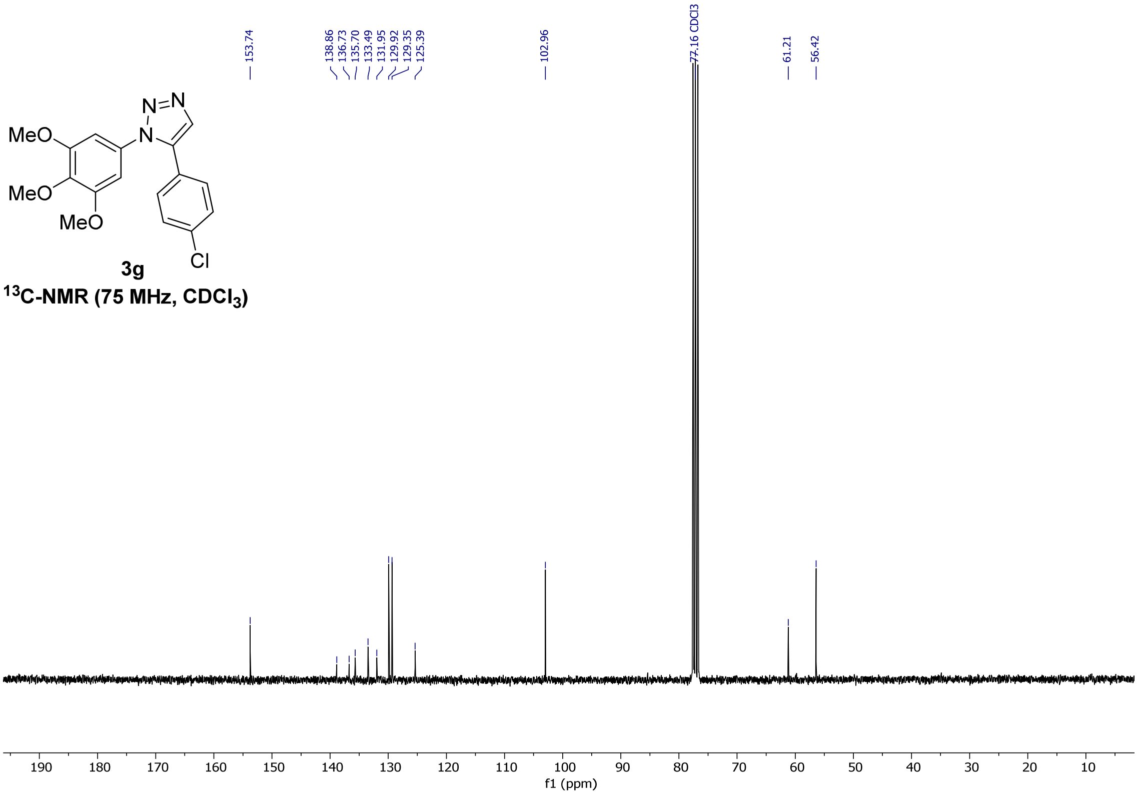

3g

^{13}C-NMR (75 MHz, $CDCl_3$)

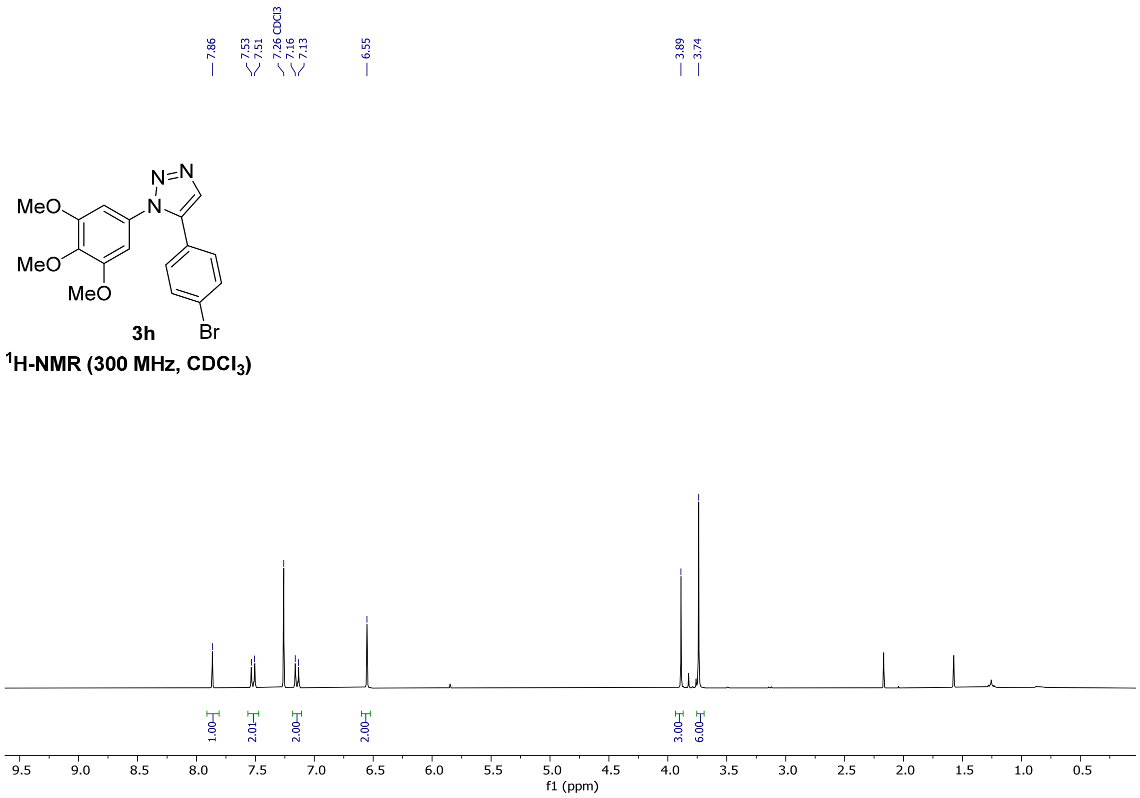

3h

^{1}H-NMR (300 MHz, CDCl$_3$)

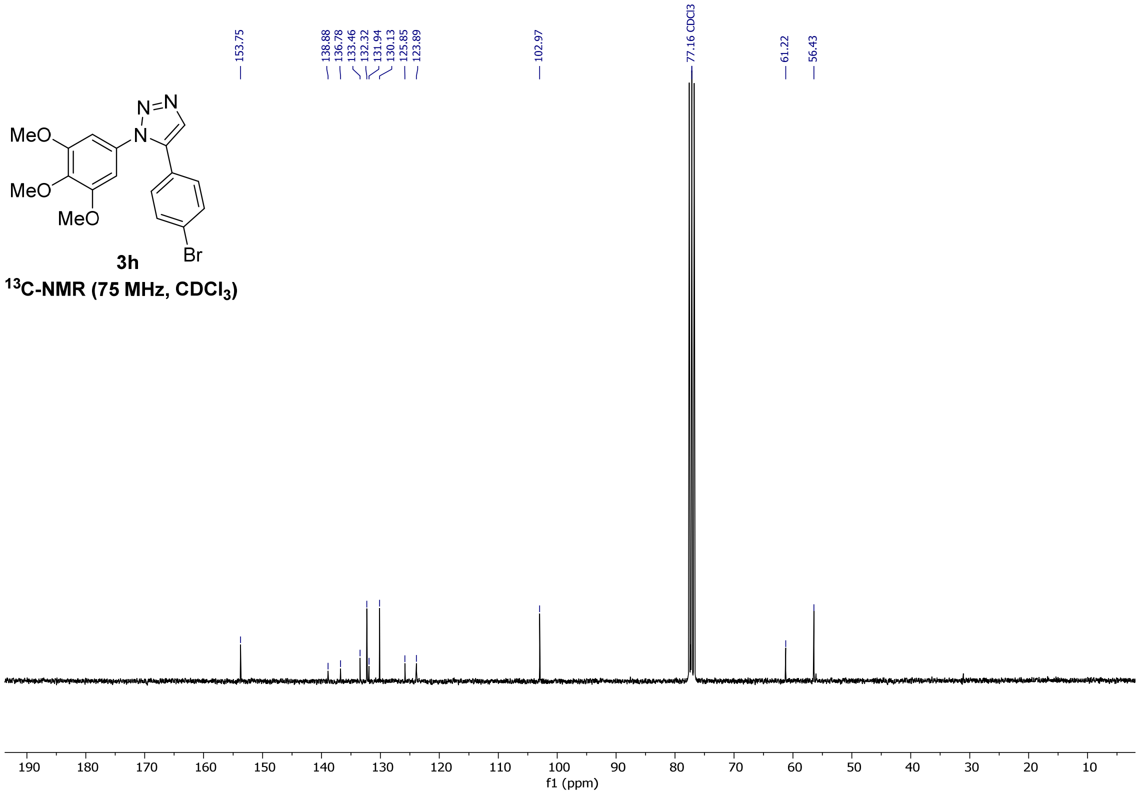

3h

^{13}C-NMR (75 MHz, CDCl$_3$)

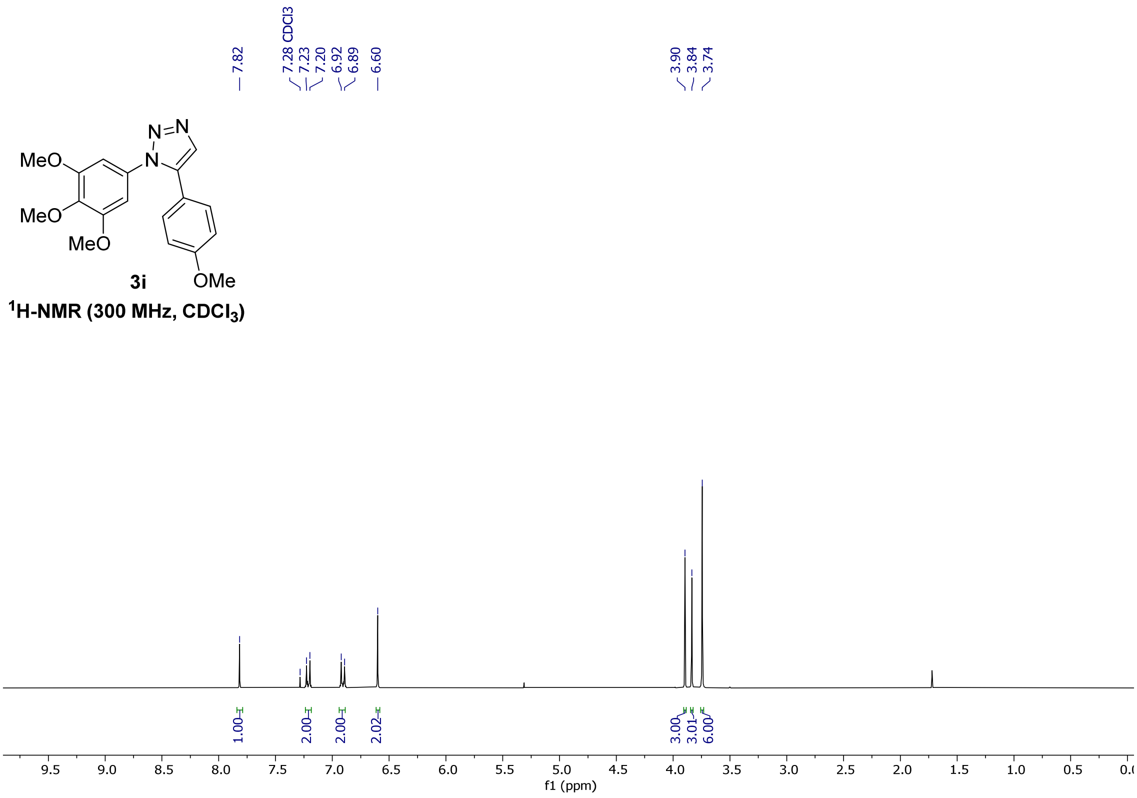
7.82
7.28 CDCl3
7.23
7.20
6.92
6.89
6.60
3.90
3.84
3.74
MeO
MeO
MeO
N
N
N
OMe
3i
1H-NMR (300 MHz, CDCl3)
1.00
2.00
2.00
2.02
3.00
3.01
6.00
9.5 9.0 8.5 8.0 7.5 7.0 6.5 6.0 5.5 5.0 4.5 4.0 3.5 3.0 2.5 2.0 1.5 1.0 0.5
f1 (ppm)

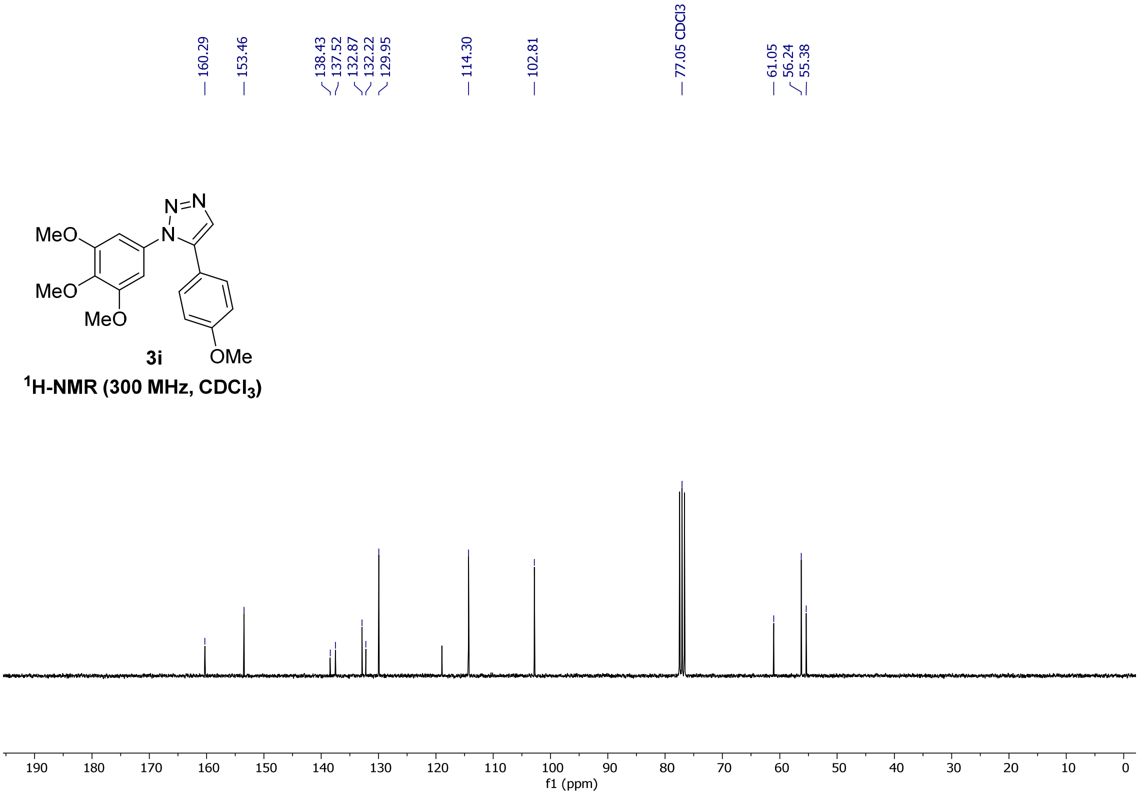
160.29
153.46
138.43
137.52
132.87
132.22
129.95
114.30
102.81
77.05 CDCl3
61.05
56.24
55.38
MeO
MeO
MeO
N
N
N
OMe
3i
1H-NMR (300 MHz, CDCl3)
190 180 170 160 150 140 130 120 110 100 90 80 70 60 50 40 30 20 10 0
f1 (ppm)

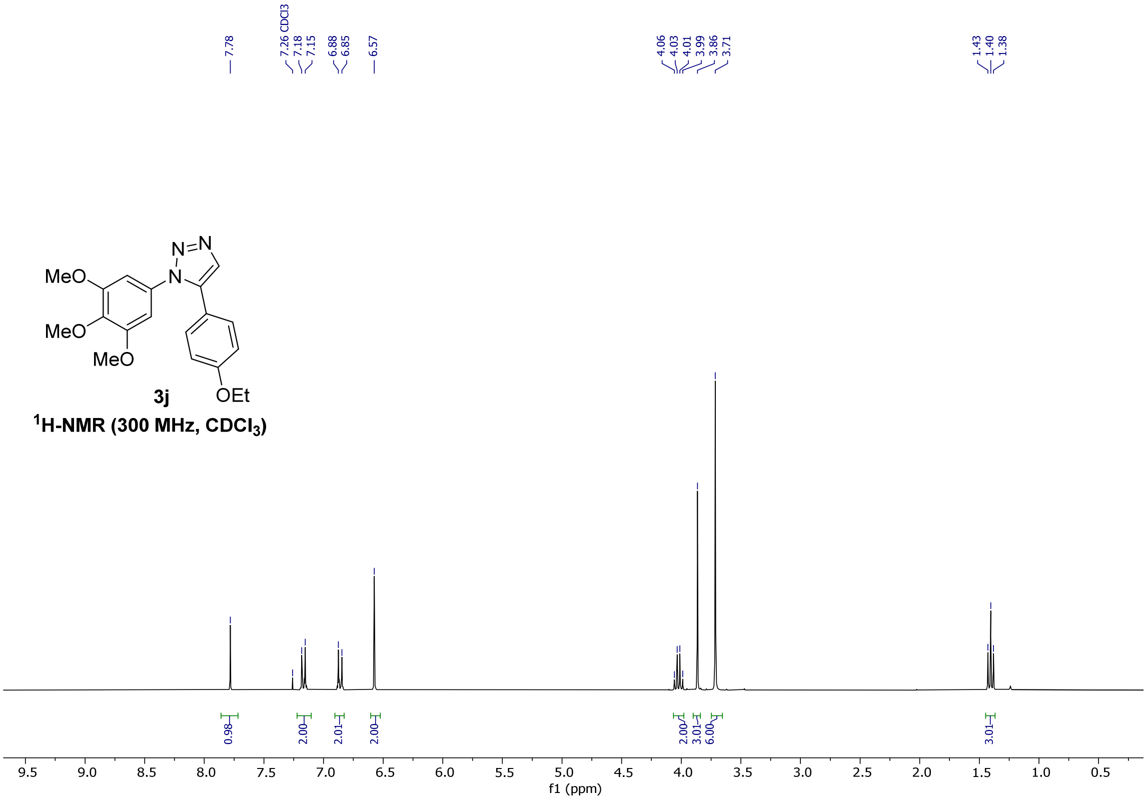
7.78
7.26 CDCl3
7.18
7.15
6.88
6.85
6.57
4.06
4.03
4.01
3.99
3.86
3.71
1.43
1.40
1.38
N=N
N
MeO
MeO
MeO
OEt
3j
^{1}H-NMR (300 MHz, CDCl$_3$)
0.98
2.00
2.01
2.00
2.00
3.01
6.00
3.01
f1 (ppm)

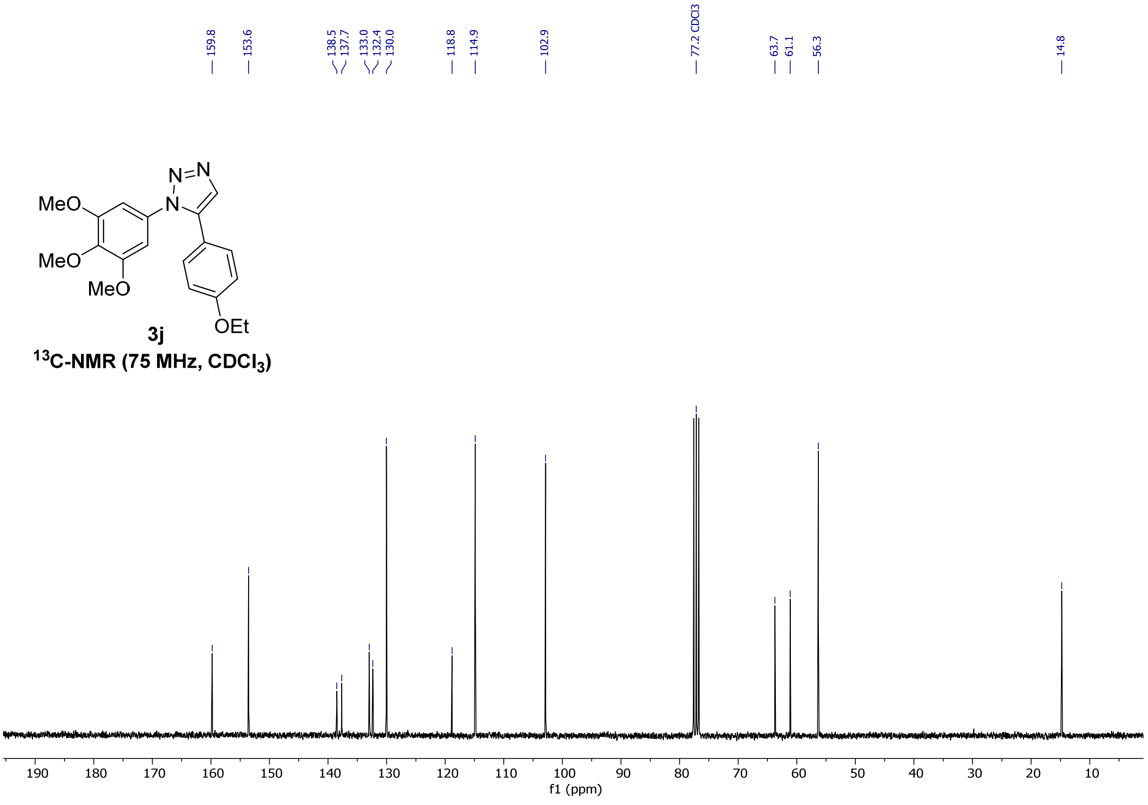
159.8
153.6
138.5
137.7
133.0
132.4
130.0
118.8
114.9
102.9
77.2 CDCl3
63.7
61.1
56.3
14.8
N=N
N
MeO
MeO
MeO
OEt
3j
^{13}C-NMR (75 MHz, CDCl$_3$)
f1 (ppm)

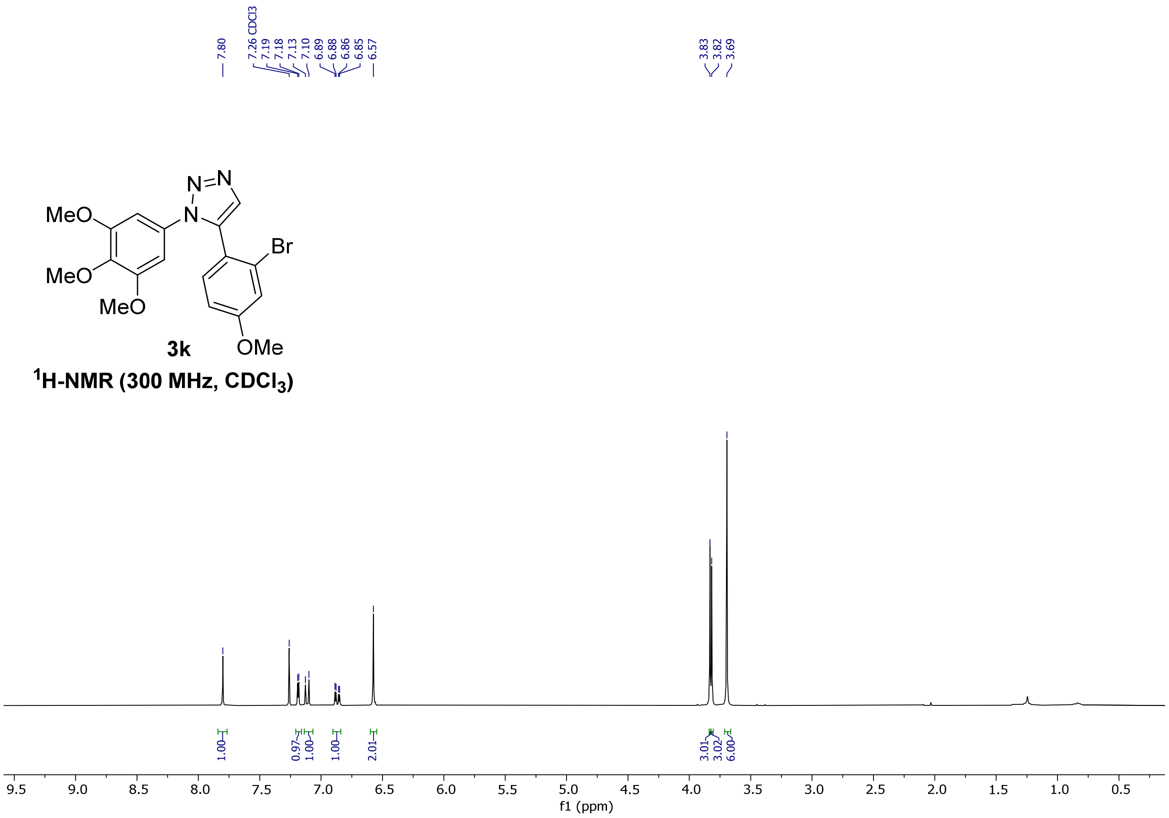
7.80
7.26 CDCl3
7.19
7.18
7.13
7.10
6.89
6.88
6.86
6.85
6.57
3.83
3.82
3.69
MeO
MeO
MeO
N
N
N
Br
OMe
3k
1H-NMR (300 MHz, CDCl3)
1.00
0.97
1.00
1.00
2.01
3.01
3.02
6.00
f1 (ppm)

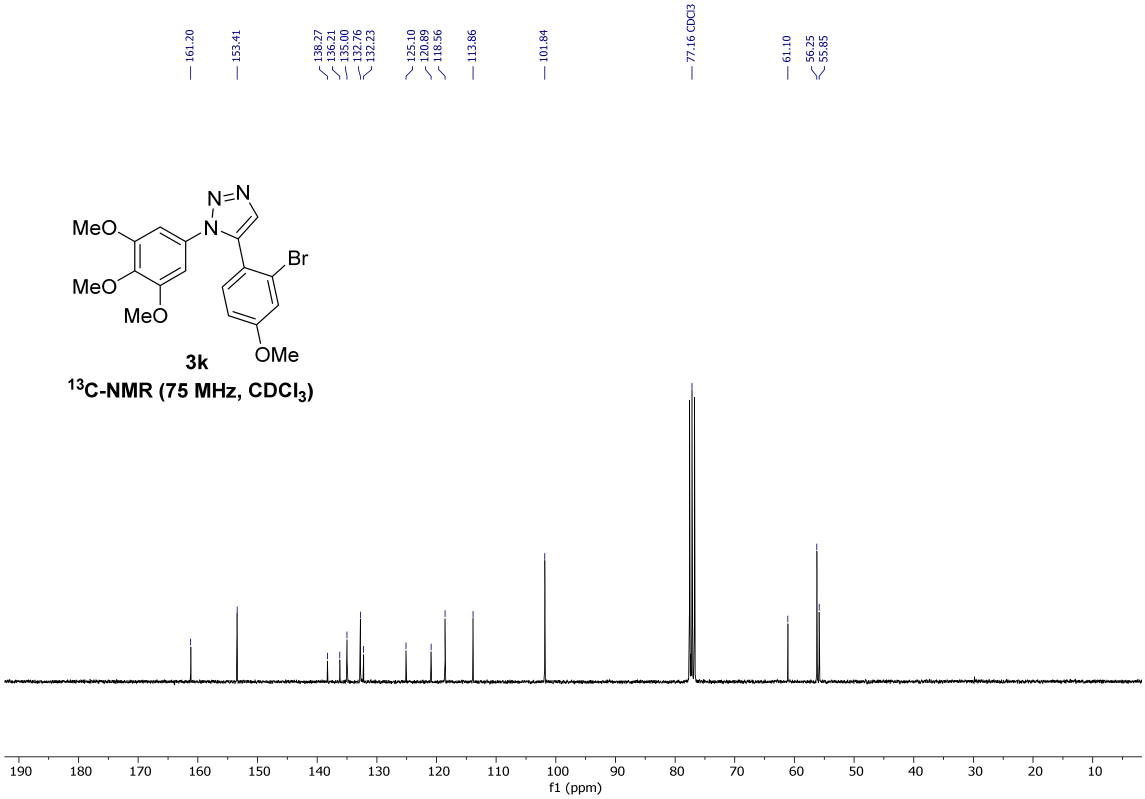
161.20
153.41
138.27
136.21
135.00
132.76
132.23
125.10
120.89
118.56
113.86
101.84
77.16 CDCl3
61.10
56.25
55.85
MeO
MeO
MeO
N
N
N
Br
OMe
3k
13C-NMR (75 MHz, CDCl3)
f1 (ppm)

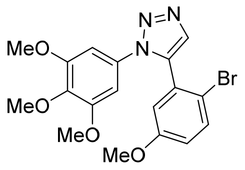

3l

^{1}H-NMR (300 MHz, $CDCl_3$)

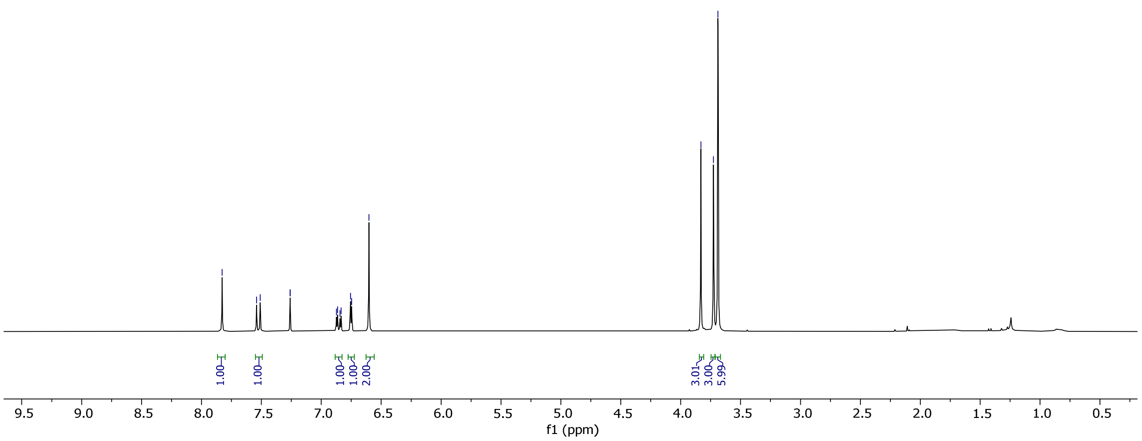

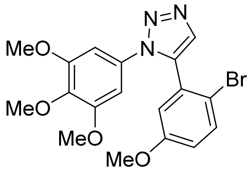

3l

^{13}C-NMR (75 MHz, $CDCl_3$)

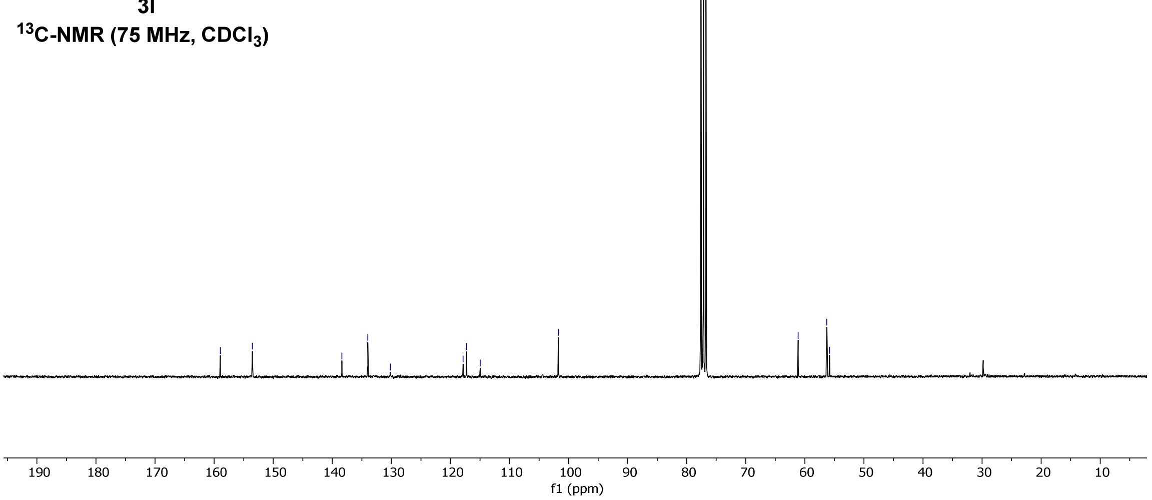

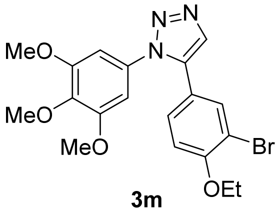

3m

^{1}H-NMR (400 MHz, $CDCl_3$)

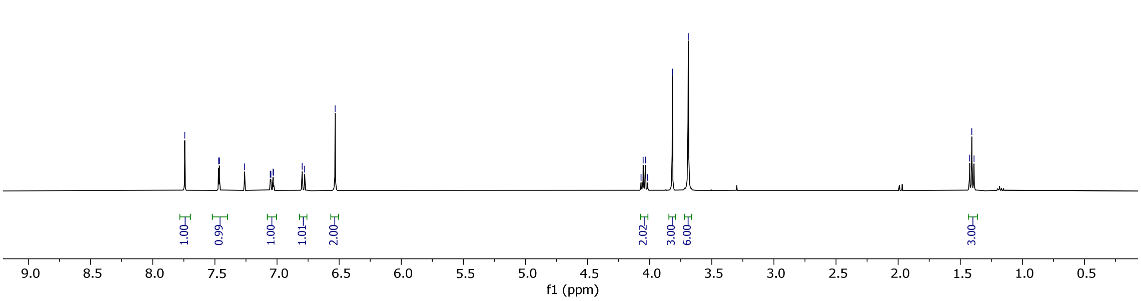

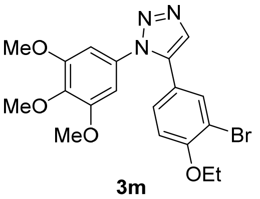

3m

^{13}C-NMR (101 MHz, $CDCl_3$)

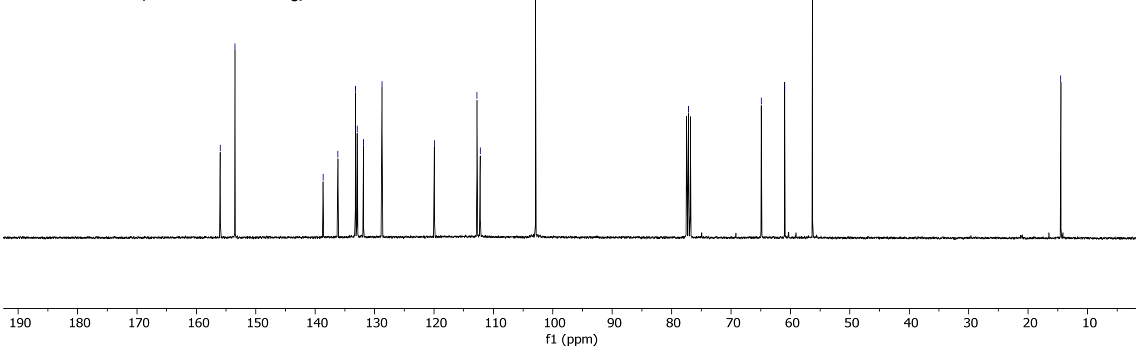

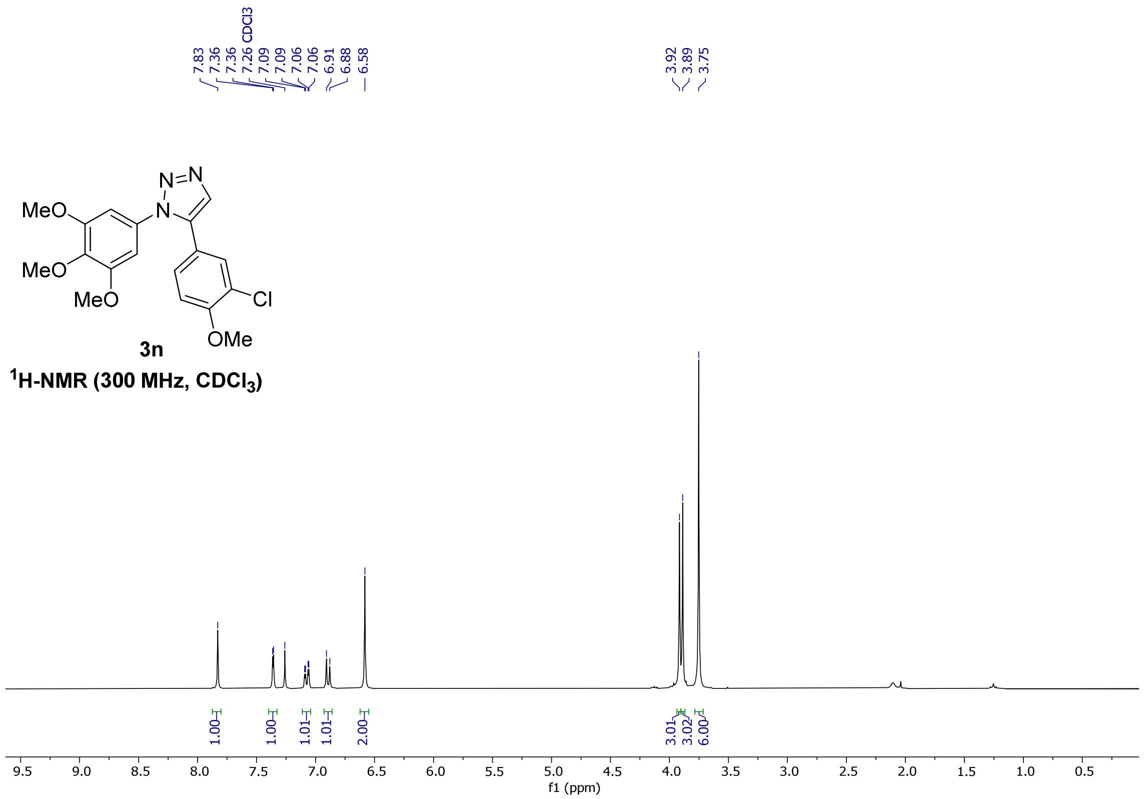
7.83
7.36
7.36
7.26 CDCl3
7.09
7.09
7.06
7.06
6.91
6.88
6.58
3.92
3.89
3.75
MeO
MeO
MeO
N=N
N
Cl
OMe
3n
1H-NMR (300 MHz, CDCl3)
1.00
1.00
1.01
1.01
2.00
3.01
3.02
6.00
f1 (ppm)

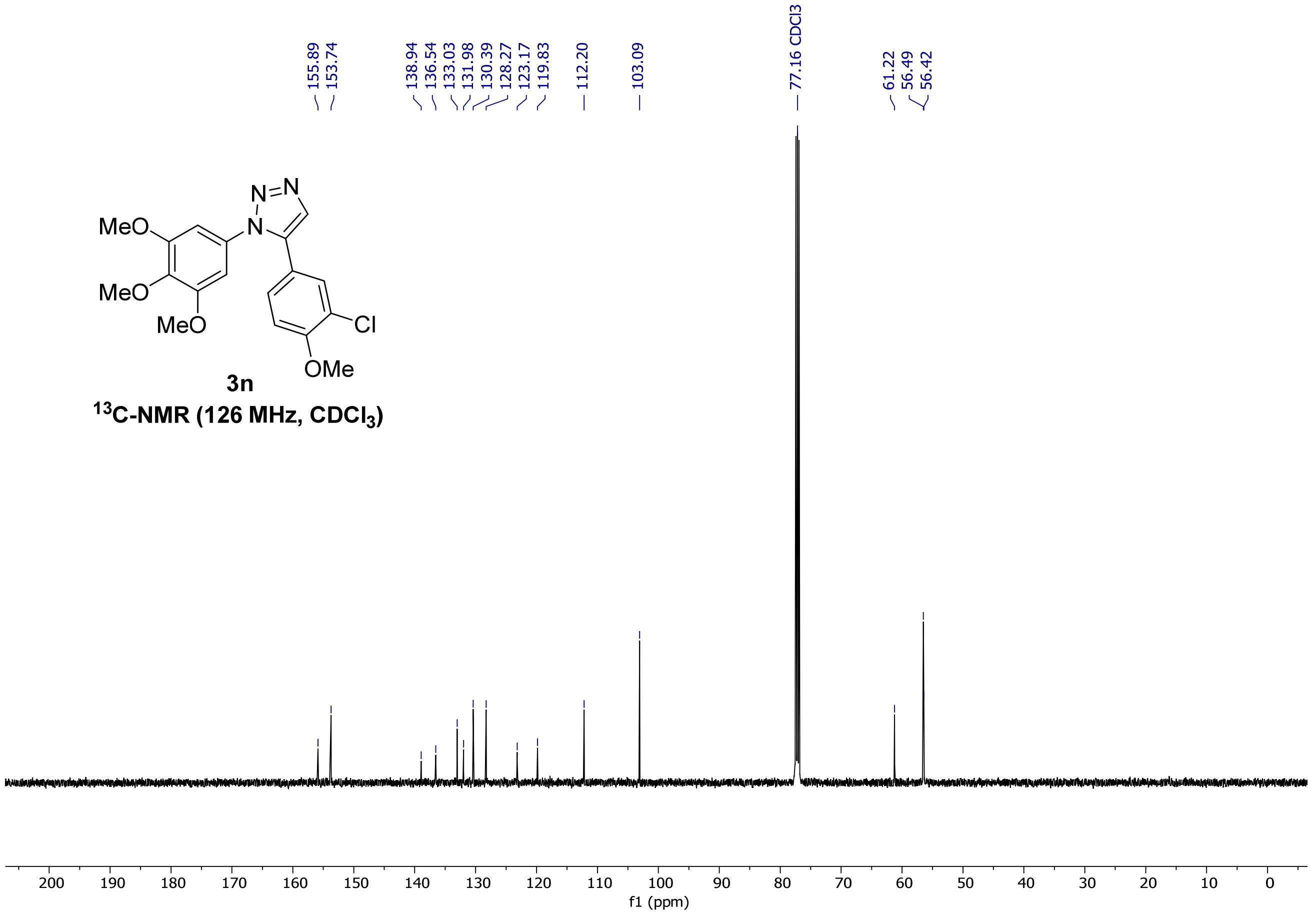
155.89
153.74
138.94
136.54
133.03
131.98
130.39
128.27
123.17
119.83
112.20
103.09
77.16 CDCl3
61.22
56.49
56.42
MeO
MeO
MeO
N=N
N
Cl
OMe
3n
13C-NMR (126 MHz, CDCl3)
f1 (ppm)

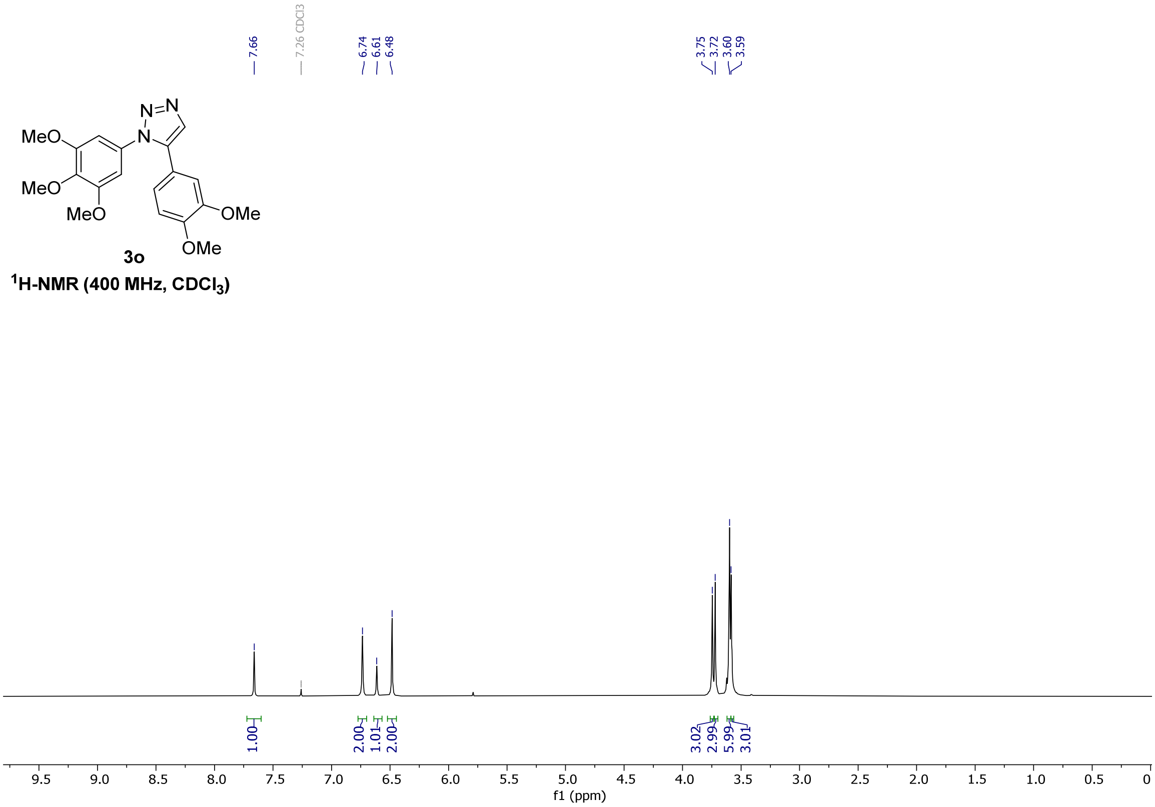
7.66
7.26 CDCl3
6.74
6.61
6.48
3.75
3.72
3.60
3.59
MeO
MeO
MeO
OMe
OMe
3o
1H-NMR (400 MHz, CDCl3)
1.00
2.00
1.01
2.00
3.02
2.99
5.99
3.01
f1 (ppm)

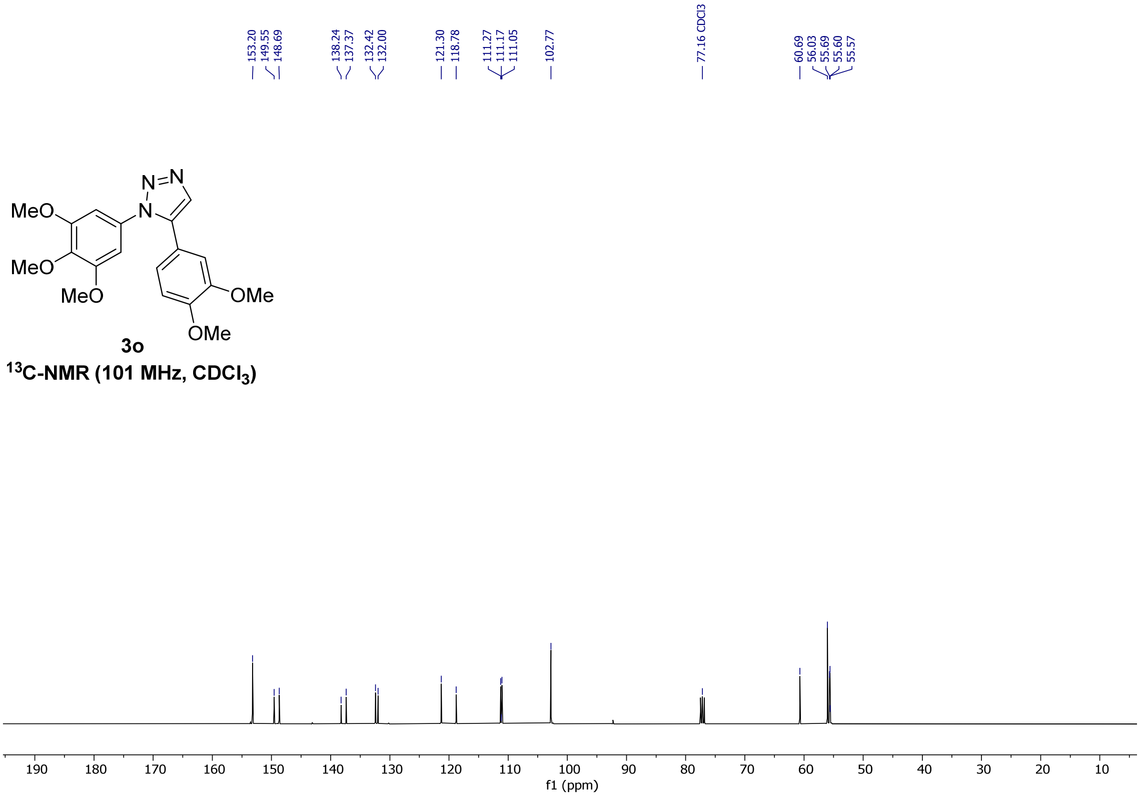
153.20
149.55
148.69
138.24
137.37
132.42
132.00
121.30
118.78
111.27
111.17
111.05
102.77
77.16 CDCl3
60.69
56.03
55.69
55.60
55.57
MeO
MeO
MeO
OMe
OMe
3o
13C-NMR (101 MHz, CDCl3)
f1 (ppm)

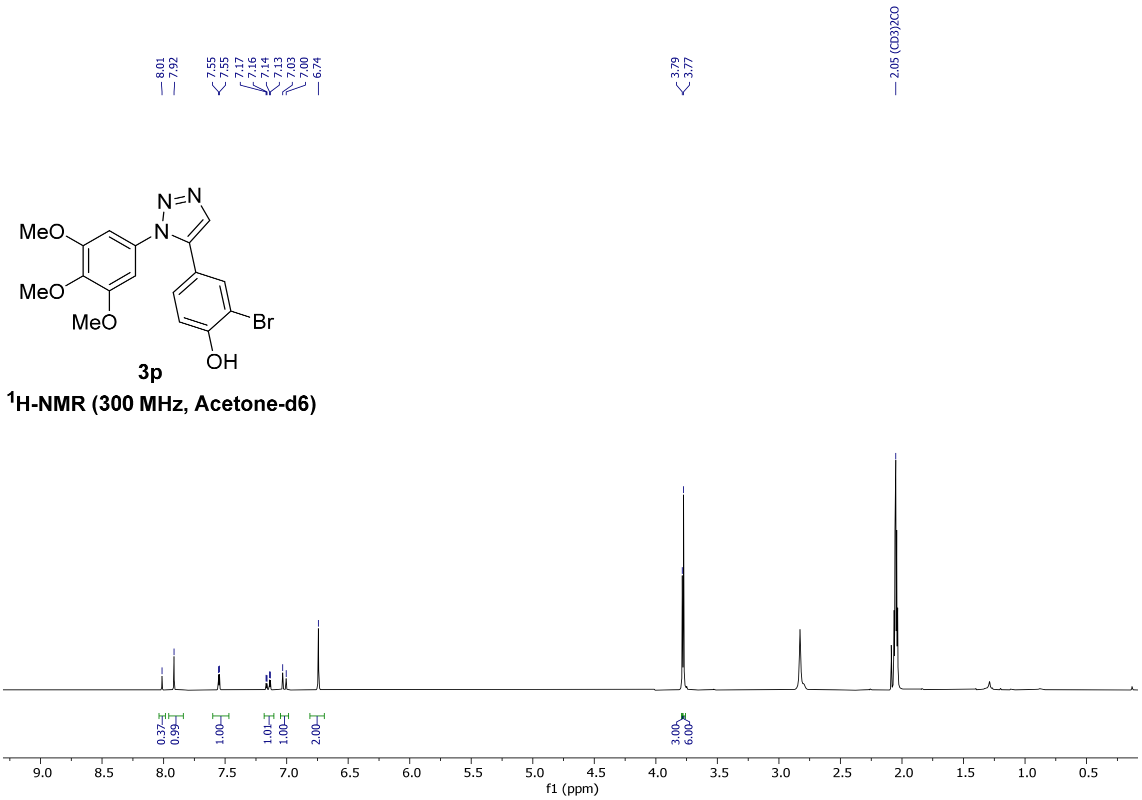
8.01
7.92
7.55
7.55
7.17
7.16
7.14
7.13
7.03
7.00
6.74
3.79
3.77
2.05 (CD3)2CO
MeO
MeO
MeO
N
N
N
Br
OH
3p
1H-NMR (300 MHz, Acetone-d6)
0.37
0.99
1.00
1.01
1.00
2.00
3.00
6.00
f1 (ppm)

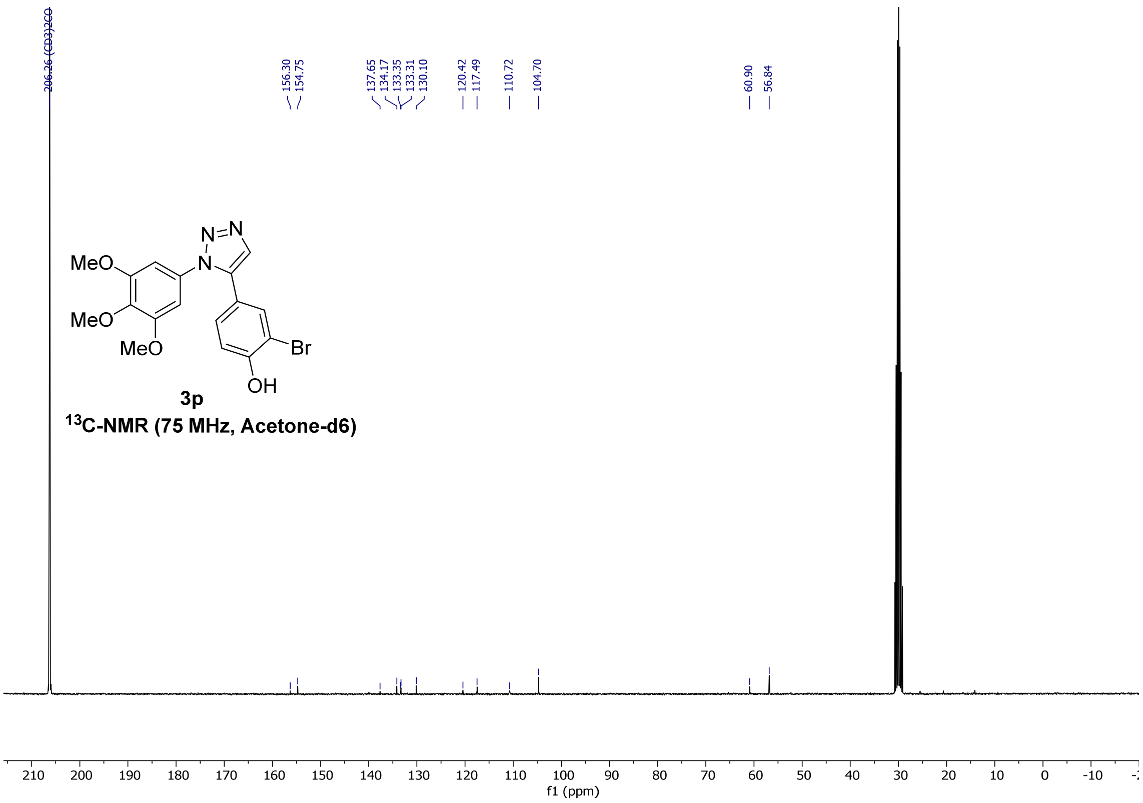
206.26 (CD3)2CO
156.30
154.75
137.65
134.17
133.35
133.31
130.10
120.42
117.49
110.72
104.70
60.90
56.84
MeO
MeO
MeO
N
N
N
Br
OH
3p
13C-NMR (75 MHz, Acetone-d6)
f1 (ppm)

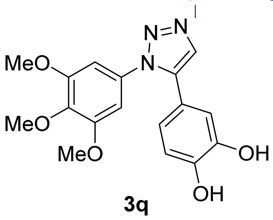

3q

^{1}H-NMR (300 MHz, CD_3OD)

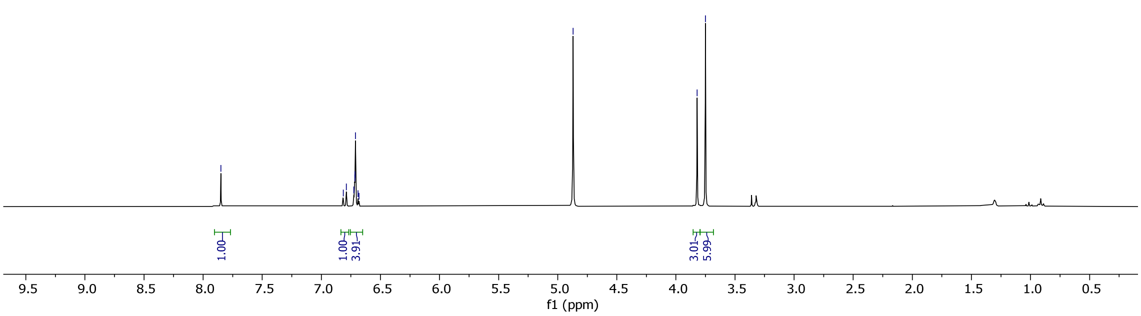

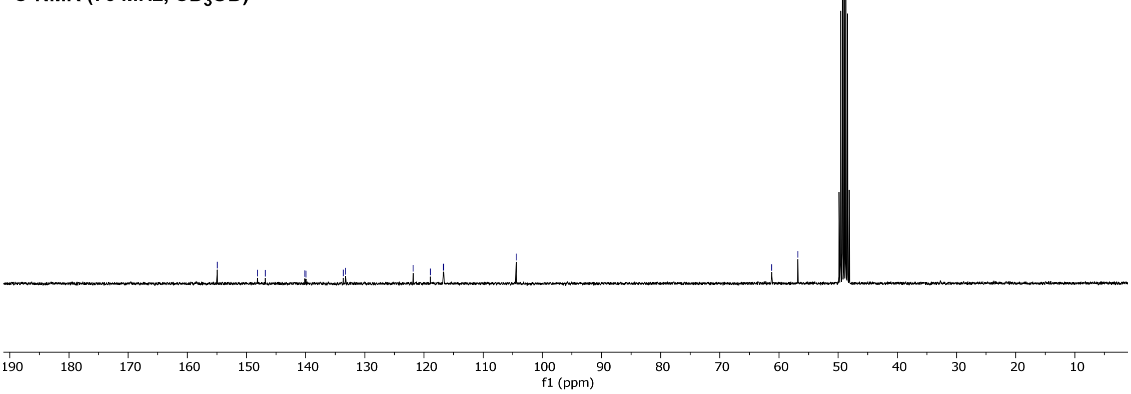

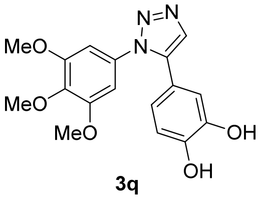

3q

^{13}C-NMR (75 MHz, CD_3OD)

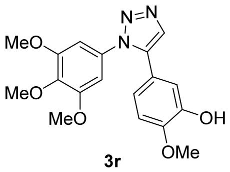

3r

^{1}H-NMR (300 MHz, $CDCl_3$)

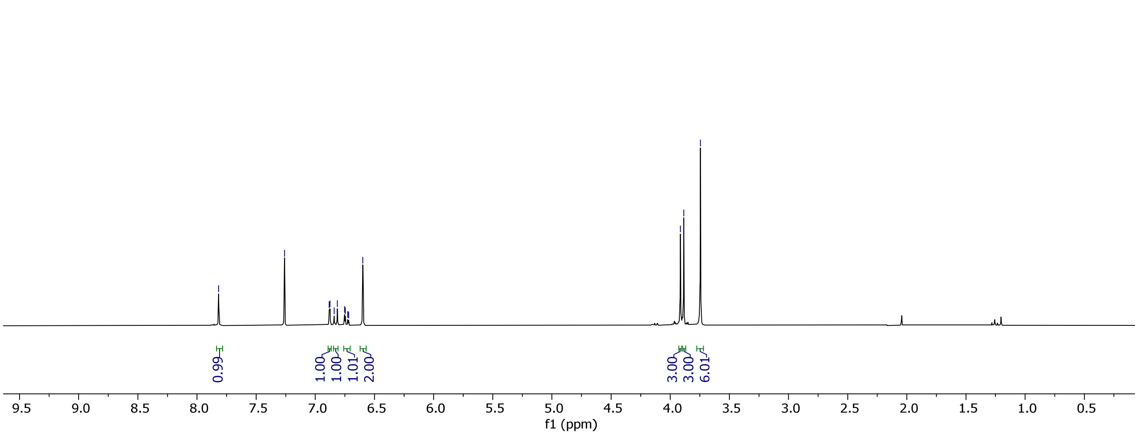

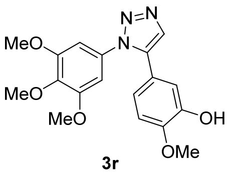

3r

^{13}C-NMR (75 MHz, $CDCl_3$)

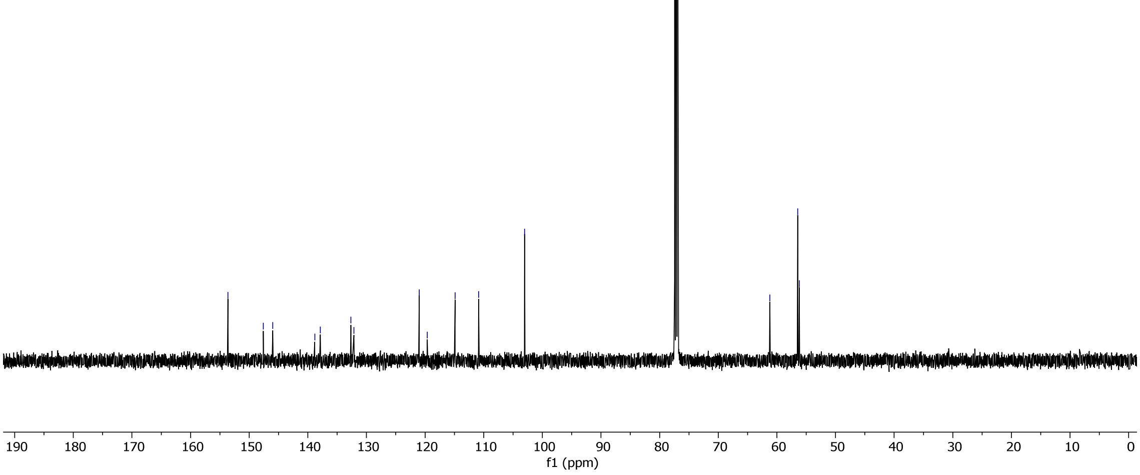

HRMS spectra of new compounds

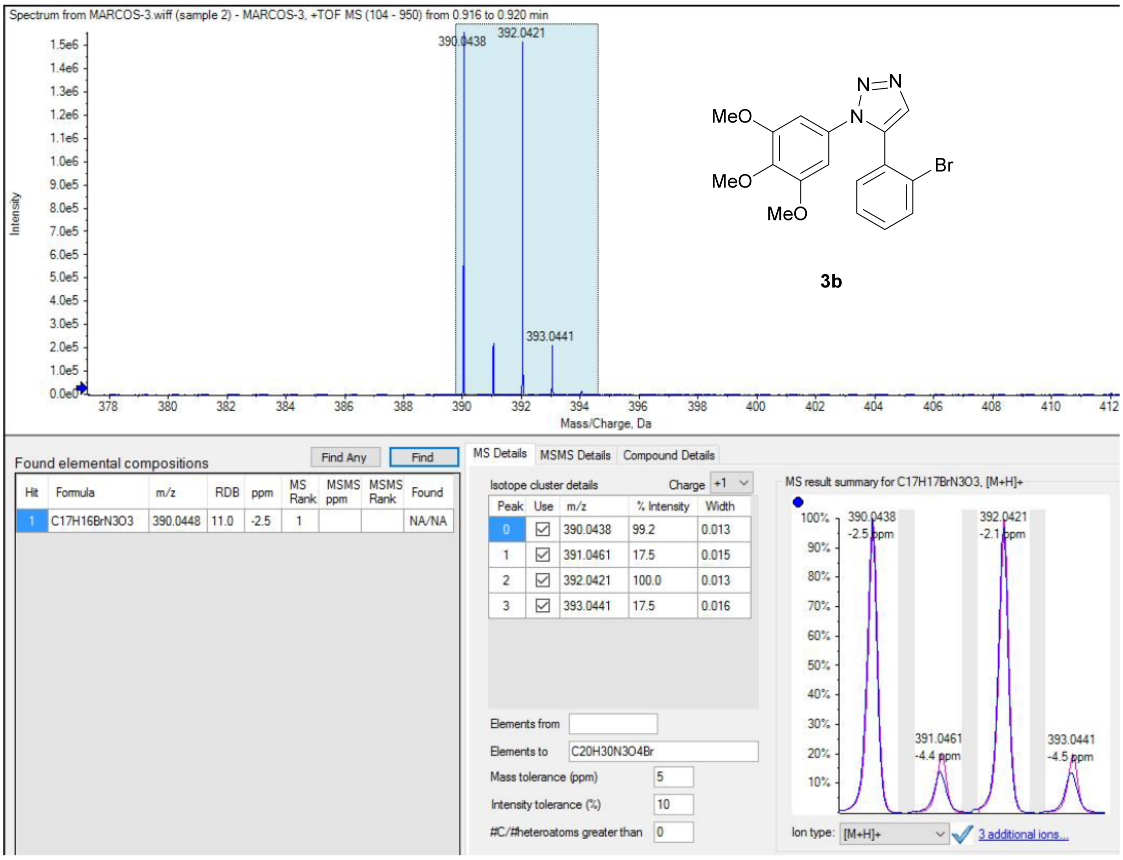

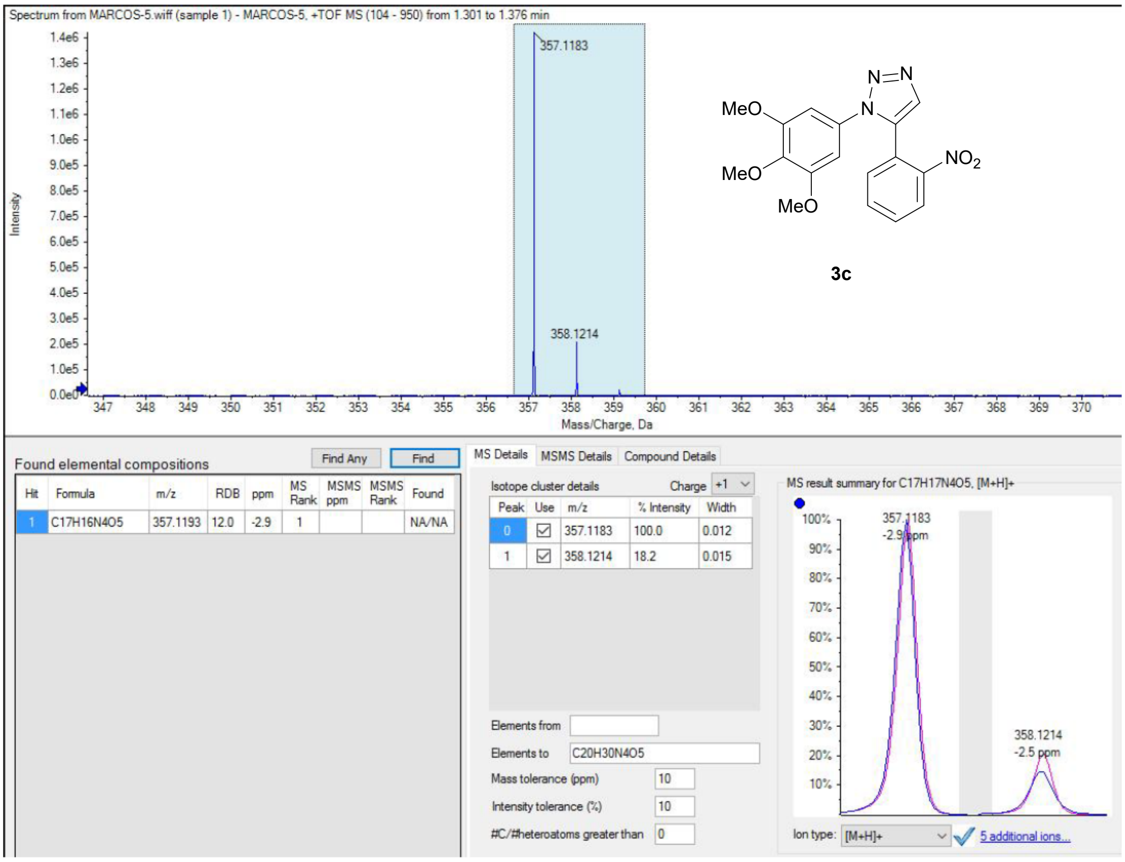

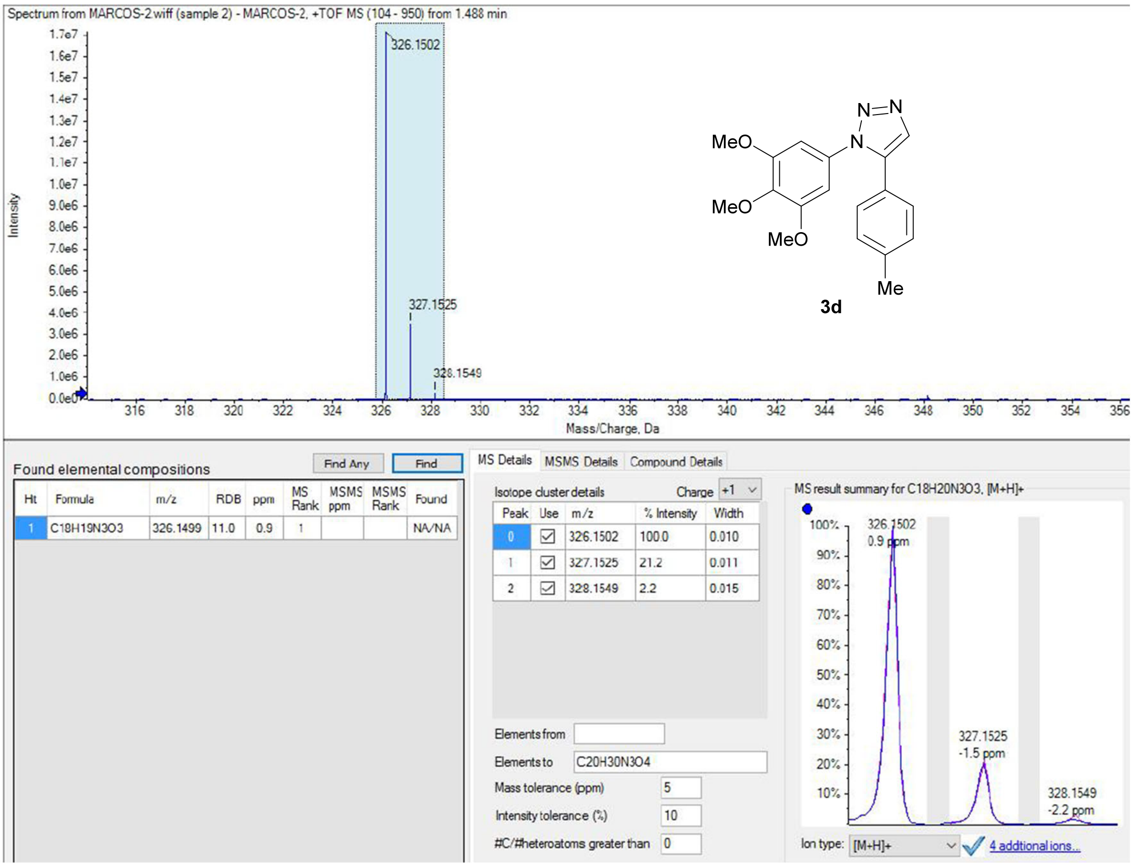
Spectrum from MARCOS-2.wiff (sample 2) - MARCOS-2, +TOF MS (104 - 950) from 1.488 min
326.1502
327.1525
328.1549
Intensity
Mass/Charge, Da
MeO
MeO
MeO
Me
3d
Found elemental compositions
Find Any
Find
Ht | Formula | m/z | RDB | ppm | MS Rank | MSMS ppm | MSMS Rank | Found
1 | C18H19N3O3 | 326.1499 | 11.0 | 0.9 | 1 | | | NA/NA
MS Details
MSMS Details
Compound Details
Isotope cluster details
Charge +1
Peak | Use | m/z | % Intensity | Width
0 | 326.1502 | 100.0 | 0.010
1 | 327.1525 | 21.2 | 0.011
2 | 328.1549 | 2.2 | 0.015
Elements from
Elements to C20H30N3O4
Mass tolerance (ppm) 5
Intensity tolerance (%) 10
#C/#heteroatoms greater than 0
MS result summary for C18H20N3O3, [M+H]+
326.1502
0.9 ppm
327.1525
-1.5 ppm
328.1549
-2.2 ppm
Ion type: [M+H]+
4 addtional ions...

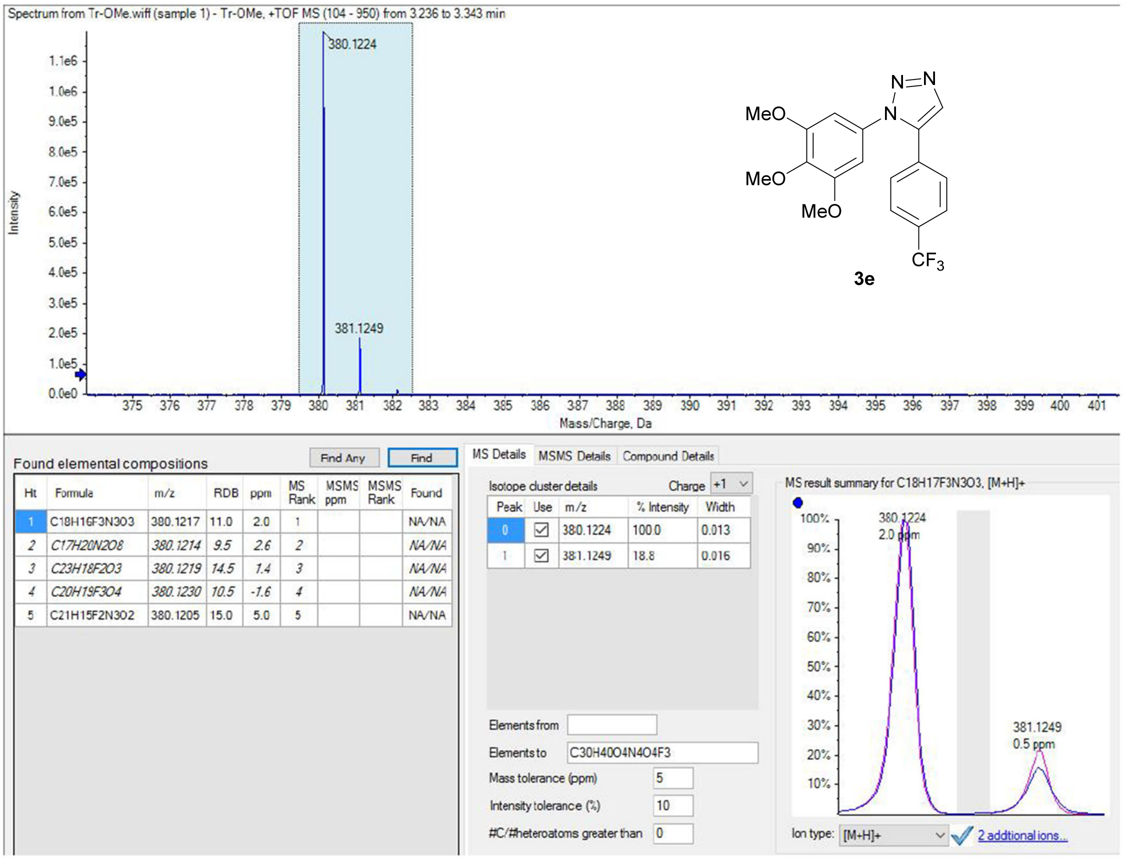
Spectrum from Tr-OMe.wiff (sample 1) - Tr-OMe, +TOF MS (104 - 950) from 3.236 to 3.343 min
380.1224
381.1249
Intensity
Mass/Charge, Da
MeO
MeO
MeO
CF3
3e
Found elemental compositions
Find Any
Find
Ht | Formula | m/z | RDB | ppm | MS Rank | MSMS ppm | MSMS Rank | Found
1 | C18H16F3N3O3 | 380.1217 | 11.0 | 2.0 | 1 | | | NA/NA
2 | C17H20N2O8 | 380.1214 | 9.5 | 2.6 | 2 | | | NA/NA
3 | C23H18F2O3 | 380.1219 | 14.5 | 1.4 | 3 | | | NA/NA
4 | C20H19F3O4 | 380.1230 | 10.5 | -1.6 | 4 | | | NA/NA
5 | C21H15F2N3O2 | 380.1205 | 15.0 | 5.0 | 5 | | | NA/NA
MS Details
MSMS Details
Compound Details
Isotope cluster details
Charge +1
Peak | Use | m/z | % Intensity | Width
0 | 380.1224 | 100.0 | 0.013
1 | 381.1249 | 18.8 | 0.016
Elements from
Elements to C30H40O4N4O4F3
Mass tolerance (ppm) 5
Intensity tolerance (%) 10
#C/#heteroatoms greater than 0
MS result summary for C18H17F3N3O3, [M+H]+
380.1224
2.0 ppm
381.1249
0.5 ppm
Ion type: [M+H]+
2 addtional ions...

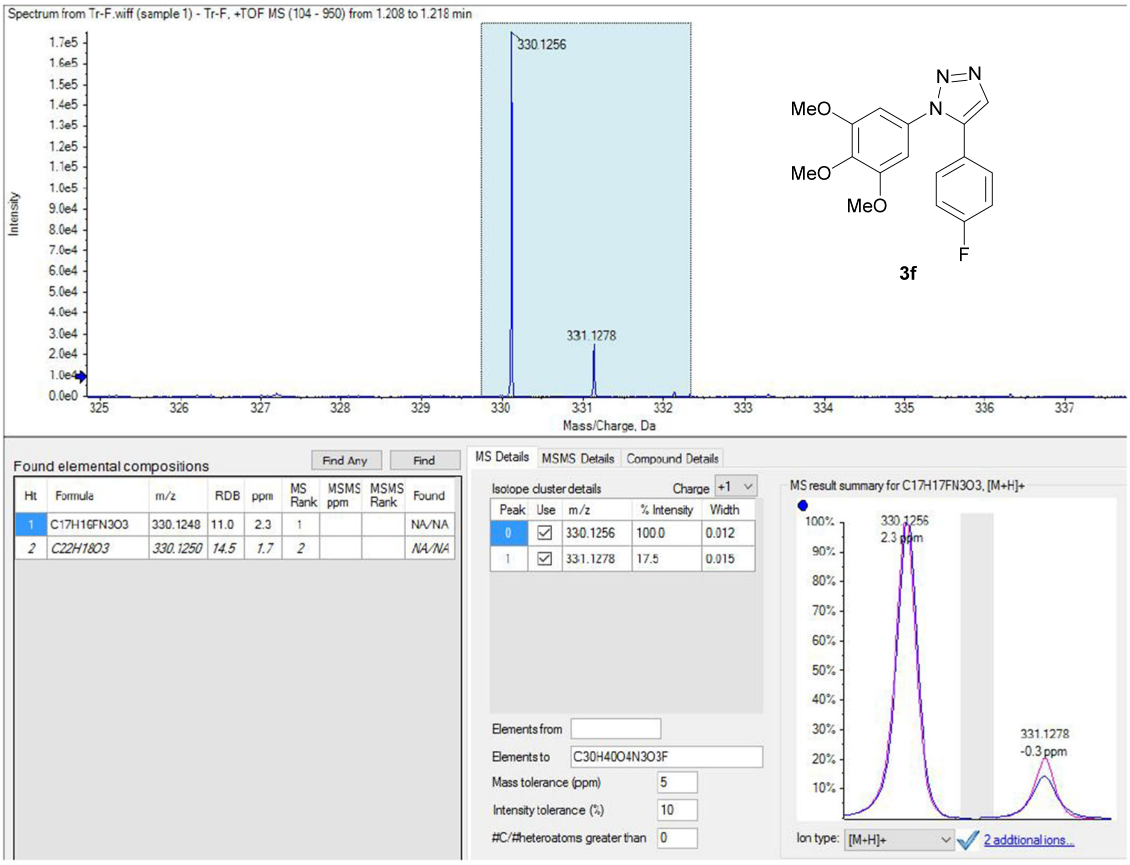
Spectrum from Tr-F.wiff (sample 1) - Tr-F, +TOF MS (104 - 950) from 1.208 to 1.218 min
330.1256
331.1278
Intensity
Mass/Charge, Da
MeO
MeO
MeO
N
N
N
F
Found elemental compositions
Find Any
Find
Ht | Formula | m/z | RDB | ppm | MS Rank | MSMS ppm | MSMS Rank | Found
1 | C17H16FN3O3 | 330.1248 | 11.0 | 2.3 | 1 | | | NA/NA
2 | C22H18O3 | 330.1250 | 14.5 | 1.7 | 2 | | | NA/NA
MS Details
MSMS Details
Compound Details
Isotope cluster details
Charge +1
Peak | Use | m/z | % Intensity | Width
0 | | 330.1256 | 100.0 | 0.012
1 | | 331.1278 | 17.5 | 0.015
Elements from
Elements to C30H40O4N3O3F
Mass tolerance (ppm) 5
Intensity tolerance (%) 10
#C/#heteroatoms greater than 0
MS result summary for C17H17FN3O3, [M+H]+
330.1256
2.3 ppm
331.1278
-0.3 ppm
Ion type: [M+H]+
2 addtional ions...

3f

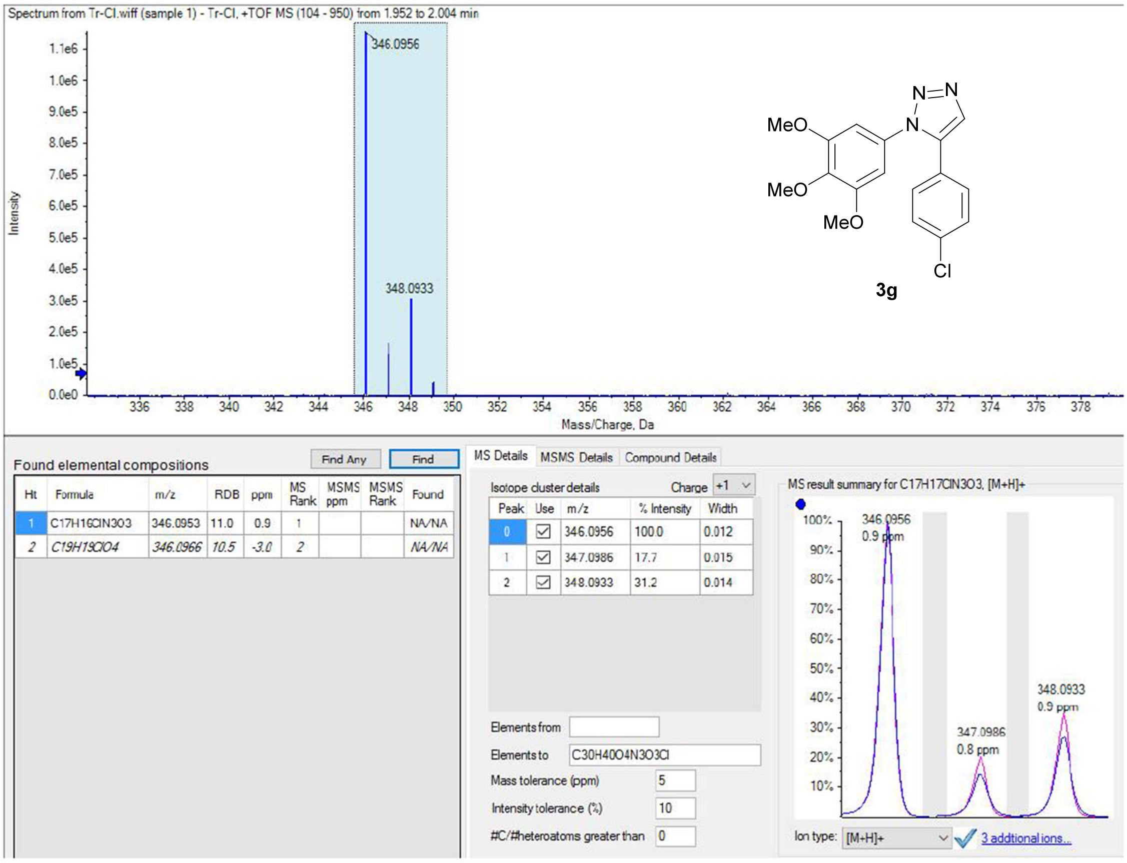
Spectrum from Tr-Cl.wiff (sample 1) - Tr-Cl, +TOF MS (104 - 950) from 1.952 to 2.004 min
346.0956
348.0933
Intensity
Mass/Charge, Da
MeO
MeO
MeO
N
N
N
Cl
Found elemental compositions
Find Any
Find
Ht | Formula | m/z | RDB | ppm | MS Rank | MSMS ppm | MSMS Rank | Found
1 | C17H16ClN3O3 | 346.0953 | 11.0 | 0.9 | 1 | | | NA/NA
2 | C19H19ClO4 | 346.0966 | 10.5 | -3.0 | 2 | | | NA/NA
MS Details
MSMS Details
Compound Details
Isotope cluster details
Charge +1
Peak | Use | m/z | % Intensity | Width
0 | | 346.0956 | 100.0 | 0.012
1 | | 347.0986 | 17.7 | 0.015
2 | | 348.0933 | 31.2 | 0.014
Elements from
Elements to C30H40O4N3O3Cl
Mass tolerance (ppm) 5
Intensity tolerance (%) 10
#C/#heteroatoms greater than 0
MS result summary for C17H17ClN3O3, [M+H]+
346.0956
0.9 ppm
347.0986
0.8 ppm
348.0933
0.9 ppm
Ion type: [M+H]+
3 addtional ions...

3g

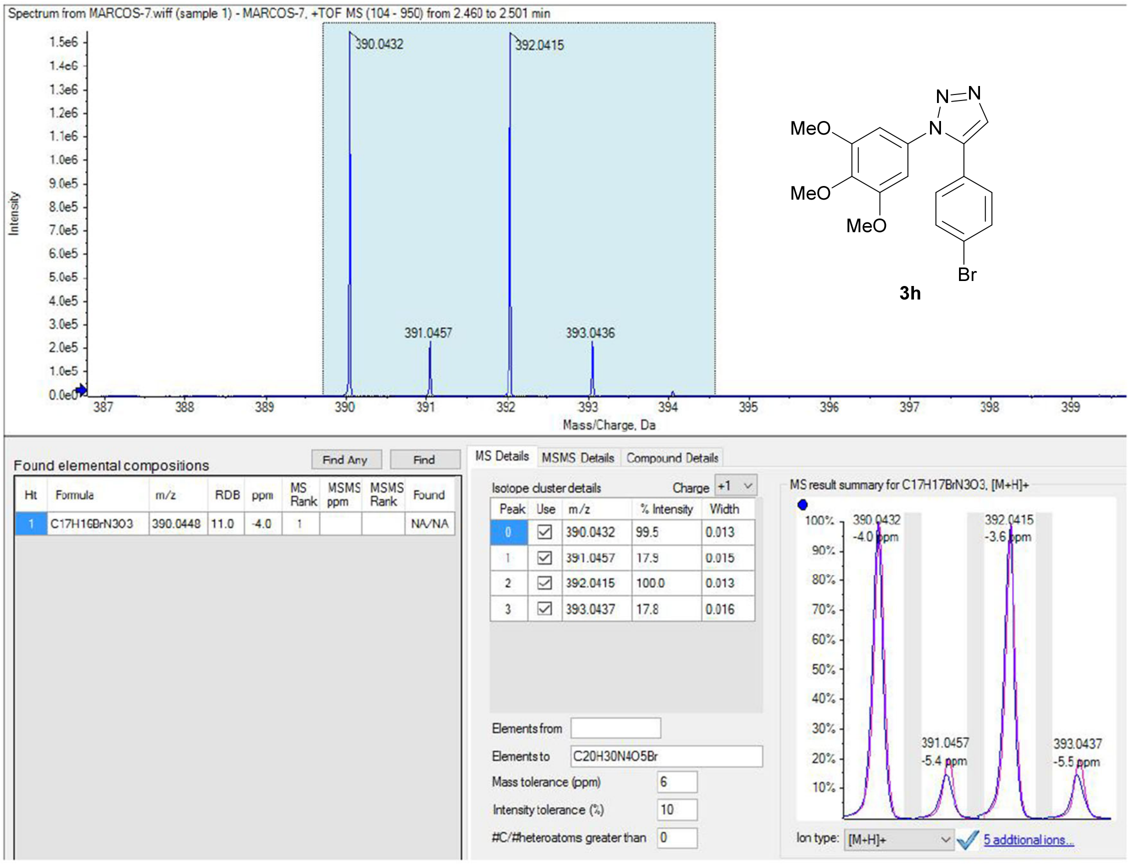

Spectrum from MARCOS-7.wiff (sample 1) - MARCOS-7, +TOF MS (104 - 950) from 2.460 to 2.501 min
390.0432
392.0415
391.0457
393.0436
Intensity
Mass/Charge, Da
MeO
MeO
MeO
N=N
N
Br
3h
Found elemental compositions
Find Any
Find
Ht | Formula | m/z | RDB | ppm | MS Rank | MSMS ppm | MSMS Rank | Found
1 | C17H16BrN3O3 | 390.0448 | 11.0 | -4.0 | 1 | | | NA/NA
MS Details
MSMS Details
Compound Details
Isotope cluster details
Charge +1
Peak | Use | m/z | % Intensity | Width
0 | 390.0432 | 99.5 | 0.013
1 | 391.0457 | 17.9 | 0.015
2 | 392.0415 | 100.0 | 0.013
3 | 393.0437 | 17.8 | 0.016
Elements from
Elements to C20H30N4O5Br
Mass tolerance (ppm) 6
Intensity tolerance (%) 10
#C/#heteroatoms greater than 0
MS result summary for C17H17BrN3O3, [M+H]+
390.0432 -4.0 ppm
392.0415 -3.6 ppm
391.0457 -5.4 ppm
393.0437 -5.5 ppm
Ion type: [M+H]+
5 addtional ions...

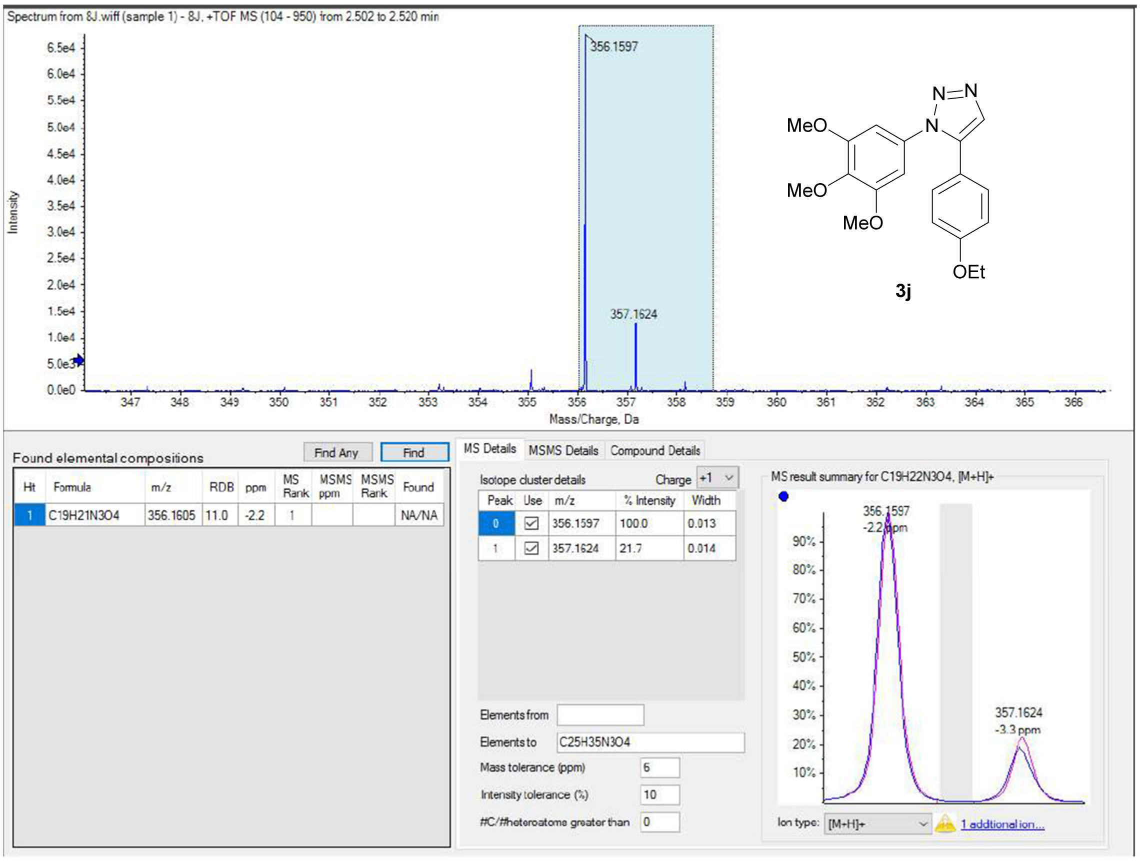

Spectrum from 8J.wiff (sample 1) - 8J, +TOF MS (104 - 950) from 2.502 to 2.520 min
356.1597
357.1624
Intensity
Mass/Charge, Da
MeO
MeO
MeO
N=N
N
OEt
3j
Found elemental compositions
Find Any
Find
Ht | Formula | m/z | RDB | ppm | MS Rank | MSMS ppm | MSMS Rank | Found
1 | C19H21N3O4 | 356.1605 | 11.0 | -2.2 | 1 | | | NA/NA
MS Details
MSMS Details
Compound Details
Isotope cluster details
Charge +1
Peak | Use | m/z | % Intensity | Width
0 | 356.1597 | 100.0 | 0.013
1 | 357.1624 | 21.7 | 0.014
Elements from
Elements to C25H35N3O4
Mass tolerance (ppm) 6
Intensity tolerance (%) 10
#C/#heteroatoms greater than 0
MS result summary for C19H22N3O4, [M+H]+
356.1597 -2.2 ppm
357.1624 -3.3 ppm
Ion type: [M+H]+
1 addtional ion...

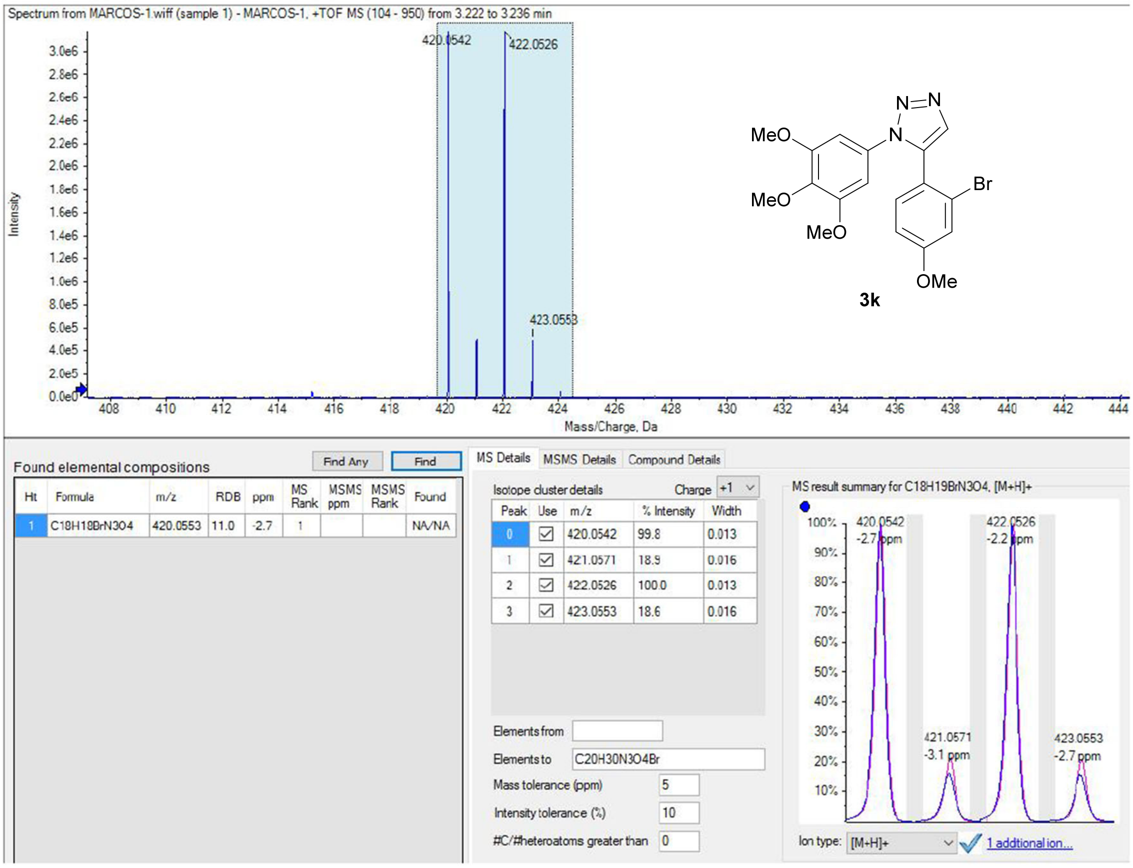
Spectrum from MARCOS-1.wiff (sample 1) - MARCOS-1, +TOF MS (104 - 950) from 3.222 to 3.236 min
420.0542
422.0526
423.0553
Intensity
Mass/Charge, Da
MeO
MeO
MeO
N
N=N
Br
OMe
3k
Found elemental compositions
Find Any
Find
Ht Formula m/z RDB ppm MS Rank MSMS ppm MSMS Rank Found
1 C18H18BrN3O4 420.0553 11.0 -2.7 1 NA/NA
MS Details
MSMS Details
Compound Details
Isotope cluster details
Charge +1
Peak Use m/z % Intensity Width
0 420.0542 99.8 0.013
1 421.0571 18.9 0.016
2 422.0526 100.0 0.013
3 423.0553 18.6 0.016
Elements from
Elements to C20H30N3O4Br
Mass tolerance (ppm) 5
Intensity tolerance (%) 10
#C/#heteroatoms greater than 0
MS result summary for C18H19BrN3O4, [M+H]+
420.0542 -2.7 ppm
422.0526 -2.2 ppm
421.0571 -3.1 ppm
423.0553 -2.7 ppm
Ion type: [M+H]+
1 addtional ion...

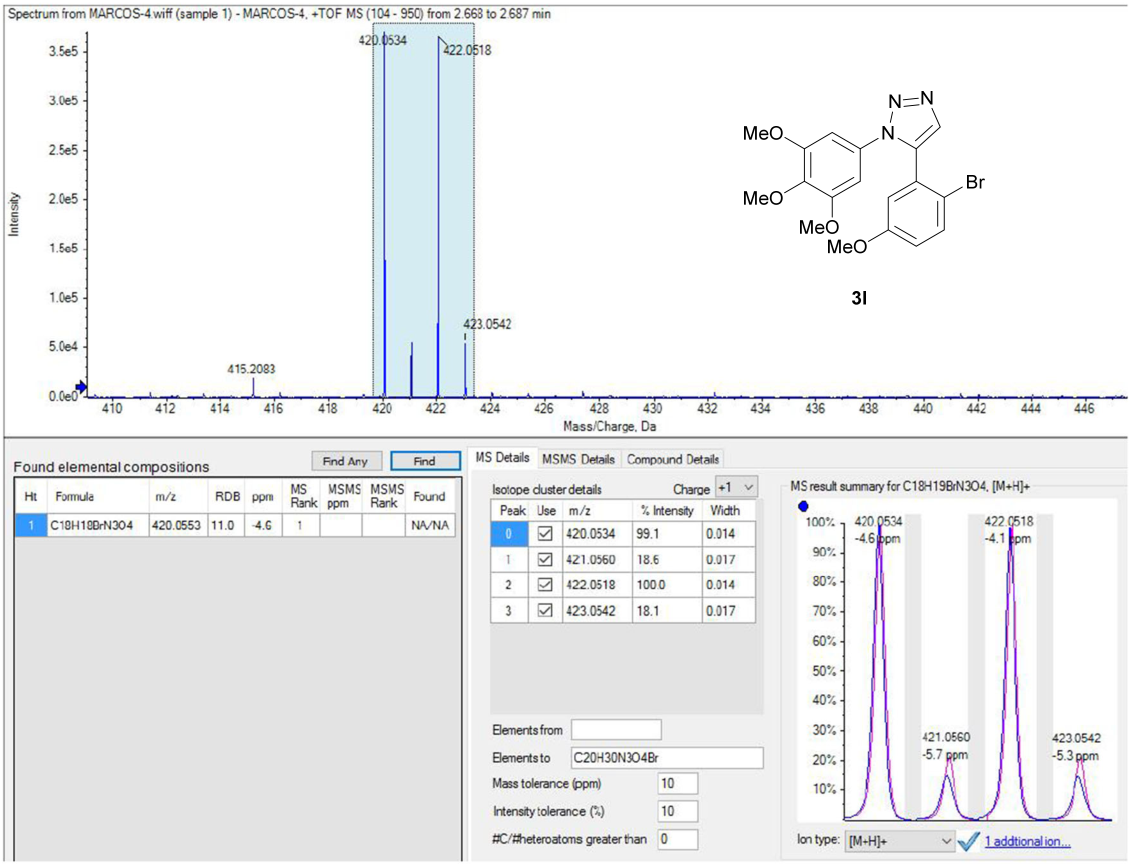
Spectrum from MARCOS-4.wiff (sample 1) - MARCOS-4, +TOF MS (104 - 950) from 2.668 to 2.687 min
420.0534
422.0518
423.0542
415.2083
Intensity
Mass/Charge, Da
MeO
MeO
MeO
MeO
N
N=N
Br
3l
Found elemental compositions
Find Any
Find
Ht Formula m/z RDB ppm MS Rank MSMS ppm MSMS Rank Found
1 C18H18BrN3O4 420.0553 11.0 -4.6 1 NA/NA
MS Details
MSMS Details
Compound Details
Isotope cluster details
Charge +1
Peak Use m/z % Intensity Width
0 420.0534 99.1 0.014
1 421.0560 18.6 0.017
2 422.0518 100.0 0.014
3 423.0542 18.1 0.017
Elements from
Elements to C20H30N3O4Br
Mass tolerance (ppm) 10
Intensity tolerance (%) 10
#C/#heteroatoms greater than 0
MS result summary for C18H19BrN3O4, [M+H]+
420.0534 -4.6 ppm
422.0518 -4.1 ppm
421.0560 -5.7 ppm
423.0542 -5.3 ppm
Ion type: [M+H]+
1 addtional ion...

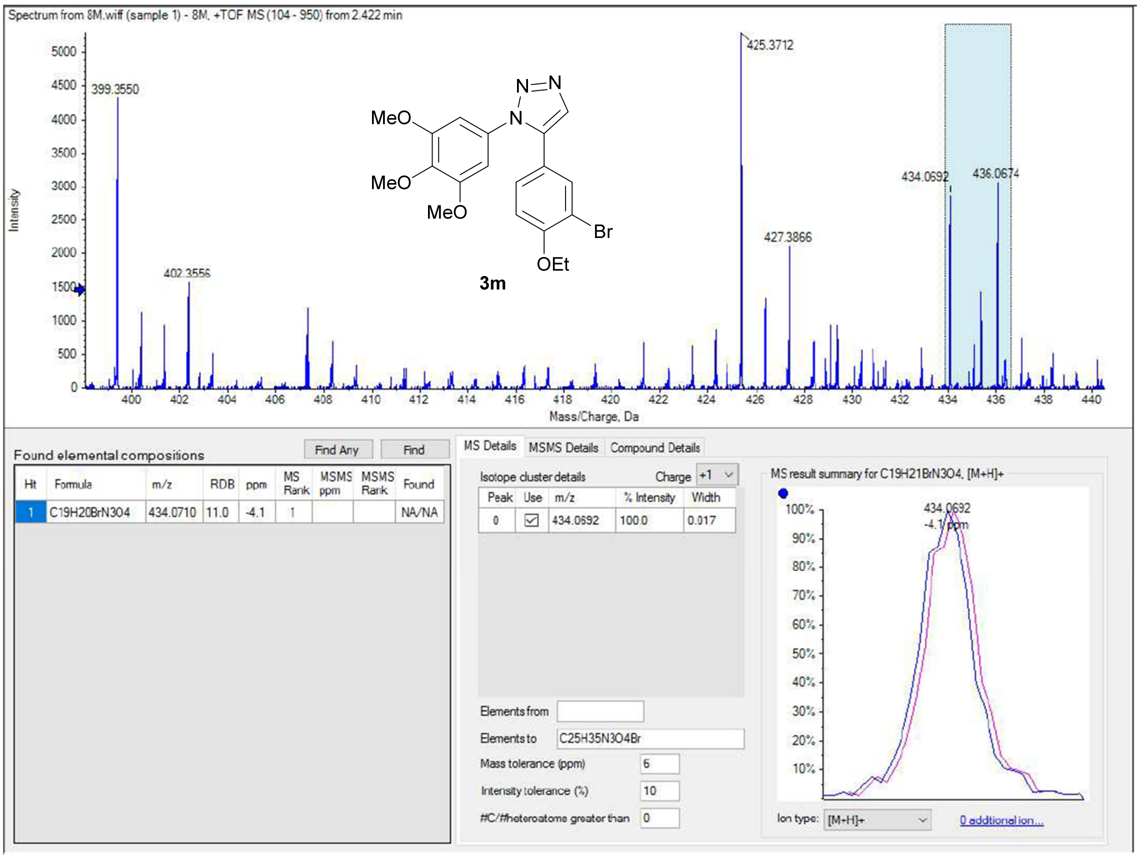
Spectrum from 8M.wiff (sample 1) - 8M, +TOF MS (104 - 950) from 2.422 min
Intensity
Mass/Charge, Da
399.3550
402.3556
425.3712
427.3866
434.0692
436.0674
MeO
MeO
MeO
N=N
N
Br
OEt
3m
Found elemental compositions
Find Any
Find
Ht | Formula | m/z | RDB | ppm | MS Rank | MSMS ppm | MSMS Rank | Found
1 | C19H20BrN3O4 | 434.0710 | 11.0 | -4.1 | 1 | | | NA/NA
MS Details
MSMS Details
Compound Details
Isotope cluster details
Charge +1
Peak | Use | m/z | % Intensity | Width
0 | 434.0692 | 100.0 | 0.017
Elements from
Elements to C25H35N3O4Br
Mass tolerance (ppm) 6
Intensity tolerance (%) 10
#C/#heteroatoms greater than 0
MS result summary for C19H21BrN3O4, [M+H]+
434.0692
-4.1 ppm
Ion type: [M+H]+
0 additional ion...

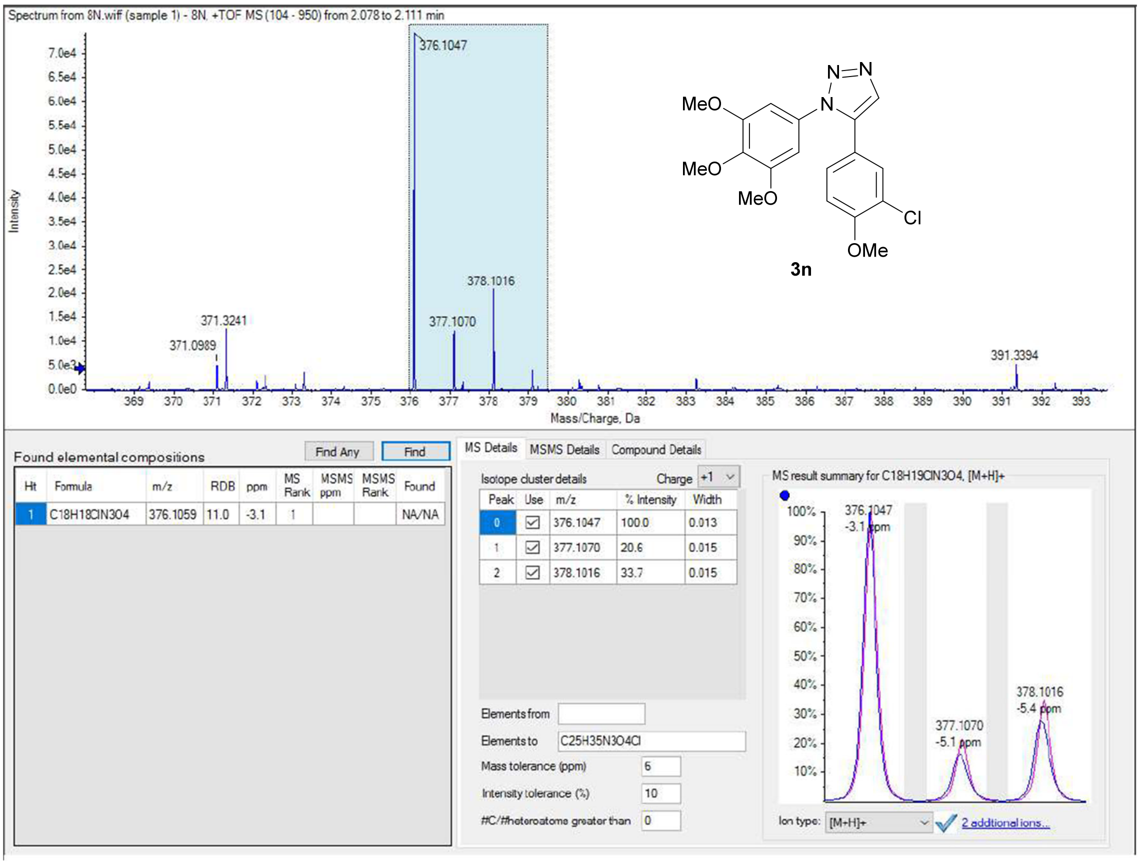
Spectrum from 8N.wiff (sample 1) - 8N, +TOF MS (104 - 950) from 2.078 to 2.111 min
Intensity
Mass/Charge, Da
376.1047
378.1016
377.1070
371.3241
371.0989
391.3394
MeO
MeO
MeO
N=N
N
Cl
OMe
3n
Found elemental compositions
Find Any
Find
Ht | Formula | m/z | RDB | ppm | MS Rank | MSMS ppm | MSMS Rank | Found
1 | C18H18ClN3O4 | 376.1059 | 11.0 | -3.1 | 1 | | | NA/NA
MS Details
MSMS Details
Compound Details
Isotope cluster details
Charge +1
Peak | Use | m/z | % Intensity | Width
0 | 376.1047 | 100.0 | 0.013
1 | 377.1070 | 20.6 | 0.015
2 | 378.1016 | 33.7 | 0.015
Elements from
Elements to C25H35N3O4Cl
Mass tolerance (ppm) 6
Intensity tolerance (%) 10
#C/#heteroatoms greater than 0
MS result summary for C18H19ClN3O4, [M+H]+
376.1047
-3.1 ppm
377.1070
-5.1 ppm
378.1016
-5.4 ppm
Ion type: [M+H]+
2 additional ions...

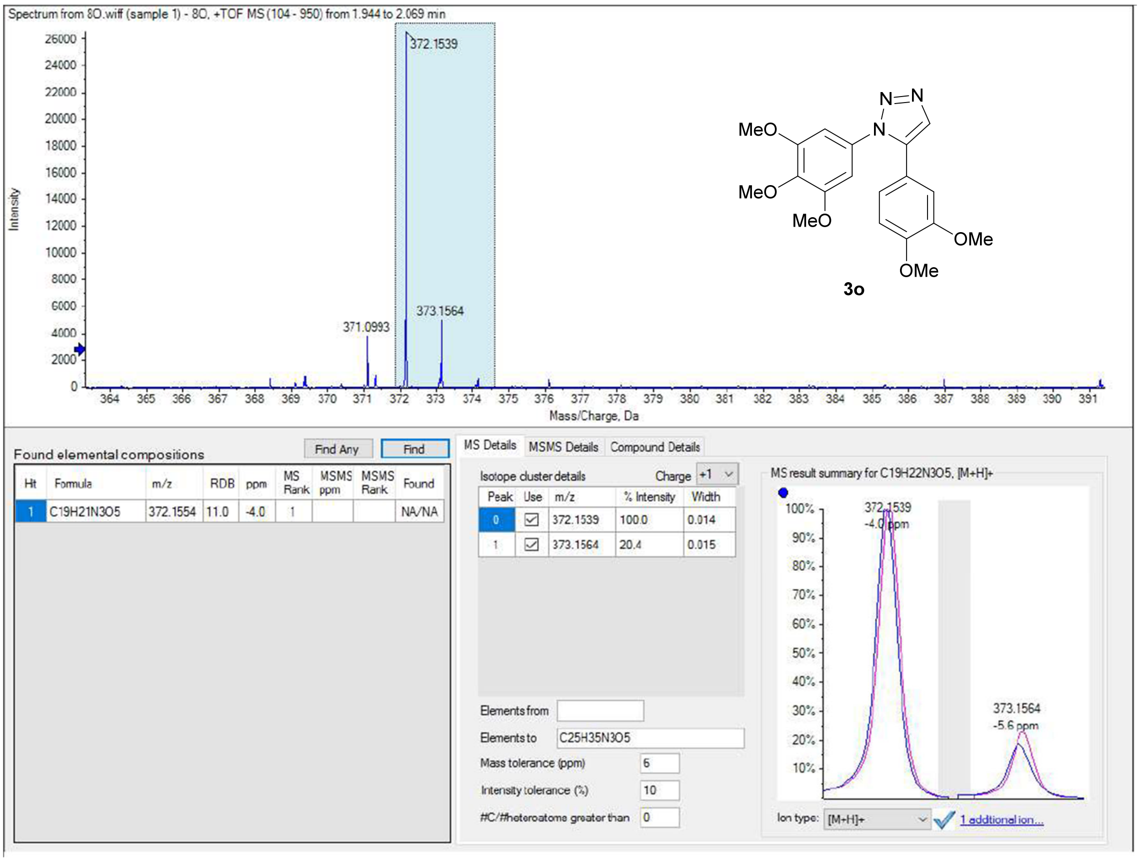
Spectrum from 8O.wiff (sample 1) - 8O, +TOF MS (104 - 950) from 1.944 to 2.069 min
372.1539
371.0993
373.1564
Intensity
Mass/Charge, Da
MeO
MeO
MeO
N=N
N
OMe
OMe
3o
Found elemental compositions
Find Any
Find
Ht | Formula | m/z | RDB | ppm | MS Rank | MSMS ppm | MSMS Rank | Found
1 | C19H21N3O5 | 372.1554 | 11.0 | -4.0 | 1 | | | NA/NA
MS Details
MSMS Details
Compound Details
Isotope cluster details
Charge +1
Peak | Use | m/z | % Intensity | Width
0 | 372.1539 | 100.0 | 0.014
1 | 373.1564 | 20.4 | 0.015
Elements from
Elements to C25H35N3O5
Mass tolerance (ppm) 6
Intensity tolerance (%) 10
#C/#heteroatoms greater than 0
MS result summary for C19H22N3O5, [M+H]+
372.1539
-4.0 ppm
373.1564
-5.6 ppm
Ion type: [M+H]+
1 addtional ion...

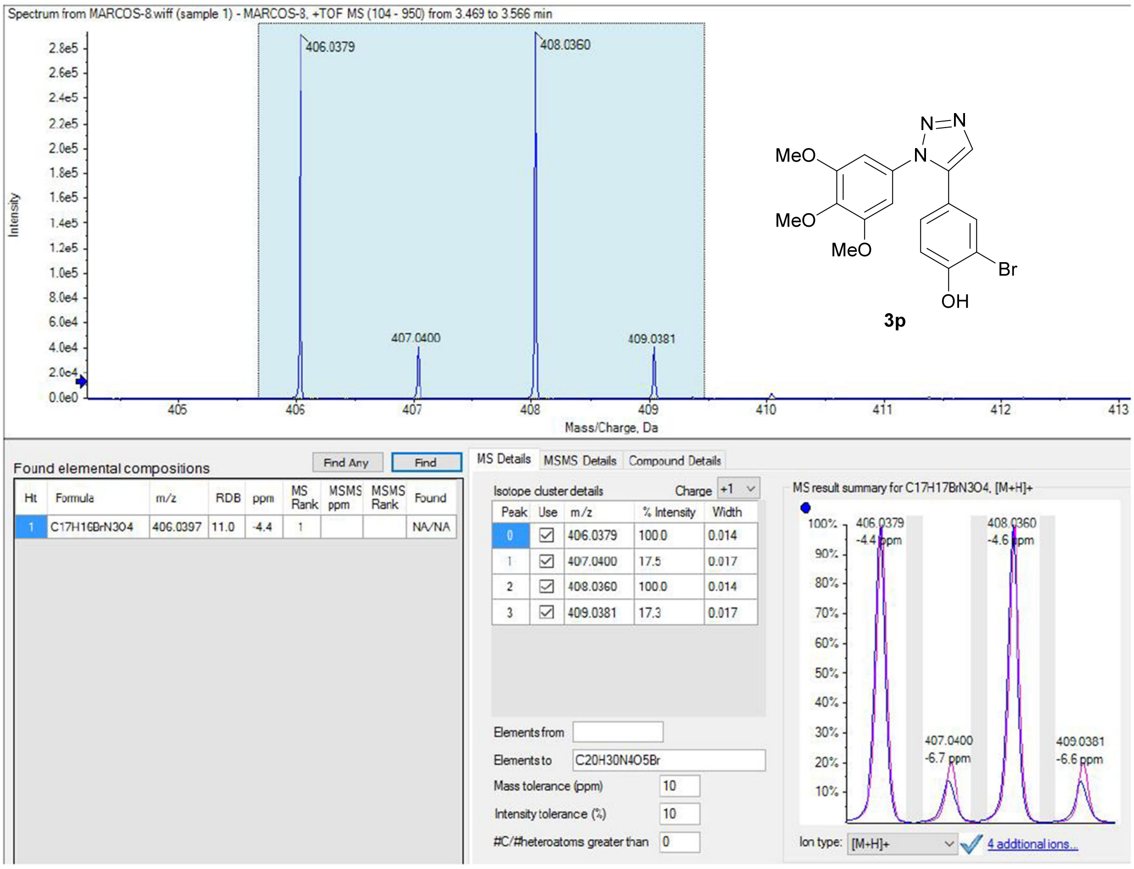
Spectrum from MARCOS-8.wiff (sample 1) - MARCOS-8, +TOF MS (104 - 950) from 3.469 to 3.566 min
406.0379
408.0360
407.0400
409.0381
Intensity
Mass/Charge, Da
MeO
MeO
MeO
N=N
N
Br
OH
3p
Found elemental compositions
Find Any
Find
Ht | Formula | m/z | RDB | ppm | MS Rank | MSMS ppm | MSMS Rank | Found
1 | C17H16BrN3O4 | 406.0397 | 11.0 | -4.4 | 1 | | | NA/NA
MS Details
MSMS Details
Compound Details
Isotope cluster details
Charge +1
Peak | Use | m/z | % Intensity | Width
0 | 406.0379 | 100.0 | 0.014
1 | 407.0400 | 17.5 | 0.017
2 | 408.0360 | 100.0 | 0.014
3 | 409.0381 | 17.3 | 0.017
Elements from
Elements to C20H30N4O5Br
Mass tolerance (ppm) 10
Intensity tolerance (%) 10
#C/#heteroatoms greater than 0
MS result summary for C17H17BrN3O4, [M+H]+
406.0379
-4.4 ppm
408.0360
-4.6 ppm
407.0400
-6.7 ppm
409.0381
-6.6 ppm
Ion type: [M+H]+
4 addtional ions...

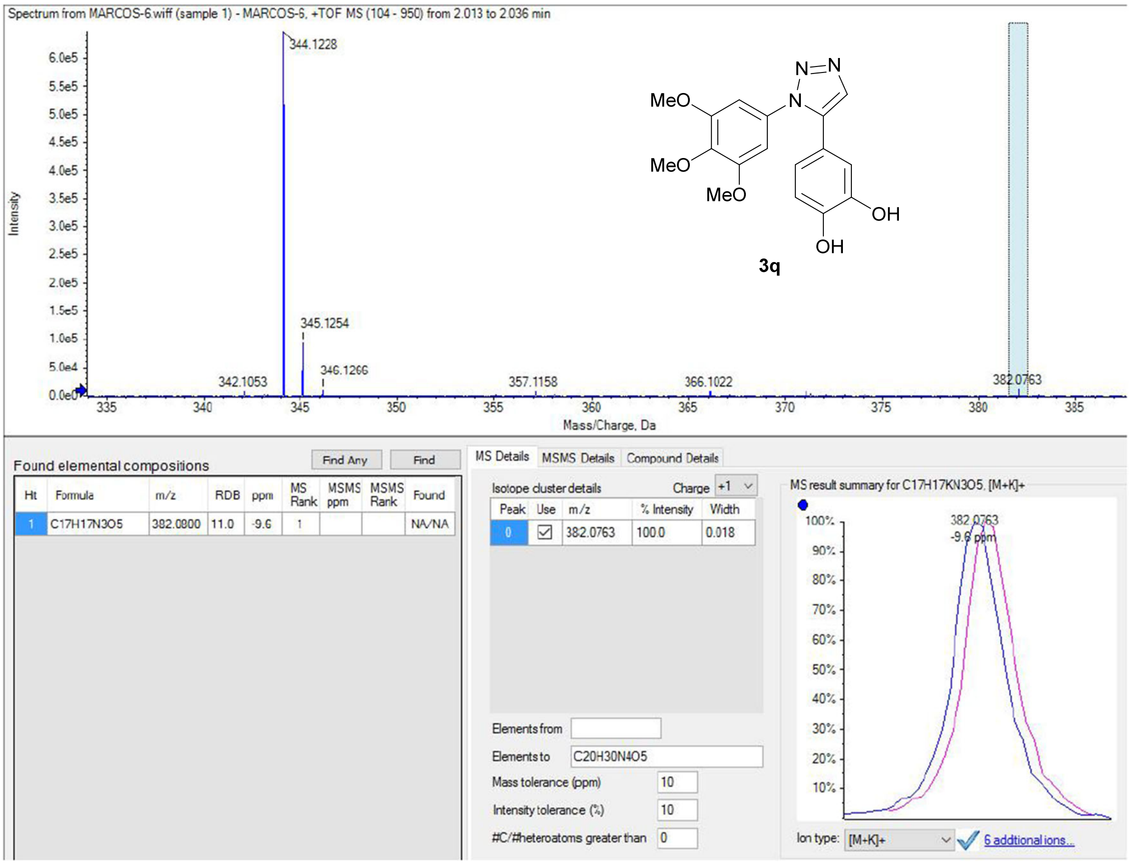
Spectrum from MARCOS-6.wiff (sample 1) - MARCOS-6, +TOF MS (104 - 950) from 2.013 to 2.036 min
344.1228
345.1254
346.1266
342.1053
357.1158
366.1022
382.0763
Intensity
Mass/Charge, Da
MeO
MeO
MeO
N=N
N
OH
OH
3q
Found elemental compositions
Find Any
Find
Ht | Formula | m/z | RDB | ppm | MS Rank | MSMS ppm | MSMS Rank | Found
1 | C17H17N3O5 | 382.0800 | 11.0 | -9.6 | 1 | | | NA/NA
MS Details
MSMS Details
Compound Details
Isotope cluster details
Charge +1
Peak | Use | m/z | % Intensity | Width
0 | | 382.0763 | 100.0 | 0.018
Elements from
Elements to C20H30N4O5
Mass tolerance (ppm) 10
Intensity tolerance (%) 10
#C/#heteroatoms greater than 0
MS result summary for C17H17KN3O5, [M+K]+
382.0763
-9.6 ppm
Ion type: [M+K]+
6 addtional ions...
